# Prognostic and diagnostic values of non-coding RNAs as biomarkers for breast cancer: An umbrella review and pan-cancer analysis

**DOI:** 10.3389/fmolb.2023.1096524

**Published:** 2023-01-16

**Authors:** Afshin Bahramy, Narges Zafari, Fatemeh Rajabi, Amirhossein Aghakhani, Ahmad Jayedi, Alireza Soltani Khaboushan, Masoumeh Majidi Zolbin, Mir Saeed Yekaninejad

**Affiliations:** ^1^ Pediatric Urology and Regenerative Medicine Research Center, Gene, Cell and Tissue Research Institute, Children’s Medical Center, Tehran University of Medical Sciences, Tehran, Iran; ^2^ Department of Medical Genetics, Faculty of Medical Sciences, Tarbiat Modares University, Tehran, Iran; ^3^ Department of Epidemiology and Biostatistics, School of Public Health, Tehran University of Medical Sciences, Tehran, Iran; ^4^ Social Determinants of Health Research Center, Semnan University of Medical Sciences, Semnan, Iran; ^5^ Students’ Scientific Research Center, Tehran University of Medical Sciences, Tehran, Iran

**Keywords:** breast cancer, non-coding RNAs, non-invasive tool, prognostic and diagnostic, pan-cancer analysis

## Abstract

**Background:** Breast cancer (BC) is the most common cancer in women. The incidence and morbidity of BC are expected to rise rapidly. The stage at which BC is diagnosed has a significant impact on clinical outcomes. When detected early, an overall 5-year survival rate of up to 90% is possible. Although numerous studies have been conducted to assess the prognostic and diagnostic values of non-coding RNAs (ncRNAs) in breast cancer, their overall potential remains unclear. In this field of study, there are various systematic reviews and meta-analysis studies that report volumes of data. In this study, we tried to collect all these systematic reviews and meta-analysis studies in order to re-analyze their data without any restriction to breast cancer or non-coding RNA type, to make it as comprehensive as possible.

**Methods:** Three databases, namely, PubMed, Scopus, and Web of Science (WoS), were searched to find any relevant meta-analysis studies. After thoroughly searching, the screening of titles, abstracts, and full-text and the quality of all included studies were assessed using the AMSTAR tool. All the required data including hazard ratios (HRs), sensitivity (SENS), and specificity (SPEC) were extracted for further analysis, and all analyses were carried out using Stata.

**Results:** In the prognostic part, our initial search of three databases produced 10,548 articles, of which 58 studies were included in the current study. We assessed the correlation of non-coding RNA (ncRNA) expression with different survival outcomes in breast cancer patients: overall survival (OS) (HR = 1.521), disease-free survival (DFS) (HR = 1.33), recurrence-free survival (RFS) (HR = 1.66), progression-free survival (PFS) (HR = 1.71), metastasis-free survival (MFS) (HR = 0.90), and disease-specific survival (DSS) (HR = 0.37). After eliminating low-quality studies, the results did not change significantly. In the diagnostic part, 22 articles and 30 datasets were retrieved from 8,453 articles. The quality of all studies was determined. The bivariate and random-effects models were used to assess the diagnostic value of ncRNAs. The overall area under the curve (AUC) of ncRNAs in differentiated patients is 0.88 (SENS: 80% and SPEC: 82%). There was no difference in the potential of single and combined ncRNAs in differentiated BC patients. However, the overall potential of microRNAs (miRNAs) is higher than that of long non-coding RNAs (lncRNAs). No evidence of publication bias was found in the current study. Nine miRNAs, four lncRNAs, and five gene targets showed significant OS and RFS between normal and cancer patients based on pan-cancer data analysis, demonstrating their potential prognostic value.

**Conclusion:** The present umbrella review showed that ncRNAs, including lncRNAs and miRNAs, can be used as prognostic and diagnostic biomarkers for breast cancer patients, regardless of the sample sources, ethnicity of patients, and subtype of breast cancer.

## 1 Introduction

Breast cancer (BC) is the most prevalent cancer in females, which accounted for 29% of all new cancer diagnoses among women in 2015. BC incidence and morbidity are predicted to increase fast, with an estimated 268,600 new cases per year (Li et al., 2018; Heidari et al., 2021). The high mortality rates and low 5-year survival rates of BC can be primarily attributed to the difficulty of detecting the disease early and the lack of effective therapeutic approaches and drugs. Clinical outcomes of BC are strongly influenced by the stage at which the disease is diagnosed, and an overall 5-year survival rate of up to 90% can be achieved when it is detected early, while survival drops to 20% when it has already spread to distant organs (Xie et al., 2016). Chemoresistance and distant-site metastasis, which are the leading causes of death in breast cancer, pose challenges to effective management and treatment (Chang et al., 2016). In spite of the progress in treatment options, many patients suffer from relapses of metastatic diseases, which lead to their deaths (Lei et al., 2016). As a result, a more thorough understanding of the molecular mechanisms of BC progression may lead to more effective prognostic biomarkers and therapeutic targets.

Cancer tumor size, histological subtype and grade, lymph node metastases, and lymphovascular invasion are classical clinicopathological features indicating BC’s prognosis based on careful histological analysis (Weigel and Dowsett, 2010). However, they can only be useful for a limited number of patients because of their low prognostic capacity (Zhu et al., 2020). The biopsy process is unpleasant and risky for the patients and expensive and time-consuming, and it requires a certain level of expertise from the pathologist. Considering this, alternative methodologies to find non-invasive cancer biomarkers are gaining increasing attention because they are less painful, easier to sample, and potentially more economical (Pardini et al., 2019). A biomarker is any measurable indicator that can detect malignancy or predict tumor behavior, prognosis, or response to therapy (Li et al., 2017). BC is currently diagnosed primarily by imaging, pathology, and serological markers (Liu et al., 2019a). Despite their advantages, these methods have weaknesses, including invasiveness, inconvenience, higher costs, and a high rate of false-positive results. Mammography is the gold standard for the diagnosis of BC, but it is associated with some drawbacks, such as ionizing radiation’s harmful effects and low sensitivity for early detection. Also, a needle biopsy or surgical biopsy, which is typically used to confirm a breast cancer diagnosis, is not only invasive but also not needed in most cases when the tumors are benign. In contrast to the aforementioned methods, the circulating tumor biomarker-based method is simple, convenient, and affordable for detecting BC at early stages and predicting its progression or recurrence (Xie et al., 2016; Li et al., 2018; Li et al., 2020). In clinical practice, several specific biomarkers are currently used, including the carcinoembryonic antigen (CEA), carbohydrate antigen 15-3 (CA15-3), progesterone receptor (PR), and estrogen receptor (ER). However, their sensitivity and specificity are not enough to predict BC. These findings indicated the urgent need for new sensitive, specific, and non-invasive biomarkers to diagnose BC (Jiang et al., 2020).

A large majority of the human genome can be encoded by non-coding RNAs (ncRNAs) (Zhang et al., 2015a) that regulate gene transcription and are categorized into short non-coding RNAs (20–200 nt) and long non-coding RNAs (200 nt–10 kb) (Shi et al., 2016). The use of non-coding RNAs as biomarkers for various diseases’ detection has recently been reported as one of the most promising molecular agents (Carter et al., 2017; Wang et al., 2019a; Zafari et al., 2021a). They are robust and non-invasive biomarkers that can be monitored in the bloodstream (LIU et al., 2019; Yi et al., 2020; Zafari et al., 2021b). The three most important classes of the ncRNA family are microRNA (miRNA), long non-coding RNA (lncRNA), and circular RNA (circRNA) (Chandra Gupta and Nandan Tripathi, 2017; Powrózek and Ochieng Otieno, 2022). Studies have indicated that miRNAs and lncRNAs display differential expression in different developmental stages and pathological conditions of cancers and other complications (Tomar et al., 2020; Bahramy et al., 2021). Several studies have shown that miRNAs are abnormally expressed in plasma during the onset and progression of breast cancer, suggesting that miRNAs may serve as biomarkers for early detection of breast cancer (Liu et al., 2019a). Due to the fact that miRNAs function as tumor suppressors or oncogenes, control the expression of many genes, and that they comprise around 1%–4% of the genome, they can be applied as biomarkers (Mulrane et al., 2013; Rasool et al., 2016). There is evidence that certain circulating miRNAs can be used to differentiate breast cancer from healthy controls and even benign lesions (Li et al., 2020). lncRNAs are another subset of non-coding RNAs, and their importance in normal development and tumorigenesis is being investigated. They play a key role in alternative splicing, gene regulation, histone modification, chromatin rearrangement, and gene expression regulation in BC (Soudyab et al., 2016). circRNA is another novel class of endogenous ncRNA molecules with diverse functions and frequent tissue-specific expression (Lü et al., 2017a). Researchers found that circRNAs may be markers of cell proliferation and be associated with cancer subtypes in breast cancer. Lü et al. (2017a) reported that circRNAs are differentially expressed in BC through participation in cancer-related pathways and sequestering miRNAs. In addition to acting as microRNA sponges, they interact with RNA-binding proteins to regulate gene expression. The studies showed that they contributed to the onset and development of tumors (Chu et al., 2021). Thus, ncRNAs would be proper diagnostic candidates for investigating their potential. There is growing evidence that microRNAs and lncRNAs play a critical role in cancer initiation, progression, and metastasis, making them valuable biomarkers for detecting and predicting breast cancer (Li et al., 2017; Elghoroury et al., 2018; Zhu et al., 2020).

Numerous systematic reviews and meta-analyses have been conducted on prognostic and diagnostic biomarkers for BC. However, as far as we know, the results of these systematic reviews and meta-analyses have not been synthetically assessed. Available meta-analyses and systematic reviews were analyzed and graded based on their credibility in order to provide an overview of correlations between BC survival outcomes and prognostic biomarkers and to identify robust prognostic and diagnostic biomarkers for clinical practice.

## 2 Materials and methods

### 2.1 Prognostic and diagnostic studies

#### 2.1.1 Search strategy

The current umbrella review was registered in PROSPERO and was accepted for further investigation on 04 February 2022 (PROSPERO ID number: CRD42022301393 and CRD42022300459). A comprehensive search was conducted across three different databases, namely, PubMed, Scopus, and Web of Science (WoS), on 8 January 2022. The main search keywords and MeSH terms that were applied in the diagnostic part of the article include “diagnostic/diagnosis early/diagnostic, molecular” and “miRNA/microRNA” and “lncRNA/long non-coding RNA” and “breast cancer/breast carcinoma/breast, carcinoma/breast, cancer” and “non, coding, RNA/noncoding RNA/untranslated/small non-coding RNA”. Searched keywords and MeSH terms for prognostic studies included “breast cancer,” “microrna,” “non-coding rna,” “small non-coding rna,” “prognosis,” and “prognostic value.” Language and other limitations were not applied to our search. The reference lists of the eligible studies identified in the databases and gray literature were also manually checked. The search strategy of PubMed can be found in Supplementary Tables S1, S2. Other databases’ strategies are available in Supplementary Tables S3, S4.

#### 2.1.2. Inclusion and exclusion criteria

All eligible articles that were included in the diagnostic section of our study met the following criteria: (Heidari et al., 2021): systematic review and meta-analysis studies, meta-analysis studies, and studies that conducted meta-analysis as a part of their original or review study; (Li et al., 2018) articles that analyzed the overall potential of ncRNAs as breast cancer diagnostic markers; (Xie et al., 2016) articles that included the studies that focused on the breast cancer patients with histopathological evidence that their clinical data were complete; (Chang et al., 2016) studies that investigated diagnostic potential of ncRNAs in plasma, serum, and tissue; and (Lei et al., 2016) studies that focused on any subtypes of breast cancer. Of these studies in the meta-analysis part, we included the articles that 1) had sufficient data to calculate true positives (TPs), false positives (FPs), false negatives (FNs), and true negatives (TNs) (these data were essential to measure the sensitivity and specificity) and 2) analyzed the ncRNAs’ diagnostic accuracy individually or as a panel. The following criteria were considered for inclusion in the prognostic section of our study: 1) focus on the association between ncRNAs and survival outcomes in breast cancer patients, including overall survival (OS), progression-free survival (PFS), disease-free survival (DFS), cancer-specific survival (CSS), recurrence-free survival (RFS), metastasis-free survival (MFS), disease-specific survival (DSS), and distant relapse-free survival (DRFS); 2) providing the effect size and confidence interval (CI) or appropriate data for calculating them; and 3) providing hazard ratios (HRs), odds ratios (ORs) or relative risks (RRs), and 95% CI. Studies that met the following criteria were excluded from our analysis: (Heidari et al., 2021) the control groups in the study were not clearly stated or the control group were not healthy individuals; (Li et al., 2018) the study did not involve humans; (Xie et al., 2016) data were not available in the article; and (Chang et al., 2016) originals, reviews, editorial articles, expert opinions, animal studies, conference abstracts, and *in vitro* and *ex vivo* studies. Two researchers (AB and NZ) independently assessed all these criteria, and any discrepancy was resolved by consensus.

#### 2.1.3 Quality assessment

The quality of primary diagnostic and prognostic studies was evaluated through AMSTAR version 2.0 (A Measurement Tool to Assess Systematic Reviews) (Shea et al., 2017). A useful tool to assess the methodological quality of eligible systematic reviews and meta-analysis studies included in an umbrella review was used in the current study and can be accessed through “https://amstar.ca/Amstar_Checklist.php”. It includes 16 critical items to ensure that the quality assessment is as thorough as possible. Articles were graded as high, moderate, low, or critically low based on these items.

#### 2.1.4 Data extraction

Two researchers (AB and NZ) independently evaluated all retrieved studies for suitability and extracted relevant data. The extracted data factors for diagnostic articles included first author name, date of publication, country of origin, databases, study design, number of patients, sample type, diagnostic parameters, publication bias (*p*-value), threshold effect, source of heterogeneity, biomarkers’ names, and relative risk estimates including the diagnostic odds ratio (DOR), negative likelihood ratio (NLR), positive likelihood ratio (PLR), the area under the curve (AUC), sensitivity, specificity, and other required information of the eligible systematic reviews and meta-analyses. Also, the prognostic section included the first author, search date, country, searched databases, population size, number of included studies, sample sources, biomarkers’ names, HR (OR or RR) and the corresponding 95% CI, and any related data that were provided in the subgroup analysis part. Discrepancies were also resolved by further discussion.

#### 2.1.5 Statistical analysis

Stata software (version 14.0, StataCorp, MIDAS module) was used for meta-analysis. In diagnostic studies, true-positive (TP), false-positive (FP), false-negative (FN), and true-negative (TN) values were calculated based on sensitivity, specificity, and prevalence of breast cancer. The AUC was assessed by the summary receiver operating characteristic curve (SROC). Fagan’s nomogram and likelihood ratio plots were used to determine ncRNA clinical applicability, in which a PLR >10 and an NLR <0.1 are considered high clinical applicability. The heterogeneity was analyzed by chi-squared and I^2^ tests, and *p* <0.1 or I^2^ >50% is considered to be significant. In the prognostic section of this study, the obtained data of OS and/or DFS and/or RFS and/or PFS and/or MFS and/or DSS and other factors were analyzed using Stata version 12.0 software (Stata Corporation, College Station, Texas, USA). For all of them, the HR values were pooled, and heterogeneity tests were performed. I^2^ >50% is considered to be significant heterogeneity (Egger et al., 1997). If I^2^ >50%, the random-effects model was used, and if I^2^ < 50%, the fixed-effects model was used to analyze the pooled HR values and 95% CI. When I^2^ >50%, we also performed subgroup and meta-regression analyses. Forest plots were generated as graphical representations. Deeks’ funnel plot asymmetry test was used to trace the potential publication bias among studies, and *p* <0.01 indicates significant statistical publication bias.

### 2.2 Pan-cancer analysis

We conduct further analysis to see if there is a correlation, in terms of survival analysis, between the data in online databases and the data in these systematic reviews and meta-analyses. First and foremost, it was necessary to find shared target genes of significant ncRNAs in the included studies. Web-based tools were used to find potential target genes, of which we used four in our investigation: miRNet [a web-based tool that searches the miRTarBase and TargetScan databases for predicted and experimentally validated target genes (https://www.mirnet.ca/)], miRTargetLink Human [which contains experimentally validated miRTarBase interactions and predicted targets generated with miRanda (https://www.ccb.uni-saarland.de/)], Mienturnet [computationally predicted and/or experimentally validated miRNA–target interactions from TargetScan (http://userver.bio.uniroma1.it/apps/mienturnet/)], and mirDIP [providing nearly 152 million human microRNA–target predictions, which were collected across 30 different resources (https://ophid.utoronto.ca/mirDIP/)]. Then, using a Venn diagram, we looked for common genes that these four tools targeted. We discovered seven genes: E2F3, SMAD4, SMAD7, IRAK1, BTG2, UBR5, and VEGFA. Additionally, we drew a Sankey diagram to display the network of connections between the predicted genes, miRNAs, and lncRNAs from these studies. Following that, in order to determine whether these genes are connected to breast cancer-related pathways, we carried out Gene Ontology (GO) and pathway analysis to identify the biological pathways (BPs), cellular components (CC), and molecular functions (MF) in which these genes are involved.

Finding out how these molecules (ncRNAs and mRNAs) are expressed in global databases like Pan-Cancer is crucial. These databases should also be used to re-analyze the impact of miRNAs, lncRNAs, and genes on patient survival. To assess the expression of ncRNAs and genes, we use the ENCORI database (https://starbase.sysu.edu.cn/). The Kaplan–Meier plotter (https://kmplot.com/analysis/—pan-cancer RNA-seq) was used to perform the survival analysis for both OS and relapse-free survival (RFS).

## 3 Results

### 3.1 Prognostic study

#### 3.1.1 Characteristics of included systematic reviews and meta-analysis studies

The primary search of PubMed, Scopus, and WoS resulted in the discovery of 10,548 articles in total; 3,827 duplicate articles were removed, and 6,721 articles remained for title and abstract screening, of which 129 articles underwent full-text screening. After a precise evaluation of each article’s full text, 67 articles were excluded because they did not meet the inclusion criteria. Totally, 58 systematic reviews and meta-analysis studies and 141 datasets were included in the current umbrella review. The flowchart is shown in Supplementary Figure S1. A total of 141 different associations between the survival rate of breast cancer patients and ncRNAs as prognostic biomarkers were retrieved from these included articles. These studies covered more than 50,000 patients and included 400 research articles. For further analyses, data were carefully extracted from included articles by two independent authors (NZ and AB), and any discrepancy was resolved by discussion. The main characteristics of included systematic reviews and meta-analyses are provided in Supplementary Table S5 based on included articles.

#### 3.1.2 Methodological quality assessment

All items of the AMSTAR 2.0 tool were assessed for each study. Of the 16 items, two were missing in all articles: item 2 and item 10. Therefore, after consulting with an epidemiologist, these two items were not considered while assessing the quality of the included studies. A total of 23, 11, and 24 studies were graded as moderate, low, and critically low quality, respectively. Item 3 was missing in nine studies (15%). Item 4 was missing in nine studies (15%) and determined as “partial yes” in 21 studies (36%). Items 5 and 6 were missing in 13 studies (22%). Item 7 was missing in 29 studies (50%) and determined as “partial yes” in the rest (50%). Items 9, 12, and 13 of the AMSTAR items were missing in 20 (34%) and 23 (39%) studies. Nine (15%) studies were not considered “yes” in item 14, and 12 studies were determined to be “no” in item 15. Item 16 was missing in only four studies (6.8%). The detailed results of the quality assessment are shown in Supplementary Table S6.

#### 3.1.3 Association of ncRNA expression with the survival rate of breast cancer patients

The OS and/or DFS and/or RFS and/or PFS and/or MFS and/or DSS of 205,184, 32,010, 31,410, 17,295, 9,109, and 9,010 patients in 87, 26, 20, 12, 7, and 4 datasets were analyzed, respectively, and the main results are shown in Supplementary Figure S2. The meta-analysis of these datasets showed that ncRNA expression is associated with poor OS (HR = 1.521), poor DFS (HR = 1.33), poor RFS (HR = 1.66), poor PFS (HR = 1.71), good MFS (HR = 0.90), and good DSS (HR = 0.37) (Table 1).

**TABLE 1:** Prognostic value of ncRNA expression in breast cancer.

Variable	Survival outcome	No. of datasets	No. of patients	HR (95% CI)	(*p*-value)	Model	Heterogeneity I^2^ and H^2^
ncRNAs	OS	87	205,184	1.52 (1.345–1.721)	0.00	Random effects	94.30% and 17.53
	DFS	26	32,010	1.33 (0.99–1.78)	0.00	Random effects	95.82% and 23.91
	RFS	20	31,410	1.664 (1.254–2.207)	0.00	Random effects	90.48% and 10.51
	PFS	12	17,295	1.706 (1.233–2.360)	0.00	Random effects	85.59% and 6.94
	MFS	7	9,109	0.904 (0.266–3.078)	0.00	Random effects	98.46% and 64.74
	DSS	4	9,010	0.374 (0.043–3.276)	0.00	Random effects	98.53% and 67.82
miRNAs	OS	56	60,376	1.633 (1.417–1.881)	0.00	Random effects	92.77% and 13.84
	DFS	16	11,011	1.64 (1.24–2.17)	0.00	Random effects	89.97% and 9.97
	RFS	8	7,800	1.963 (1.328–2.901)	0.00	Random effects	87.51% and 8.01
	PFS	10	7,243	1.820 (1.334–2.483)	0.00	Random effects	76.07% and 4.18
Single miRNAs	OS	50	50,653	1.641 (1.405–1.916)	0.00	Random effects	90.33% and 10.34
	DFS	15	10,561	1.635 (1.227–2.177)	0.00	Random effects	90.75% and 10.81
	RFS	8	7,800	1.963 (1.328–2.901)	0.00	Random effects	87.51% and 8.01
	PFS	9	6,512	1.778 (1.250–2.528)	0.00	Random effects	76.83% and 4.32
Combined miRNAs	OS	6	9,723	1.631 (1.057–2.516)	0.00	Random effects	91.61% and 11.92
lncRNAs	OS	27	23,955	1.439 (1.193–1.735)	0.00	Random effects	87.36% and 7.91
	DFS	8	12,761	0.808 (0.314–2.082)	0.00	Random effects	98.75% and 79.92
	RFS	10	13,558	1.369 (0.704–2.664)	0.00	Random effects	95.83% and 23.98
	MFS	4	7,463	0.470 (0.047–4.730)	0.00	Random effects	99.25% and 133.13
Single lncRNAs	OS	23	12,325	1.445 (1.185–1.764)	0.00	Random effects	83.15% and 5.94
	DFS	6	7,423	0.551 (0.149–2.038)	0.00	Random effects	99.13% and 115.45
	RFS	9	11,366	1.231 (0.565–2.681)	0.00	Random effects	97.05% and 33.91
	MFS	4	7,463	0.470 (0.047–4.730)	0.00	Random effects	99.25% and 133.13
Combined RNAs	OS	4	11,630	1.402 (0.782–2.513)	0.00	Random effects	96.35% and 27.37

After putting aside critically low-quality articles, the meta-analysis of these datasets showed that ncRNA expression is associated with poor OS (HR = 1.52), poor DFS (HR = 1.34), poor RFS (HR = 1.54), poor PFS (HR = 1.71), poor MFS (HR = 1.18), and good DSS (HR = 0.73) (Supplementary Figure S3).

We also analyzed the association of miRNAs (Supplementary Figure S4) and lncRNAs (Supplementary Figure S5) separately and as a single and combined panel. miRNA expression is associated with poor OS (HR = 1.63) in 60,376 patients and 56 datasets, with poor DFS (HR = 1.64) in 11,011 patients and 16 datasets, with poor RFS (HR = 1.96) in 7,800 patients and eight datasets, and with poor PFS (HR = 1.82) in 7,243 patients and 10 datasets. Particularly, single miRNAs are associated with poor OS (HR = 1.64) in 50,653 patients and 50 datasets, with poor DFS (HR = 1.63) in 10,561 patients and 15 datasets, with poor RFS (HR = 1.96) in 7,800 patients and eight datasets, and with poor PFS (HR = 1.78) in 6,512 patients and nine datasets. Moreover, combined miRNAs are associated with poor OS (HR = 1.63) in 9,723 patients and six datasets (Table 1). According to the results, single miRNAs are more robust biomarkers in association with OS than combined miRNAs.

lncRNA expression is associated with poor OS (HR = 1.44) in 23,955 patients and 27 datasets, with good DFS (HR = 0.81) in 12,761 patients and eight datasets, with poor RFS (HR = 1.37) in 13,558 patients and 10 datasets, and with good MFS (HR = 0.47) in 7,463 patients and four datasets. Single lncRNAs are associated with poor OS (HR = 1.45) in 12,325 patients and 23 datasets, with good DFS (HR = 0.55) in 7,423 patients and six datasets, with poor RFS (HR = 1.23) in 11,366 patients and nine datasets, and with good MFS (HR = 0.47) in 7,463 patients and four datasets. Moreover, combined lncRNAs are associated with poor OS (HR = 1.40) in 11,630 patients and four datasets (Table 1). Hence, miRNAs have a stronger association with the aforementioned factors than lncRNAs and would be better biomarkers based on the aforementioned results.

#### 3.1.4 Association of ncRNA expression with the survival rate based on ethnicity, sample sources, and subtypes

When grouped based on ethnicity, the pooled HRs of the Asian and non-Asian populations in OS were 3.08 and 2.11, respectively, which show that ncRNAs are indicators of poor OS prognoses regardless of ethnicity. We also recognized that ncRNA expression in both blood (including plasma and serum) and tissues is associated with poor OS prognoses as well (HR = 3.50 and 1.77). However, their expression in triple-negative breast cancer is not significantly associated with poor OS prognoses. When we considered a mixed subtype of breast cancer, it resulted in a significant association with an HR of 2.19 (Table 2) (Supplementary Figure S7).

**TABLE 2:** Association of ncRNA expression with the survival rate based on different variables.

Variable	Survival outcome	No. of datasets	No. of patients	HR (95% CI)	(*p*-value)	Model	Heterogeneity I^2^ and H^2^
Asian	OS	7	2,621	3.08 (2.04–4.66)	0.01	Random effects	71.92% and 3.56
Non-Asian	OS	8	2,896	2.11 (1.45–3.09)	0.00	Random effects	85.44% and 6.87
Blood	OS	4	1,107	3.50 (1.91–6.41)	0.00	Random effects	73.37% and 3.76
Tissue	OS	4	2,773	1.77 (1.25–2.51)	0.00	Random effects	85.62% and 6.95
TNBC	OS	4	459	1.97 (1.58–2.46)	0.75	Random effects	0.00% and 1.00
Mixed-typed BC	OS	4	13,302	2.19 (1.33–3.62)	0.00	Random effects	75.34% and 4.06

#### 3.1.5 Association of most frequently studied ncRNA expression with the survival rate

Among the most frequent ncRNAs, miR-21 and miR-210 expression are significantly associated with poor OS, and MALAT1 expression is associated with both OS and DFS; however, HOTAIR expression had no significant association with poor OS (Table 3) (Supplementary Figure S8).

**TABLE 3:** Association of most frequently studied ncRNA expression with the survival rate.

Variable	Survival outcomes	No. of datasets	No. of patients	HR (95% CI)	(p-value)	Model	Heterogeneity I^2^ and H^2^
miR-21	OS	9	7,255	1.93 (1.62–2.30)	0.02	Random effects	55.65% and 2.25
	DFS	5	4,682	1.45 (1.27–1.64)	0.33	Random effects	10.07% and 1.11
miR-210	OS	8	5,822	2.07 (1.34–3.20)	0.00	Random effects	87.59% and 8.06
	DFS	4	2,586	3.20 (2.66–3.85)	0.61	Random effects	0.00% and 1.00
MALAT1	OS	4	3,683	1.66 (1.07–2.56)	0.00	Random effects	88.34% and 8.57
	RFS	6	9,738	0.87 (0.21–3.63)	0.00	Random effects	98.79% and 82.72
HOTAIR	OS	3	996	1.29 (1.03–1.62)	0.99	Random effects	0.00% and 1.00

#### 3.1.6 Meta-regression analysis and publication bias

In order to find the probable sources of heterogeneity, we did a meta-regression analysis. For OS, we tested the sources of heterogeneity based on miRNAs vs. lncRNAs, single vs. combined miRNAs, single vs. combined lncRNAs, the number of cases, and the AMSTAR score. When we tested one variable at a time, the AMSTAR score was a source of heterogeneity. However, when we tested these variables together, the types of ncRNAs and whether they were single or combined miRNAs were found to be sources of heterogeneity (Supplementary Table S7). In DFS, no source of heterogeneity was found when testing each variable separately, but when grouped, the type of ncRNA could be a source of heterogeneity (Supplementary Table S8). In RFS, AMSTAR was found as a source of heterogeneity in a separate analysis, but in the group, there was no source of heterogeneity (Supplementary Table S9). As shown in the funnel plot, no evidence of publication bias was found (Supplementary Figure S6).

### 3.2 Diagnostic study

#### 3.2.1 Selection of studies

A total of 8,453 articles were initially obtained from PubMed (*n* = 4,080), Scopus (*n* = 683), and WoS (*n* = 2,857) databases (Supplementary Figure S7). Among these articles, 1,808 duplicate articles were removed, and 6,645 articles remained. Based on the titles and abstracts, 50 articles were left for full-text evaluation. Finally, 28 articles were excluded, and 22 meta-analysis articles (Liu et al., 2014a; Wang et al., 2014c; Xin et al., 2014; Shen et al., 2015b; Cui et al., 2015; Gao et al., 2016a; Gao et al., 2016b; Hou et al., 2016; Li et al., 2016; Xie et al., 2016; Imani et al., 2017; Tang et al., 2017; Xin et al., 2017; Cai et al., 2018; Yu et al., 2018; Wang et al., 2019c; Jiang et al., 2020; Zhao et al., 2020; Liu et al., 2021a; Liu et al., 2021b; Zhang et al., 2021) were extracted for further analysis. Among which, 30 datasets were obtained, and subsequently, two miRNAs were selected as the most frequent ncRNAs that have been assessed as a non-invasive biomarker in systematic reviews and meta-analysis studies.

#### 3.2.2 Characteristics of included studies

The characteristics of included articles are shown in Supplementary Table S10 (Liu et al., 2014a; Wang et al., 2014c; Xin et al., 2014; Shen et al., 2015b; Cui et al., 2015; Gao et al., 2016a; Gao et al., 2016b; Hou et al., 2016; Li et al., 2016; Imani et al., 2017; Xin et al., 2017; Cai et al., 2018; Yu et al., 2018; Wang et al., 2019c; Zhao et al., 2020; Liu et al., 2021b; Zhang et al., 2021). The total number of patients and healthy individuals in these 30 databases was 28,765 and 17,254, respectively. The publication years of these studies ranged from 2014 to 2020. The quality of the included studies was between high and moderate to high. Among the 22 articles, three studies were carried out on lncRNAs, 17 studies were evaluated for miRNAs, two articles studied both of them, and one article focused on circRNAs. The ncRNAs MALAT1 (three studies), let-7 family (one study), miR-155 (four studies), miR-195 (one study), miR-21 (four studies), H19 (two studies), HOTAIR (two studies), miR-34a (one study), precursor miR-203a (one study), and miR-203a-3p (one study) were assessed in plasma, serum, or tissue to find their potential as (a) non-invasive diagnostic biomarker(s). All articles were available in PubMed except study no. 5, “The roles of ncRNAs in the diagnosis, prognosis, and clinicopathological features of breast cancer: a systematic review and meta-analysis”. In this study, Tang S et al. studied miR-21, but it was excluded from our study due to a lack of complete data for analysis. As systematic reviews studied all stages, our study was not limited to a specific subtype, stage, or grade.

#### 3.2.3 Quality assessment methodology using AMSTAR 2.0

The 16-item AMSTAR 2.0 tool was used to assess the methodological quality of the 22 included meta-analyses. Items 2 and 10 were not met by any of the 22 included studies; hence, they were removed. The results showed that the qualities of all the included studies were considered moderate to low except for one meta-analysis that had critically low quality because its main focus was not meta-analysis. All included studies had more than one critical flaw [usually in items 4 (9/22: partially yes and 1/22: no) and 7 (11/22: partially yes and 11/22: no)]. The detailed results and rating criteria are shown in Supplementary Table S11.

#### 3.2.4 Overall diagnostic value of ncRNAs in breast cancer

**FIGURE 1:**
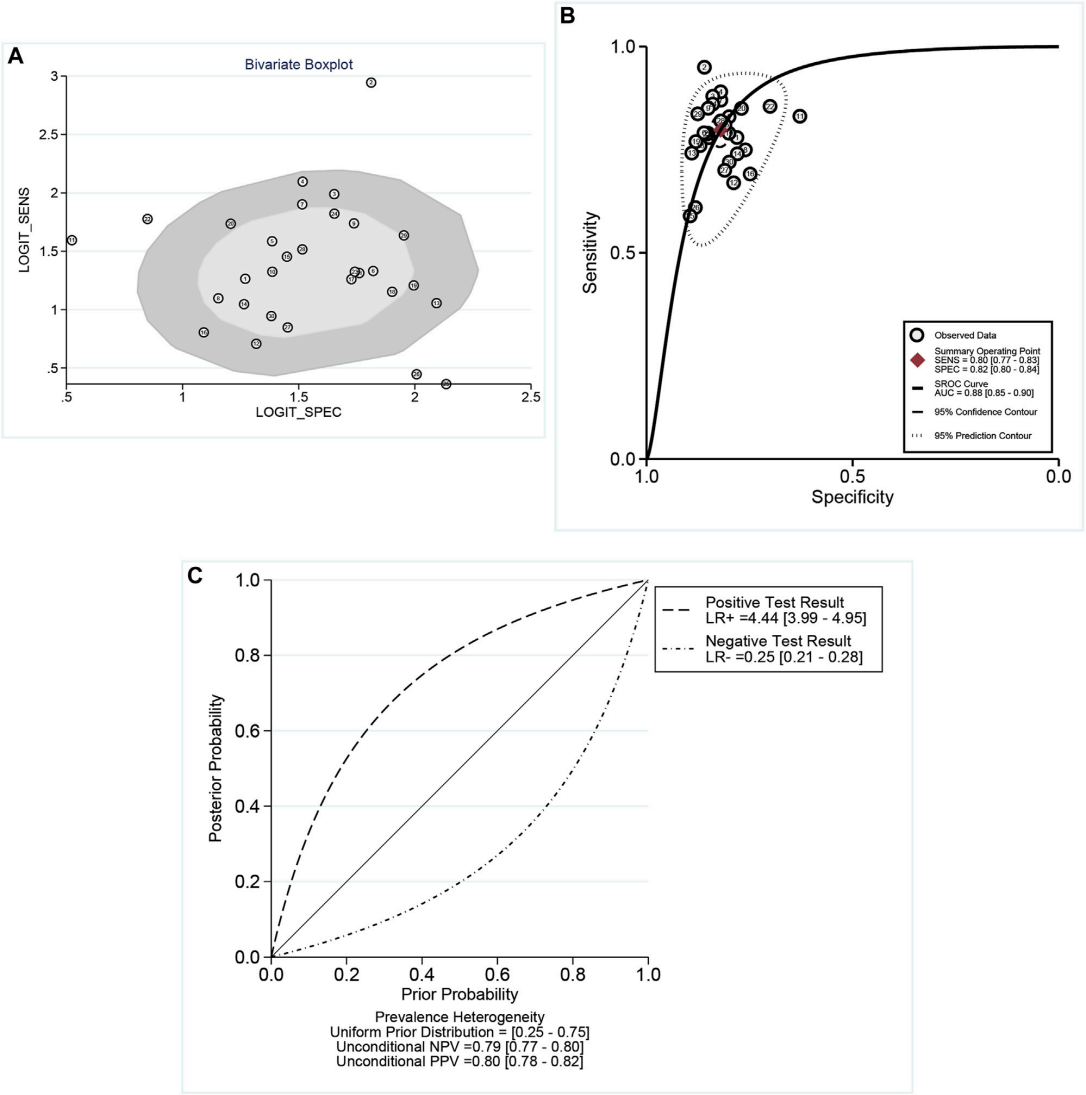
**(A)** Bivariate boxplot indicated the dispersion of datasets as an indicator of heterogeneity. **(B)** Summary receiver operator characteristic (SROC) curves. AUC, area under the curve; SENS, sensitivity; and SPEC, specificity. **(C)** Conditional plot: the overall results for NPV, PPV, LR^+^, and LR^−^. According to the bivariate boxplot, significant heterogeneity was observed among studies. I^2^ values of heterogeneity analysis resulted in 97.33 for sensitivity and 88.64 for specificity, which showed significant heterogeneity. The SROC curve showed that the values of sensitivity and specificity were 0.80 and 0.82, respectively.

**FIGURE 2:**
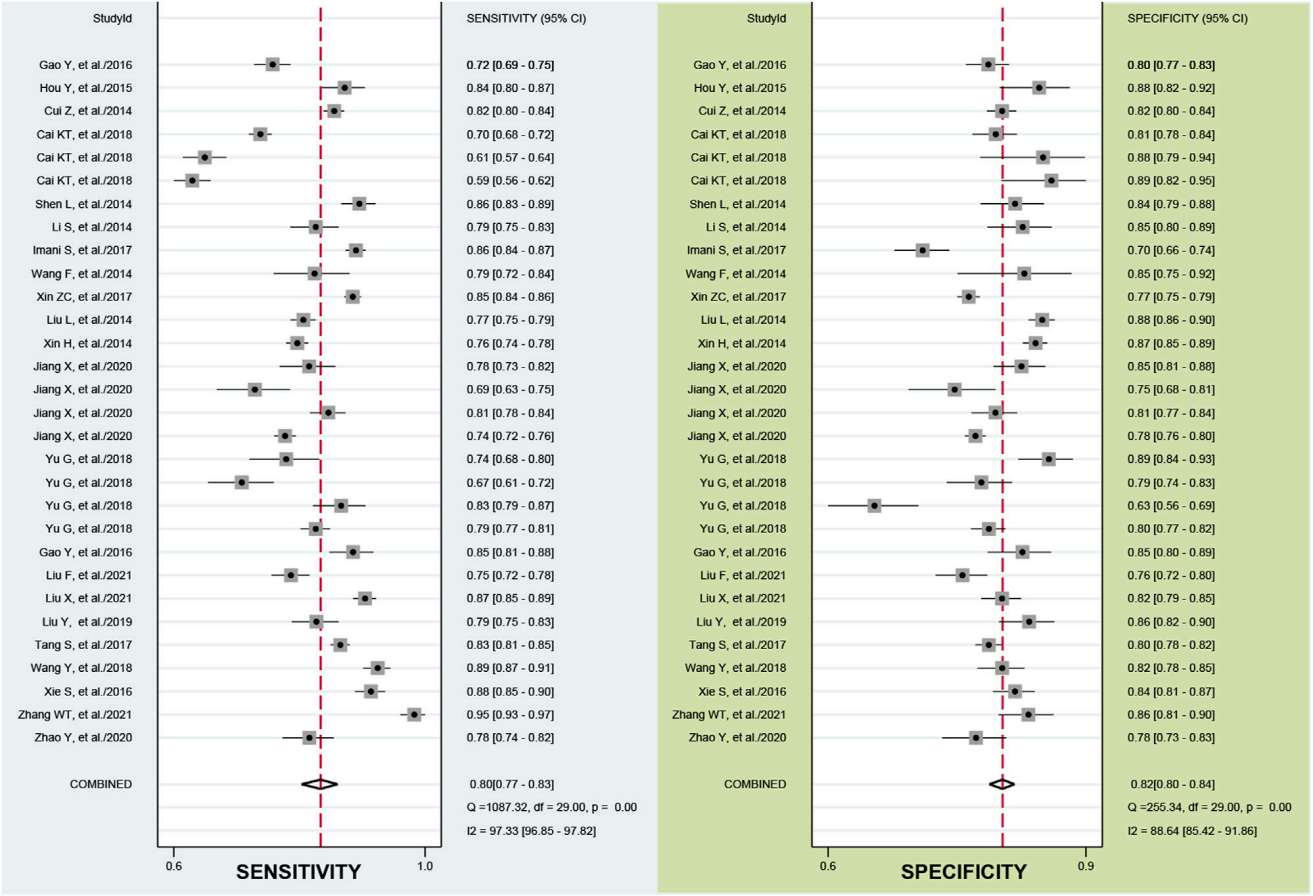
Forest plots of the sensitivity and specificity of the ncRNA assays. The overall results for negative predictive value (NPV), positive predictive value (PPV), PLR^+^, PLR^−^, sensitivity, and specificity were 0.79, 0.80, 4.44, 0.25, 0.80, and 0.82, respectively.

According to the bivariate boxplot (Figure 1A), significant heterogeneity was observed among studies, so the random-effects model was used in our meta-analysis. Forest plots of the sensitivity and specificity for ncRNAs in diagnosing BC are shown in Figure 2. The overall results for negative predictive value (NPV), positive predictive value (PPV), PLR^+^, PLR^−^, sensitivity, and specificity were 0.79 [95% CI: 0.77–0.80], 0.80 [95% CI: 0.78–0.82], 4.44 [95% CI: 3.99–4.95], 0.25 [95% CI: 0.21–0.28], 0.80 [95% CI: 0.77–0.83], and 0.82 [95% CI: 0.80–0.84], respectively. I^2^ values of the heterogeneity analysis results in 97.33 [95% CI: 96.85–97.82] for sensitivity and 88.64 [95% CI: 85.42–91.86] for specificity, indicating significant heterogeneity (Figure 1B). The SROC curve is shown in Figure 1C, where the AUC value, sensitivity, and specificity were 0.88 [95% CI: 0.85–0.90], 0.80, and 0.82, respectively, suggesting an approximately overall high accuracy for the diagnostic test.

A likelihood ratio plot was also used to assess the clinical applicability of ncRNAs (Supplementary Figure S8). The PLR and NLR were measured as indicators of diagnostic accuracy, and they were 4 and 0.25, respectively. Additionally, with a 25% pre-test probability, the positive post-test probability rises up to 60% with a PLR of 4, and the negative post-test probability drops up to 8% with an NLR of 0.25 (Supplementary Figure S8a). Based on the scatter matrix plot (Supplementary Figure S8b), the summary point with a 95% confidence interval was located in the lower right quadrant.

#### 3.2.5 Diagnostic value of ncRNAs as a panel or single biomarkers for BC detection

According to our findings, the ability of ncRNAs to distinguish BC patients from the general population is unaffected by their use in combination or as single biomarkers. Both panels and single biomarkers offer promising results (Table 4 and Supplementary Figures S9A, B).

**TABLE 4:** Diagnostic value of ncRNAs as a panel or single biomarkers.

Parameter	Sensitivity	Specificity	PLR	NLR	DOR	Threshold effect*
Single ncRNAs	0.80 [0.76, 0.84]	0.82 [0.80, 0.85]	4.5 [3.9, 5.2]	0.24 [0.20, 0.29]	19 [14, 25]	0.00
Combined ncRNAs	0.82 [0.77, 0.87]	0.82 [0.79, 0.85]	4.6 [3.9, 5.4]	0.21 [0.16, 0.28]	22 [14, 32]	0.06

* Proportion of heterogeneity likely due to the threshold effect.

#### 3.2.6 Diagnostic value of ncRNAs in different sample sources and ethnicities

First, the diagnostic value of ncRNAs was evaluated in different sample sources including plasma, serum, and tissue (Table 5, Supplementary Figures S10A–C). The result demonstrated that the use of serum showed the most promising accuracy, with a sensitivity of 0.81, a specificity of 0.82, a DOR of 19, a PLR of 4.5, and an NLR of 0.23. Additionally, the diagnostic value of ncRNAs is analyzed in Asian and Caucasian ethnicities, and the results were almost the same in these two ethnicities (Table 5, Supplementary Figures S10D, E).

**TABLE 5:** Diagnostic value of ncRNAs in different sample sources and ethnicities.

Analysis	Sensitivity (95% CI)	Specificity (95% CI)	PLR (95% CI)	NLR (95% CI)	DOR (95% CI)
Sample types					
Plasma based	0.80[0.76–0.83]	0.80 [0.73–0.86]	4.1 [3.0–5.5]	0.25 [0.22–0.29]	16 [12–22]
Serum based	0.81 [0.75, 0.86]	0.82 [0.76, 0.86]	4.5 [3.2, 6.2]	0.23 [0.16, 0.32]	19 [10, 37]
Tissue based	0.79 [0.65, 0.88]	0.67 [0.61, 0.72]	2.4 [2.0, 2.9]	0.32 [0.19, 0.54]	8 [4, 15]
Ethnicity					
Asian	0.82 [0.76, 0.87]	0.80 [0.76, 0.84]	4.2 [3.3, 5.3]	0.23 [0.17, 0.31]	18 [11, 30]
Caucasian	0.80 [0.75, 0.84]	0.81 [0.75, 0.86]	4.2 [3.2, 5.7]	0.25 [0.20, 0.31]	17 [11, 26]

#### 3.2.7 Meta-regression and subgroup analysis

Meta-regression was conducted to uncover further reasons for heterogeneity. The effect of sample size on AMSTARSco_2, lncRNAs vs. miRNAs, single miRNAs vs. combined miRNAs, and single lncRNAs vs. combined lncRNAs in each study was assessed and shown in Supplementary Figure S11. The results revealed that all parameters except sample size are sources of heterogeneity. The results of the meta-regression analysis are shown in Supplementary Table S12.

Considering that the meta-regression analysis showed that some parameters influence sensitivity and specificity significantly, therefore, we conducted subgroup analyses according to the study design in the assessment of miRNAs (single vs. combined miRNAs), ncRNAs (single vs. combined ncRNAs), and miRNAs vs. lncRNAs (Supplementary Table S12). The diagnostic value of miRNAs with 0.81 sensitivity and 0.84 specificity is higher than that of lncRNAs. Combined miRNAs with a sensitivity of 0.85 and a specificity of 0.84 are more accurate than single miRNAs. The results showed that the combined lncRNAs had more accuracy than single lncRNAs with 0.77 sensitivity; however, their specificity was the same (0.79).

#### 3.2.8 miR-21 and miR-155 as the most frequent miRNAs in studies

We selected two of the most frequent microRNAs, miR-21 (Shen et al., 2015b; Gao et al., 2016a; Gao et al., 2016b; Li et al., 2016) and miR-155 (Wang et al., 2014c; Hou et al., 2016; Liu et al., 2021b), among studies and analyzed their overall diagnostic value. MiR-21 can diagnose breast cancer with pooled sensitivity, specificity, PLR, NLR, and DOR of 0.81, 0.83, 4.9, 0.23, and 21, respectively. MiR-155 had the potential to diagnose breast cancer with pooled sensitivity, specificity, PLR, NLR, and DOR of 0.85, 0.84, 5.3, 0.17, and 31, respectively. Chi-squared and I^2^ tests were used to measure heterogeneity between miRNA’s estimated sensitivity and specificity in studies. I^2^ >50% and *p*-value <0.05 were considered as significant heterogeneity. As it was shown in Supplementary Figure S12A, the I^2^ value of the sensitivity and specificity of miR-21 is 95 and 68, which shows heterogeneity. Although the I^2^ value of the sensitivity of miR-155 is relatively high (I^2^ = 82.6), the I^2^ value of specificity is low (I^2^ = 16). It appears that miR-155 pooled specificity does not affect heterogeneity (Supplementary Figure S12B).

#### 3.2.9 Influence analysis and publication bias

**FIGURE 3:**
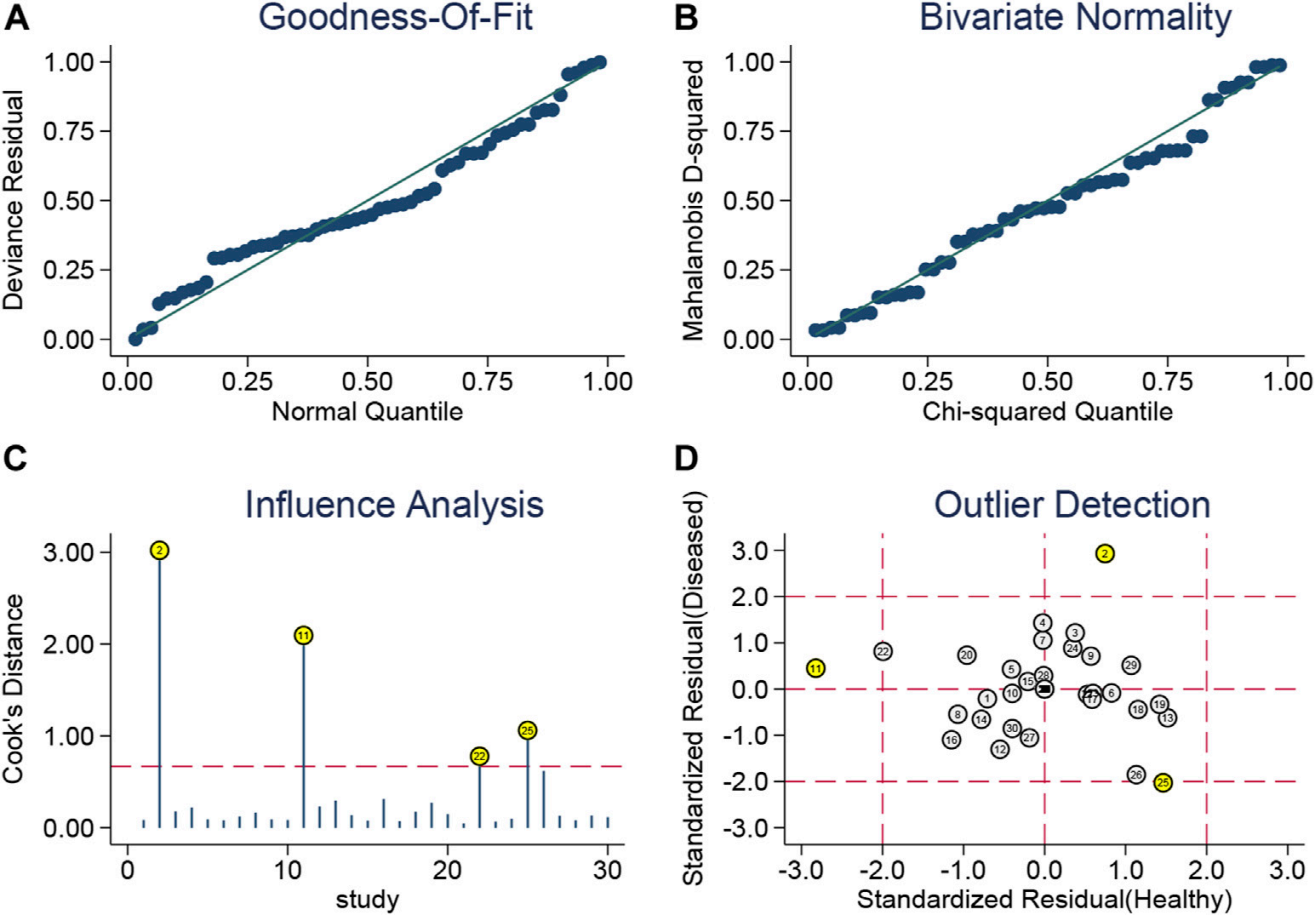
Golf diagram of **(A)** goodness-of-fit, **(B)** bivariate normality, **(C)** influence analysis, and **(D)** outlier detection. The bivariate random-effects model’s suitability is confirmed by plotting the goodness-of-fit and bivariate normality. Influence analysis revealed that datasets numbered 2, 11, 22, and 25 that belong to the let-7 family, MALAT1, miR-34a, and precursor miR-203a had Cook’s distances greater than 0.75 and were the most influential. Datasets numbered 2, 11, and 25 (related to the let-7 family, MALAT1, and precursor miR-203a, respectively) were detected as outliers, potentially accounting for some of the heterogeneity that we observed.

In this study, we confirmed the bivariate random-effects model’s suitability by plotting the goodness-of-fit and bivariate normality (Figures 3A, B). Influence analysis revealed that datasets numbered 2, 11, 22, and 25 belonging to the let-7 family, MALAT1, miR-34a, and precursor miR-203a, respectively, had Cook’s distances greater than 0.75 and were the most influential (Figure 3C). Datasets numbered 2, 11, and 25 (related to the let-7 family, MALAT1, and precursor miR-203a, respectively) were detected as outliers, potentially accounting for some of the heterogeneity that we observed (Figure 3D). To evaluate the publication bias, Deeks’ funnel plot was plotted (Supplementary Figure S13), and it showed no evidence of publication bias (*p* = 0.56) in the diagnostic analysis of ncRNAs in BC.

### 3.3 Pan-cancer analysis

#### 3.3.1 GO and pathway enrichment analysis

**FIGURE 4:**
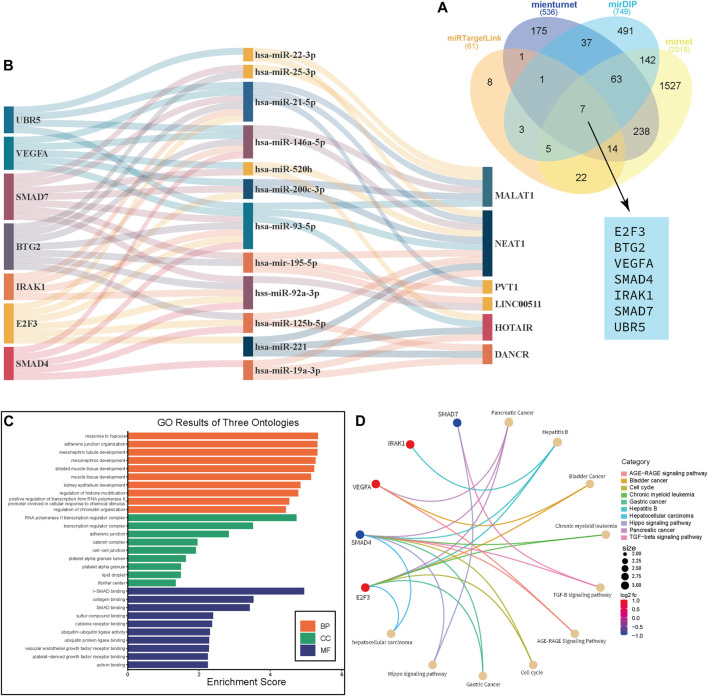
Gene Ontology and pathway analysis. Venn diagram shows the common genes that are targeted by miRNAs in this study. **(A)** Sankey diagram that shows the network between mRNA-miRNA-lncRNA. **(B)** GO enrichment analysis reveals BB, CC, and MF for target genes of lncRNAs. **(C)** Circos plot depicts the Q28 pathways in which these genes are involved **(D)**.

The target genes of the ncRNAs of interest were predicted using miRNet, miRTargetLink Human, Mienturnet, and mirDIP (Supplementary Table S13). As shown in Figure 4A, seven genes, namely, E2F3, SMAD4, SMAD7, IRAK1, BTG2, UBR5, and VEGFA, were found to be the common target of these ncRNAs. Of them, the association of UBR5 and SMAD4 with breast cancer prognosis was evaluated in previous studies (Liu et al., 2014b; Yang et al., 2020). Then, we performed a network analysis and displayed the results as a Sankey diagram to see how these genes are related to the ncRNAs under investigation (Figure 4B). To gain a deeper understanding of the role of these target genes in breast cancer, we performed GO enrichment analysis. The GO analysis showed that Biological Pathway (BP) terms were mainly enriched in the response to hypoxia, adherens junction organization, muscle tissue development, and regulation of histone modification. The CC terms are mainly involved in the RNA polymerase II transcription regulator complex, adherens junction, and cell–cell junction. The MF group was mainly enriched in SMAD binding and cytokine receptor binding (Figure 4C; Supplementary Table S14). Pathway analysis was performed to determine the pathways through which target genes were involved, and the results revealed that SMAD4, SDMAD7, and VEGFA through the hippo signaling pathway, the TGF-beta signaling pathway, and the cell cycle contribute to breast cancer, respectively (Figure 4D; Supplementary Table S14).

#### 3.3.2 Pan-cancer RNA-seq survival analysis

**FIGURE 5:**
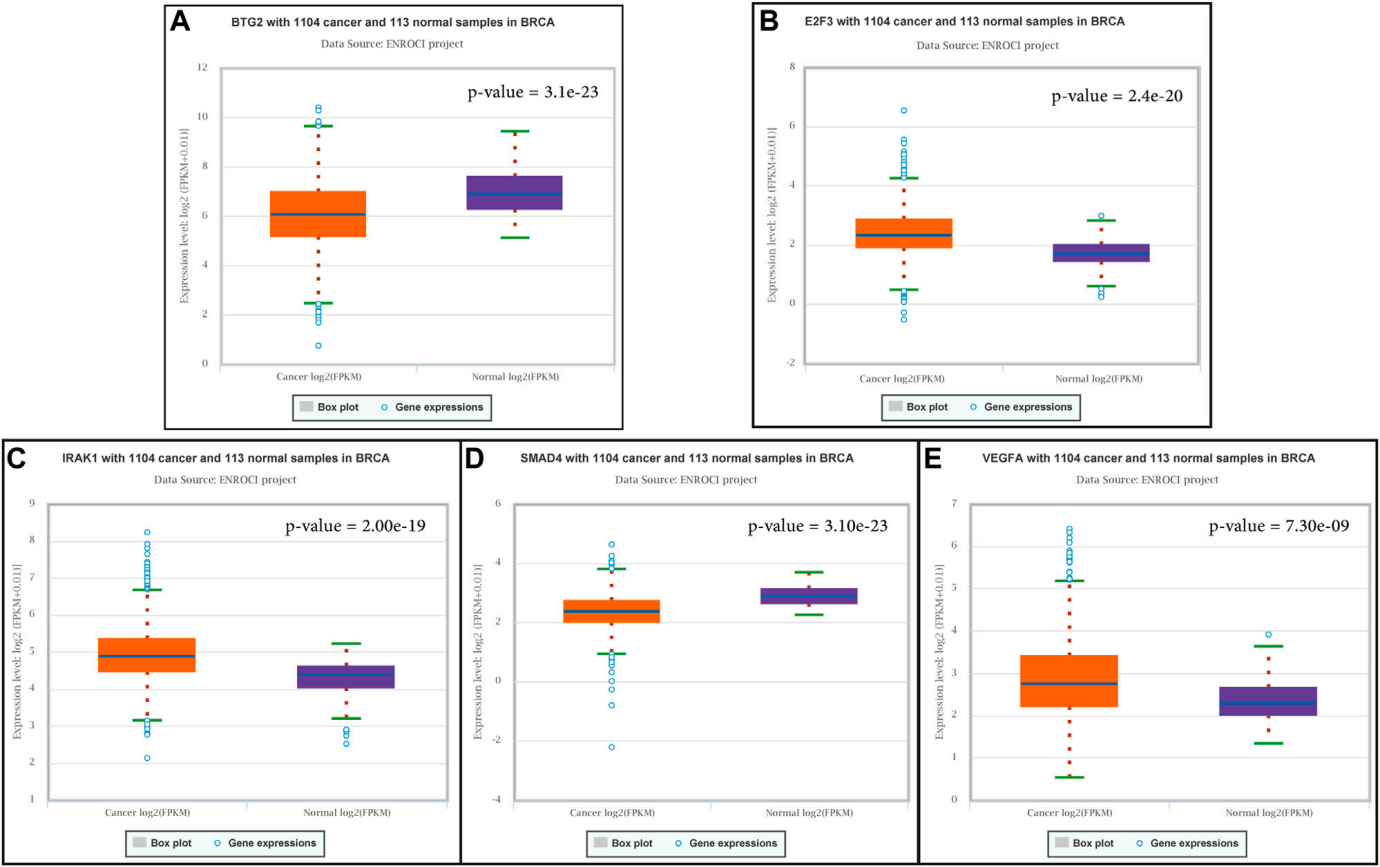
Expression of target genes of studied miRNAs in pan-cancer data.

The association of significantly differentially expressed lncRNAs, miRNAs, and their target genes with OS and RFS was evaluated. To begin, we explored how these molecules were expressed in the Pan-Cancer database [ENCORI database (https://starbase.sysu.edu.cn/panCancer.php)]. The results showed that five lncRNAs including HOTAIR, DANCR, PVT1, LINC00511, and NEAT1 and 16 miRNAs and five of their targets—VEGFA, BTG2, E2F3, IRAK1, and SMAD4—have significantly different expression patterns in normal individuals compared to those with breast cancer (Figure 5; Supplementary Figures S14, S15; Supplementary Table S15).

Additionally, we analyzed the prognostic value of ncRNAs and their targets across pan-cancer RNA-seq data. OS and RFS were evaluated on pan-cancer RNA-seq data by comparing the high and low expression of those ncRNAs and their targets that were significantly expressed in normal and cancer patients.

**FIGURE 6:**
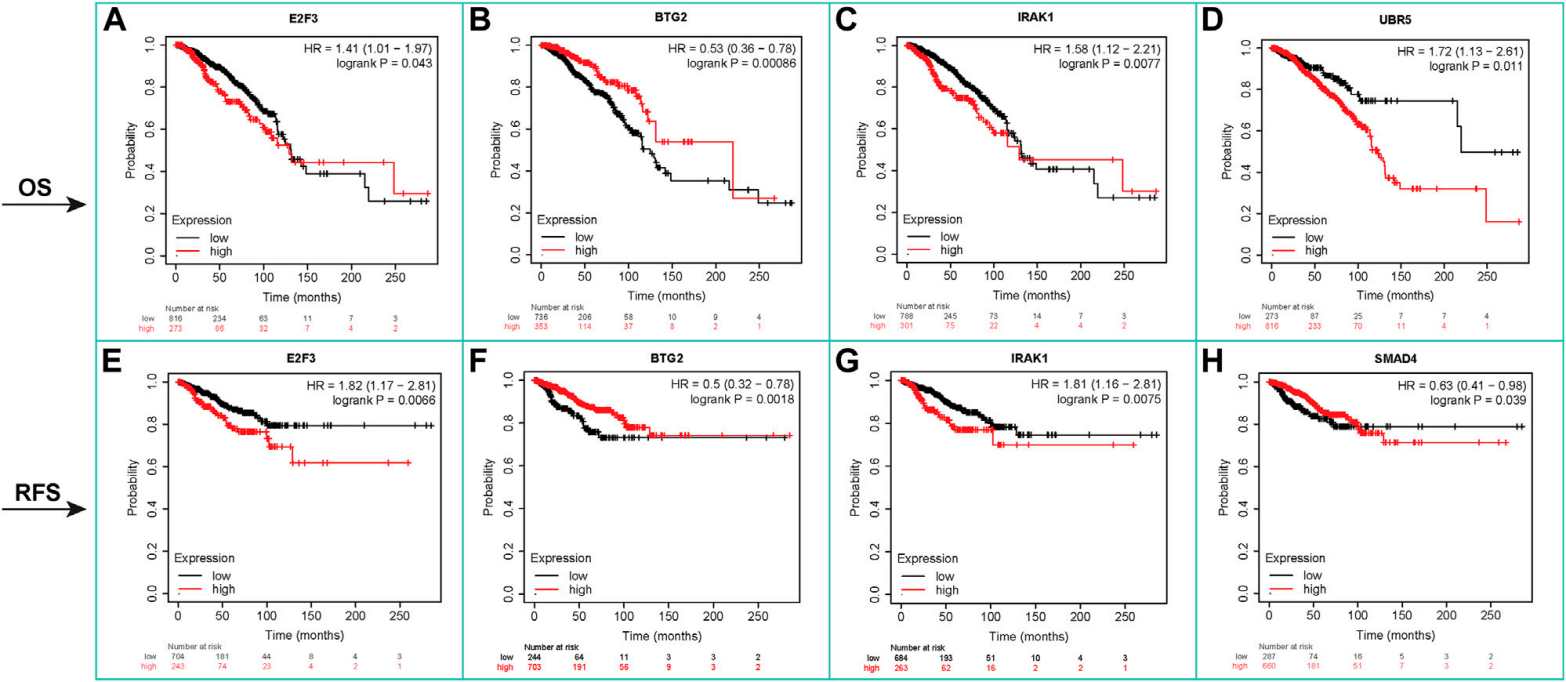
Kaplan–Meier survival analysis, OS, and RFS for mRNAs. It showed that increased expression of E2F3 (HR = 1.41), IRAK1 (HR = 1.58), and UBR5 (HR = 1.72) indicated poor OS prognosis, while decreased expression of BTG2 (HR = 0.53) is an indicator of poor OS. RFS analysis revealed that higher expression levels of E2F3 (HR = 1.82) and IRAK1 (HR = 1.81) are associated with poor RFS prognosis. Lower expression levels of BTG2 (HR = 0.50) and SMAD4 (HR = 0.63) are associated with poor RFS prognosis in patients.

Based on OS analysis data, high levels of hsa-miR-19a (HR = 1.54), hsa-miR-21 (HR = 1.63), hsa-miR-22 (HR = 1.95), hsa-miR-203a (HR = 1.44), hsa-miR-484 (HR = 1.68), hsa-miR-489 (HR = 1.81), hsa-miR-520h (HR = 2.1), and hsa-miR-4443 (HR = 1.71) are closely linked to poor OS. Meanwhile, low expression of hsa-miR-146a (HR = 0.71) is related to the poor OS prognosis of breast cancer patients (Supplementary Figure S16; Supplementary Table S16). Patients with high levels of lncRNAs, including HOTAIR (HR = 1.62), DANCR (HR = 1.41), and LINC00511 (HR = 1.51), have poor OS prognosis, whereas patients with high levels of NEAT1 (HR = 0.65) have a good prognosis. Also, patients with high levels of HOTAIR (HR = 1.62) and LINC00511 (HR = 1.6) and low levels of NEAT1 (HR = 0.64) have poor RFS prognostic indicators, respectively (Supplementary Figure S17; Supplementary Table S16). Kaplan–Meier analysis showed that increased expression of E2F3 (HR = 1.41), IRAK1 (HR = 1.58), and UBR5 (HR = 1.72) indicated poor OS prognosis, while decreased expression of BTG2 (HR = 0.53) is an indicator of poor OS. Furthermore, RFS analysis revealed that higher expression levels of E2F3 (HR = 1.82) and IRAK1 (HR = 1.81) are associated with poor RFS prognosis, whereas lower expression levels of BTG2 (HR = 0.50) and SMAD4 (HR = 0.63) are associated with poor RFS prognosis in patients (Figure 6; Supplementary Table S16).

## 4 Discussion

In recent decades, the number of women diagnosed with breast cancer has steadily increased, posing a very severe threat to their lives (Mei et al., 2020). Hence, it shows the importance of diagnostic and prognostic biomarkers to improve BC survival. Mammography is a common method for detecting breast cancer. Women younger than 40 years of age should not undergo this approach because their breast tissue is denser. Mammography has a variable rate of false positives ranging from 12% to 65%, which is responsible for the over-diagnosis of 31% of all breast cancer cases (Nassar et al., 2017). Other imaging techniques such as MRI, ultrasound, CT, and PET are also accompanied by some limitations such as low sensitivity and specificity and being expensive (Alyami, 2021; Su et al., 2021; Jin et al., 2021; Darbeheshti et al., 2022; Yousefi et al., 2022). As a result, it is deemed necessary to introduce new biomarkers that can overcome these limitations. Previous studies showed that ncRNAs are dysregulated in breast cancer (Alyami, 2021; Su et al., 2021; Jin et al., 2021). Recently, circulating RNAs have gained wide popularity as a result of their high conservation, high stability, high expression, and high specificity (Liu et al., 2021a). Although ncRNAs such as lncRNAs and miRNAs form only 1% of ncRNAs, they play essential roles in different cellular stages and tumor development. Most of the studies were carried out on miRNAs; hence, our knowledge about miRNAs is greater than that of lncRNAs in human breast cancer (Klinge, 2018). In this umbrella review, we gathered existing meta-analyses to summarize evidence related to the potential prognostic and diagnostic role of ncRNAs as biomarkers in BC.

Aiming to understand the role of ncRNAs in BC prognosis, OS and DFS of patients with different ncRNA (assessed together and/or individually) expression levels were evaluated. After systematically assessing the methodological quality and robustness of the pooled meta-analysis of 58 studies covering 205,184 participants, we found that ncRNAs are associated with poor OS with an HR of 1.52 (95% CI: 1.345–1.721) (*p* = 0.00). For more details, the expression of miRNAs was assessed in 60,376 patients, and results showed that the HR of the survival rate in BC patients is 1.633 (CI: 1.417–1.881). The analysis showed that using miRNAs as single or pooled biomarkers approximately indicated the same poor OS. Also, miRNAs discriminate OS better than lncRNAs in BC. In our study, blood-associated ncRNA expression and the survival rate showed stronger evidence than tissue ncRNAs. Among all the meta-analysis studies, miRNA-21, miR-210, MALAT1, and HOTAIR were found as the most frequently studied ncRNAs that are associated with poor OS in BC, except for HOTAIR (*p* = 0.99). The upregulation of miRNA-21, miR-210, and MALAT1 showed predictive poor prognosis in patients with BC.

In particular, miRNA-21 and miR-210 were found in various cancers such as head and neck cancer, non-small cell lung cancer, breast cancer, colorectal cancer, tongue squamous cell carcinomas, squamous cell lung carcinoma, and hepatocellular carcinoma, which were associated with poor prognosis (Camps et al., 2008; Li et al., 2009; Gee et al., 2010; Gao et al., 2011; Toiyama et al., 2013; Eilertsen et al., 2014; Huang et al., 2015; Wang et al., 2017b). According to the study by Wang and colleagues, miR-21 may not be a suitable diagnostic biomarker, but it has a prognostic value in cancer patients (Wang et al., 2014d). MiR-21 showed a significant association with OS (HR: 1.93 (95% CI: 1.62–2.30), *p* = 0.02); however, it did not have significant association with DFS (HR: 1.45 (95% CI: 1.27–1.64), *p* = 0.33). Apparently, the level of miR-21 has a negative correlation with the survival rate in breast cancer patients (Yan et al., 2008). For instance, the research conducted by Anwar and colleagues demonstrated that upregulation of miR-21 was associated with poor PFS in BC patients (Anwar et al., 2019), and it refers to its potential role in targeting the genes including TIMP3, PDCD4, PTEN, TPM1, and RECK that are involved in BC promotion and progression, especially invasion, angiogenesis, and metastasis (Petrović, 2016). Another miR-21 target is LZTFL1, which is suppressed in the presence of miR-21 overexpression and contributes to cancer metastasis. Wang and colleagues conjectured that the miR-21/LZTFL1 axis might stimulate BC epithelial–mesenchymal transition (EMT) through β-catenin (Wang et al., 2019d). Furthermore, Arisan et al. (2021) showed that miR-21 expression promotes EMT by significantly increasing E-cadherin and reducing vimentin, SNAI1, and Zeb-1 in MDA-MB-231 BC cells. Inhibitors of matrix metalloproteinases (MMPs) are another factor that contributes to cancer metastasis by regulating the extracellular matrix (ECM). The tissue inhibitor of metalloproteinase 3 (TIMP3) displayed an inverse correlation with miR-21 in BC, suggesting that miR-21 may control BC invasion by regulating TIMP3 (Song et al., 2010). Therefore, miR-21 plays an essential role in breast cancer invasion and might be a strong prognostic and diagnostic biomarker. Additionally, our study revealed that miR-210 is strongly associated with the OS of BC with HR: 2.07 (1.34–3.20) and *p* = 0.00, but there is no link between DFS and miR-210 (*p* = 0.61). Indeed, overexpression of miR-210 can inhibit cell death and promote cell survival (Wang et al., 2014b). MiR-210 inhibits cell proliferation by targeting fibroblast growth factor receptor (FGFR)-like 1 (FGFRL1) and homeobox A1 (HOXA1) and controls the cell’s response to hypoxia. Hypoxia occurs as a result of growth and development of a malignant tumor and may make conventional therapies ineffective. miR-210 depends on hypoxic gene regulation as HIF-1α/VHL induces miR-210 expression in BC (Camps et al., 2008). Also, miR-210 targets E-cadherin, a hallmark of EMT, which leads to breast cancer stem cell metastasis, proliferation, and invasion (Huang et al., 2018). MALAT1 is another one of the most frequently studied ncRNAs, and its upregulation is associated with a low survival rate in different cancers (Huang et al., 2017b; Lin et al., 2018; Sun et al., 2020). Our results [HR: 1.66 (95% CI: 1.07–2.56), *p* = 0.00] are consistent with those of previous research studies. According to the survival of BC patients within three years, MALAT1 can predict the poor prognosis with 77% sensitivity and 95% specificity (Sun et al., 2020). The study conducted by Miao and colleagues demonstrated that MALAT1 siRNA significantly reduces cell proliferation and enhances apoptosis in the MDA-MB-231 cell line (Miao et al., 2016). MALAT1 plays a critical role in angiogenesis by increasing the expression of VEGF. It was shown that MALAT1 has a mutual interaction with miR-145, and the suppression of MALAT1 significantly enhanced miR-145 levels in MCF-7 cells. Hence, the contribution of MALAT1 to angiogenesis may be carried out by interacting with miR-145 (Huang et al., 2018). Moreover, the overexpression of MALAT1 in breast cancer stem cells (CSCs) influences the stem cell-like phenotypes by modulation of SOX2 (Zeng et al., 2018). Although previous studies showed the overexpression of HOTAIR is associated with poor survival and a poor response to chemotherapy in BC patients (Tang et al., 2019), our analysis did not reveal a significant association with poor OS. Importantly, the evidence shows that overexpression of HOTAIR is linked to radioresistance (Zhang et al., 2020b) and drug resistance (Li et al., 2019c) which may cause poor OS in BC patients.

Based on our study, ncRNAs have also shown promising diagnostic potential in detecting BC, with the overall pooled sensitivity, specificity, and AUC, respectively, being 0.80, 0.82, and 0.88, suggesting that ncRNAs achieved a relatively high overall accuracy for BC detection. DOR represents the ratio of the odds of a true positive *versus* false positive, and a DOR value below 1.0 indicates poor discriminating ability. In our data, the pooled DOR was presented as 18, indicating an acceptable discriminatory performance of ncRNAs for BCs. A combination of PLR and NLR was also calculated to obtain a more comprehensive view of their diagnostic accuracy; a PLR of 4.44 would mean BC patients were four times more likely to test positive for ncRNAs than non-BC individuals. Furthermore, the pooled NLR was measured to be 0.25, indicating that there is only a 25% chance of false-negative results. However, there was no difference between the potential of single ncRNAs and combined ncRNAs in distinguishing BC patients from healthy populations. A study by Tang et al. (2017) showed that the combination of one lncRNA with 47 miRNAs has the ability to distinguish BC patients with a sensitivity of 83%, a specificity of 80%, a PLR of 4.51, an NLR of 0.21, and a DOR of 24.77, which are in line with our results.

Among various sources of ncRNAs, our data showed that serum-based ncRNAs have better accuracy than plasma and tissue with 0.81 sensitivity and 0.82 specificity. Furthermore, we conducted subgroup analyses based on the following variables: sample size, AMSTAR score, miRNA and lncRNA profiling, and their performance as a panel or single biomarker. The results showed that the miRNAs with a sensitivity of 0.81, a specificity of 0.83, and a DOR of 22 had a higher diagnostic value than lncRNAs. According to miRNA profiling, the result demonstrated that combined miRNAs are more promising as biomarkers for BC detection than single miRNAs; however, the diagnostic potential of multiple and single lncRNAs had a slight difference. No evidence of publication bias is found in the current study.

Among the ncRNAs, miR-21 and miR-155 were studied more frequently than others. In our study, miR-21 with a sensitivity of 0.81 and a specificity of 0.83 showed a high potential as a BC biomarker. Previous studies reported that miR-21 expression was significantly increased in BC and could act as a potential candidate biomarker for BC, which is in accordance with our results (Adhami et al., 2018). For example, Anwar et al. revealed that the expression of miR-21 is 7-fold higher in BC patients than in healthy women, which confirmed the oncogenic feature of miR-21 in BC (Anwar et al., 2019). It was observed that overexpression of miR-21 leads to downregulation of the signal transducer and activator of transcription 3 (STAT3) (Zhang et al., 2016). MiR-155 had a pooled sensitivity and specificity of 0.85 and 0.84, respectively, for the diagnosis of breast cancer according to our study. Liu et al. (2013) showed that the expression of miR-155 is 3-fold higher in breast tissue than in healthy adjacent tissue. Mir-155 targets the tumor suppressor gene, *suppressor of cytokine signaling 1* (SOCS1), in BC cells and negatively regulates SOCS expression. SOCS1 is a negative regulator of cytoplasmic Janus-activated kinase (JAK) and STAT3 signaling, that is, upregulation of miR-155 in BC cells activates STAT3 through the JAK pathway. Also, inflammatory cytokines such as IFN-γ and interleukin-6 (IL-6), lipopolysaccharide (LPS), and polyriboinosinic:polyribocytidylic acid [poly(I:C)] stimulate BC cells and cause the overexpression of miR-155, implying a role for miR-155 as a bridge between inflammation and cancer (Jiang et al., 2010). Moreover, increased expression of miR-155 significantly inhibits cell apoptosis, and the decreased level of miR-155 correlates with cell apoptosis. Cell adhesion molecule 1 (CADM1) is a tumor suppressor that is inactivated in most reported BC research, and its inactivation is closely associated with patients’ poor prognosis and advanced cancer progression. Zhang et al. (2019) reported that miR-155 expression downregulates CADM1 expression, and this may promote BC progression *via* CADM1 downregulation. The previous study (Han et al., 2017) indicated that the combination of miR-155 and miR-21 can diagnose breast cancer patients with a sensitivity of 96.6% and a specificity of 66.67%.

To gain insight into the molecular functions of the four miRNAs, we predicted the target genes and analyzed the related pathways and GO analysis. The abnormal signal pathway plays an important role in the occurrence and development of breast cancer. We found that these genes (VEGFA, SMAD4, BTG2, E2F3, SMAD7, UBR5, and IRAK1) have roles in a number of important signaling pathways, including the cell cycle, TGF-beta, AGE-RAGE, and hippo signaling pathways. The canonical TGF-beta/SMAD4 signaling pathway suppresses tumors in the early stages, primarily by inducing cell cycle arrest and apoptosis. SMAD4 acts at the G1/S checkpoint to keep cells in the G1 phase, resulting in cell cycle arrest (Zhao et al., 2018). According to Liu and colleagues, SMAD4 may play a role in the progression of breast cancer and be used as a prognostic indicator for the disease (Liu et al., 2014b). The TGF-beta pathway has been linked to the growth and progression of breast tumors according to earlier research. SMAD4 is necessary for TGF-beta signal transduction (De Kruijf et al., 2013). In addition, the contribution of UBR5 to poor prognosis was confirmed by upregulation of β-catenin expression and activity in ER+ breast cancer (Yang et al., 2020). The research by Mota et al. is in line with the findings of a pathway analysis that shows the role of SMAD-hippo signaling in breast cancer (Mota et al., 2018).

Furthermore, as shown in Figure 4C, these target genes were mostly involved in the regulation of the RNA polymerase II promoter, transcription, cell differentiation, and a variety of bindings. These biological processes and signaling pathways suggest that the target genes play a key role in the progression of breast cancer.

Taking everything mentioned earlier into account, these lncRNAs, miRNAs, and their predicted targets in pan-cancer data showed significantly different expression in normal and cancer patients (Figure 5, Supplementary Figures S14, S15). They were also significantly associated with poor and good prognoses with OS and RFS (Figure 6, Supplementary Figures S16, S17), suggesting that they could serve as a panel of good prognostic biomarkers for breast cancer patients.

Our study has some advantages that are worth mentioning. First, our work has been updated on 8 January 2022. Second, the work described the effectiveness of single ncRNAs and a panel of ncRNAs as non-invasive prognostic and diagnostic biomarkers in breast cancers, which have not been published before. Third, we included all studies related to this area without applying any restrictions. Analyzing the potential of ncRNAs in all sample sources, different ethnicities, and as single biomarkers and panels overly and based on RNA type makes our study more comprehensive and unique. More importantly, this umbrella review is the first and most comprehensive systematic review of systematic reviews and meta-analyses on ncRNAs that have the potential to be used as prognostic and diagnostic biomarkers. The robustness and validity of the included studies were strictly rated based on the assessment results of a series of statistical analyses. The methodological qualities of the systematic reviews included were assessed using the AMSTAR 2.0 checklist, which is a major update of the former version of AMSTAR.

We should also mention one of our study’s limitations, which is the lack of data related to each subtype of breast cancer. Most research studies have focused on breast cancer as a single disease; nevertheless, it is critical to validate a biomarker or a panel of biomarkers for each subtype. Patients would have the appropriate treatment option if early non-invasive testing could also determine the subtype. As a result, it may improve their quality of life and survival rate, so it offers more advantages if future studies, including original articles, systematic reviews, and meta-analyses, focus on one specific subtype of breast cancer to monitor and validate non-invasive biomarkers.

## 5 Conclusion

Taken together, our findings indicate that non-coding RNAs, such as miRNAs, lncRNAs, and cricRNAs, have the potential to be used as non-invasive biomarkers for breast cancer detection. Because there is no difference in the potential of ncRNAs as single biomarkers *versus* panels, finding a single one to use as a non-invasive tool would be more cost-effective. Our findings demonstrate that miRNAs have a higher diagnostic accuracy than lncRNAs. Although this could be due to the fact that most studies focused on miRNAs, we recommend that future studies focus on their potential rather than lncRNAs, as they are easier to work with and more stable in different body fluids than lncRNAs.

## Data Availability

The original contributions presented in the study are included in the article/Supplementary Material; further inquiries can be directed to the corresponding authors.

## Glossary

AMSTAR: A Measurement Tool to Assess Systematic Reviews
AUC: area under curve
BC: breast cancer
BP: biological pathways
CA15-3: carbohydrate antigen 15-3
CC: cellular component
CEA: carcinoembryonic antigen
CI: confidence interval
circRNA: circular RNA
CSS: cancer-specific survival
DFS: disease-free survival
DOR: diagnostic odds ratio
DRFS: distant relapse-free survival
DSS: disease-specific survival
ER: estrogen receptor
FGFRL1: fibroblast growth factor receptor (FGFR)-like 1
FNs: false negatives
FPs: false positives
GO: Gene Ontology
HOXA1: homeobox A1
HR: hazard ratio
lncRNA: long non-coding RNA
MF: molecular function
MFS: metastasis-free survival
miRNA: microRNA
ncRNAs: non-coding RNAs
NLR: negative likelihood ratio
OR: odds ratio
OS: overall survival
PFS: progression-free survival
PLRs: positive likelihood ratios
PR: progesterone receptor
RFS: relapse-free survival
RR: relative risk
SENS: sensitivity
SPEC: specificity
SROC: summary receiver operating characteristic curve
TNs: true negatives
TPs: true positives.
