# Supplementary figures and images for: Prognostic and diagnostic values of non-coding RNAs as biomarkers for breast cancer: An umbrella review and pan-cancer analysis
Source: Front Mol Biosci. 2023 Jan 16;10:1096524. doi: 10.3389/fmolb.2023.1096524 (PMC9885171; doi:10.3389/fmolb.2023.1096524)

Identification

Screening

Eligibility

Included

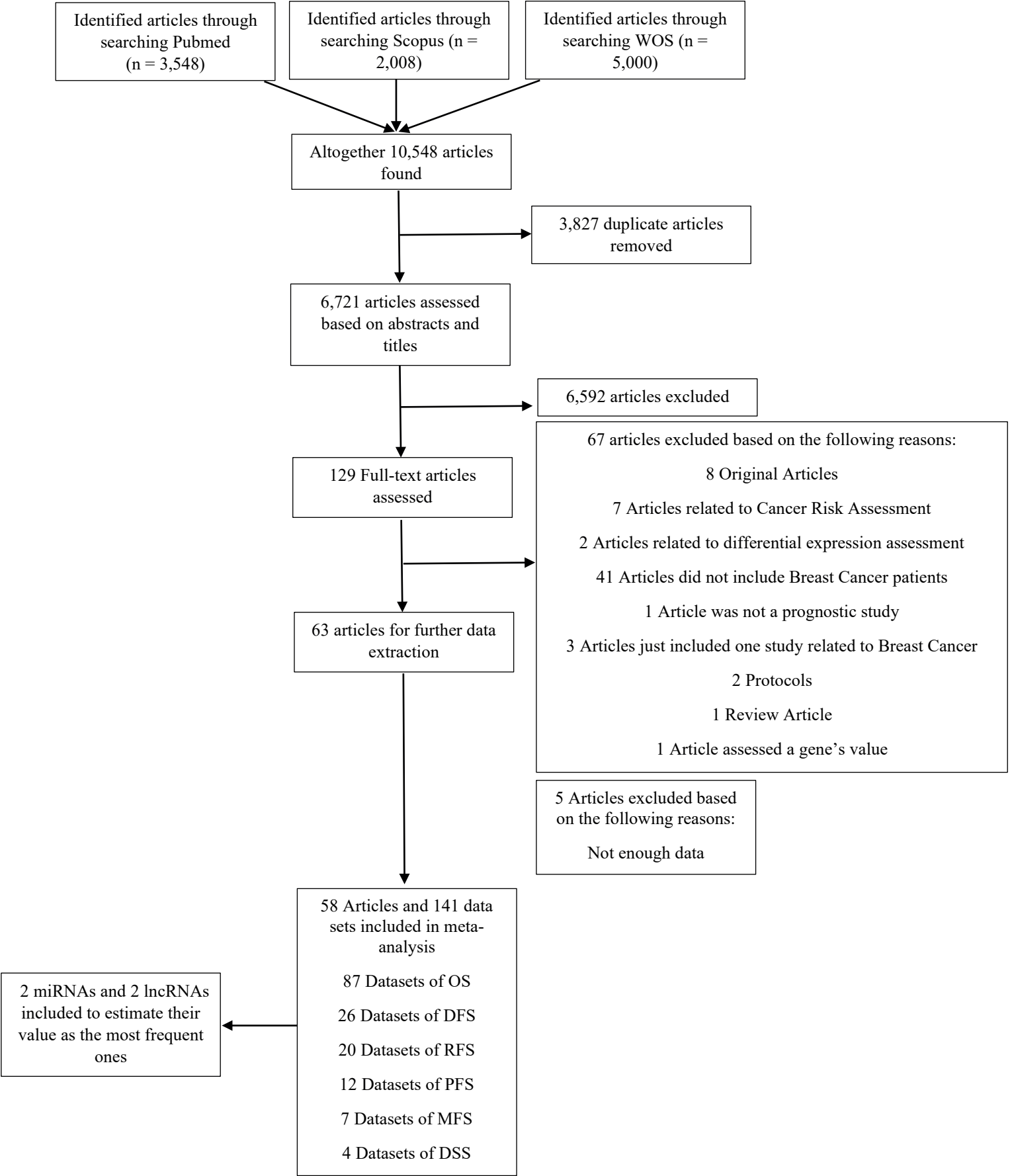

Supplement: Supplementary file 1 [file DataSheet1.ZIP › Supplementary Material, Fig 1.pdf]

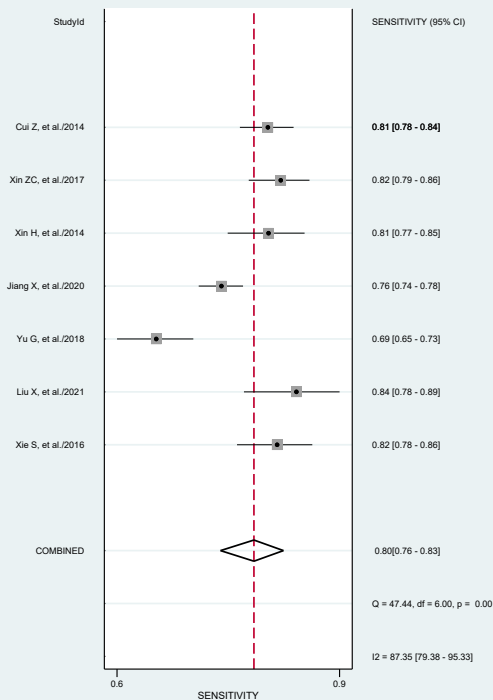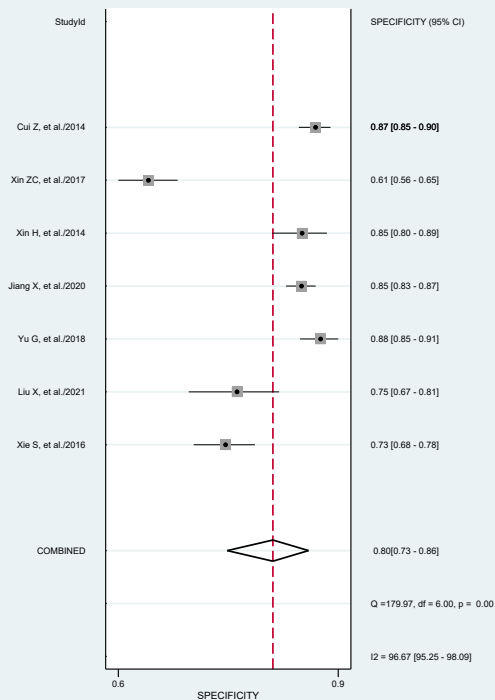

Supplement: Supplementary file 1 [file DataSheet1.ZIP › Supplementary Material, Fig 10a.pdf]

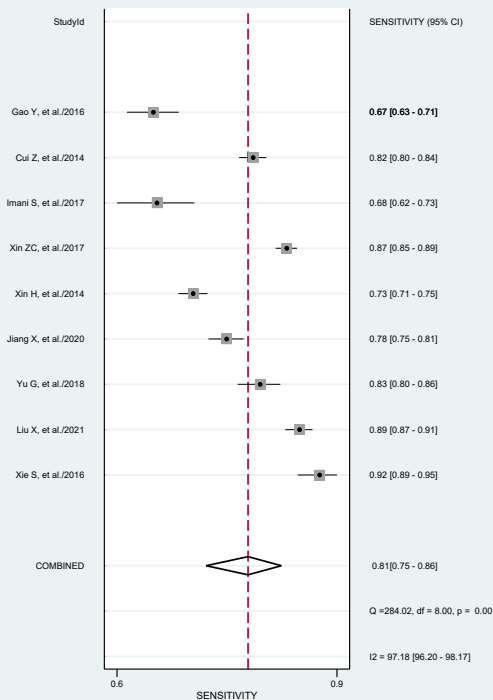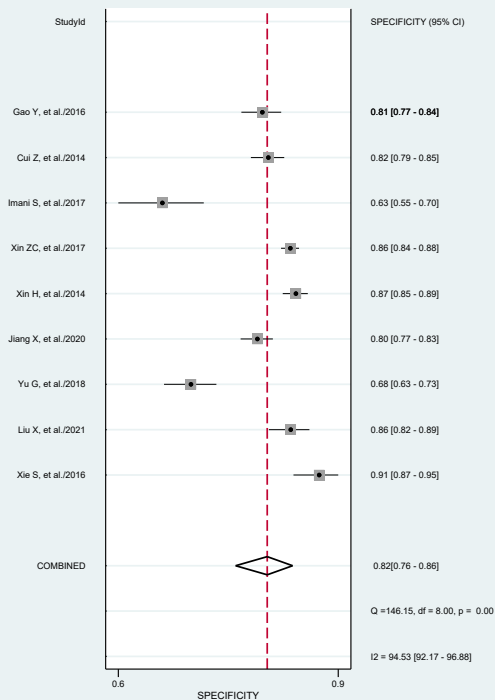

Supplement: Supplementary file 1 [file DataSheet1.ZIP › Supplementary Material, Fig 10b.pdf]

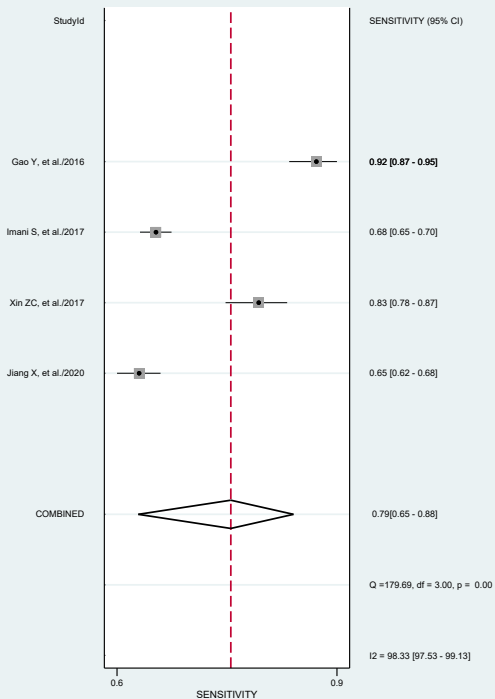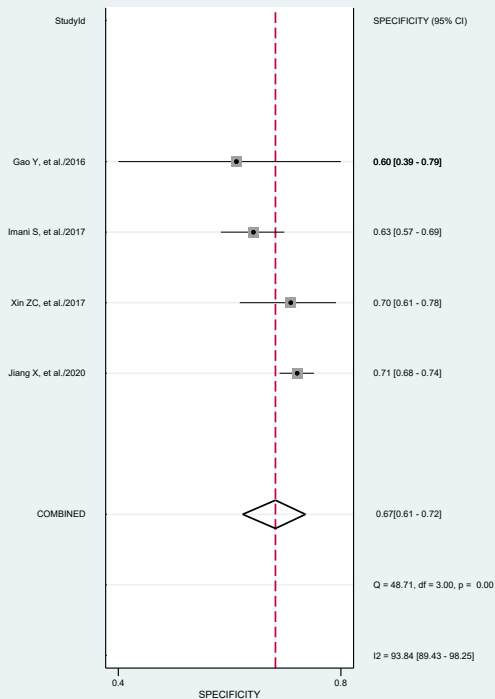

Supplement: Supplementary file 1 [file DataSheet1.ZIP › Supplementary Material, Fig 10c.pdf]

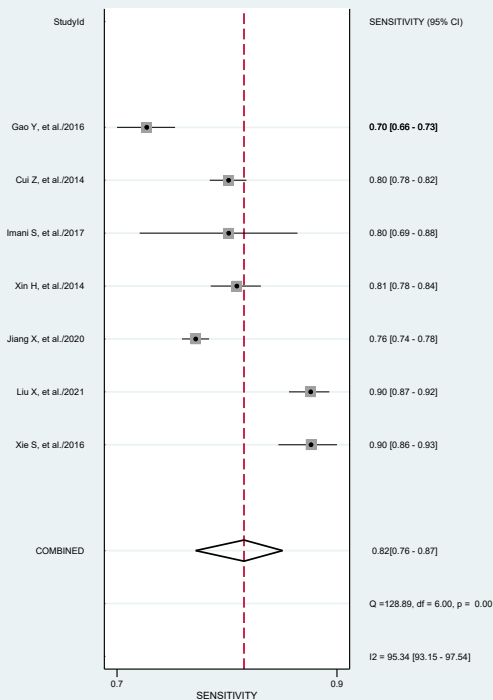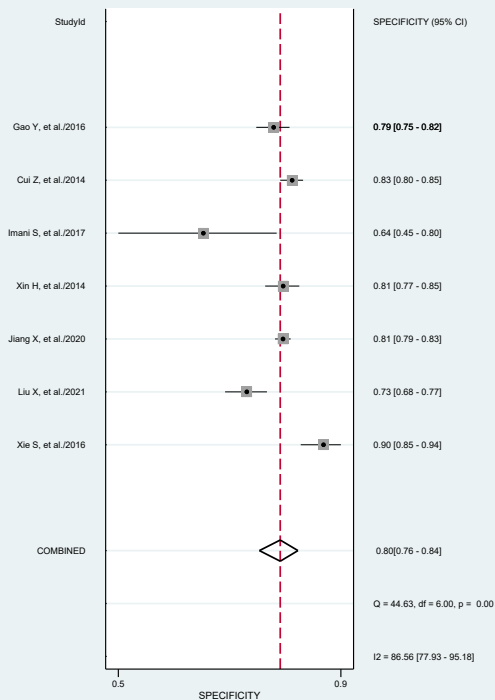

Supplement: Supplementary file 1 [file DataSheet1.ZIP › Supplementary Material, Fig 10d.pdf]

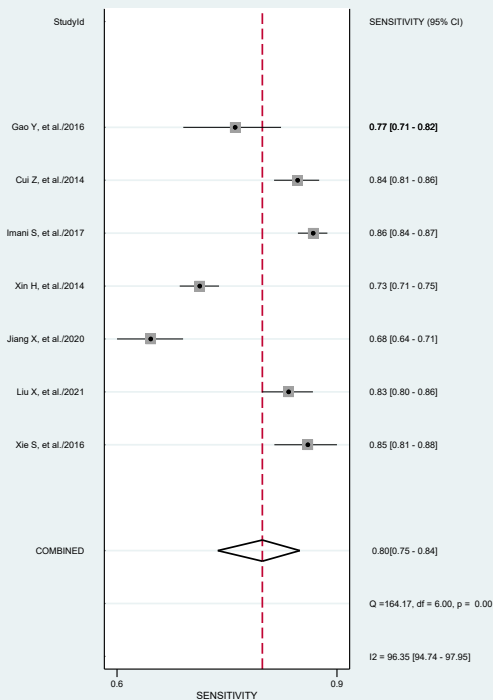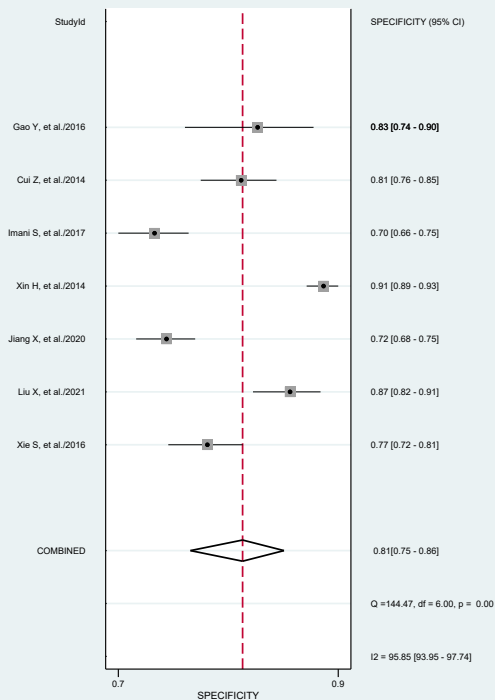

Supplement: Supplementary file 1 [file DataSheet1.ZIP › Supplementary Material, Fig 10e.pdf]

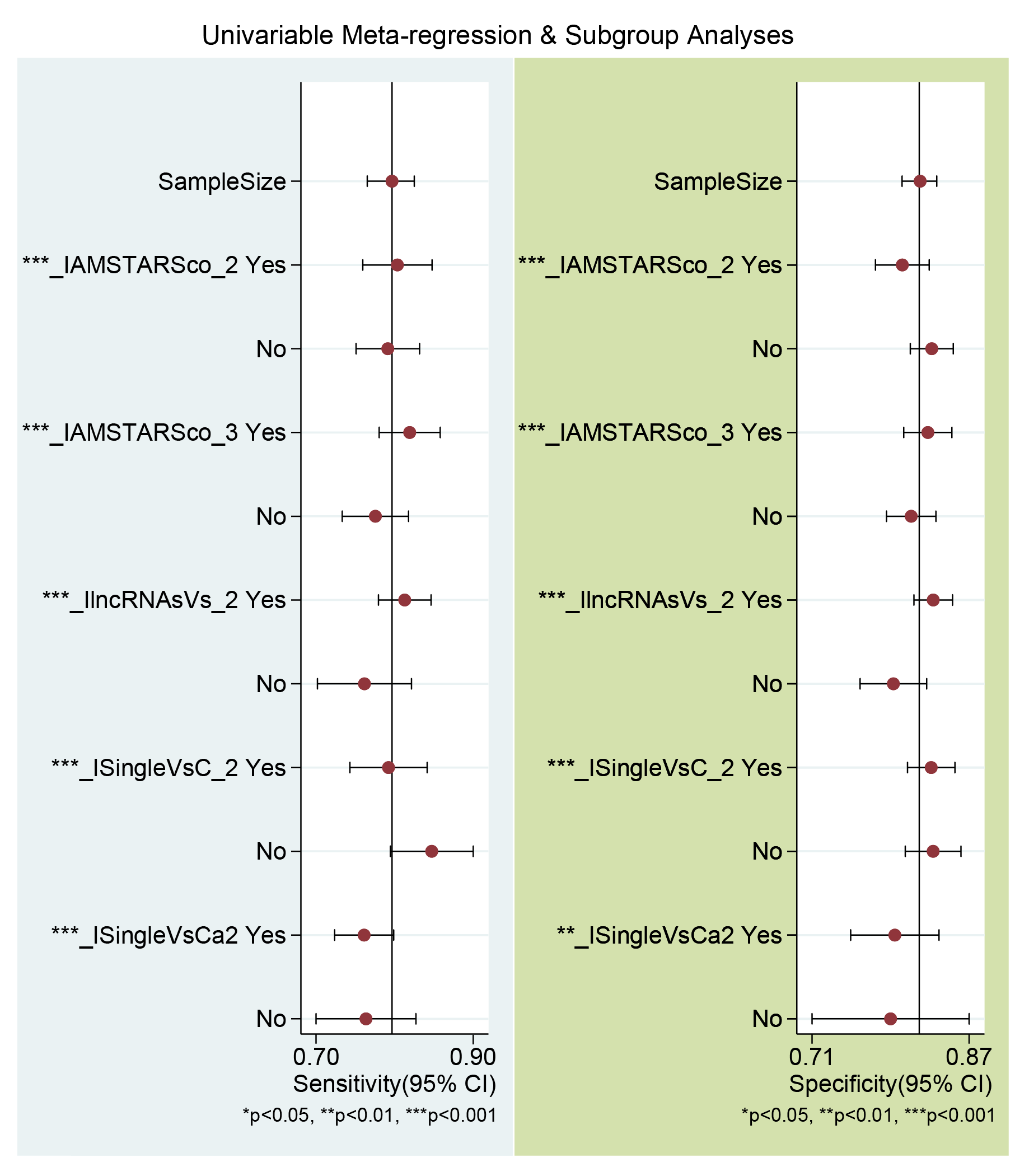

Supplement: Supplementary file 1 [file DataSheet1.ZIP › Supplementary Material, Fig 11.tif]

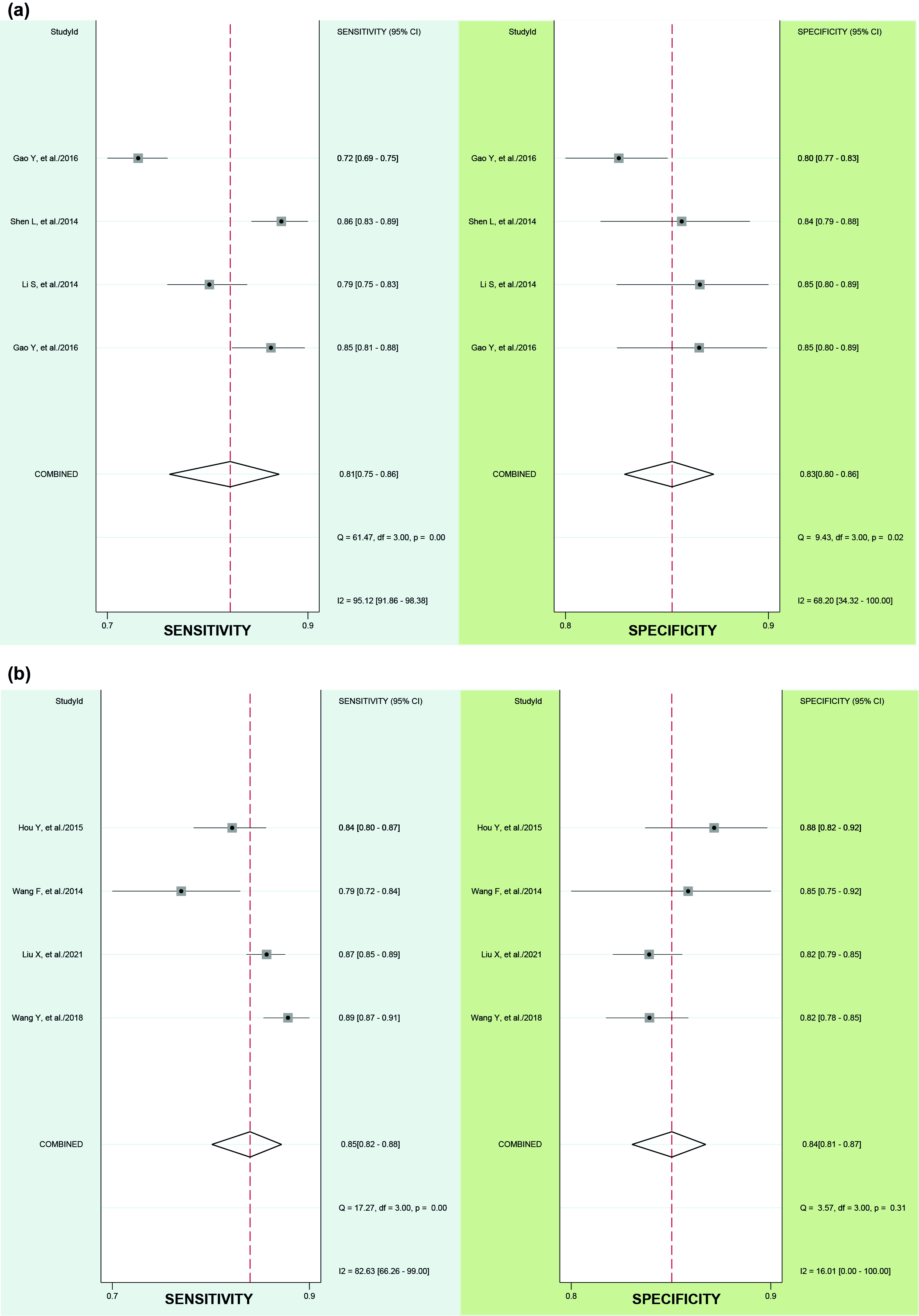

Supplement: Supplementary file 1 [file DataSheet1.ZIP › Supplementary Material, Fig 12.tif]

Deeks' Funnel Plot Asymmetry Test  
pvalue = 0.56

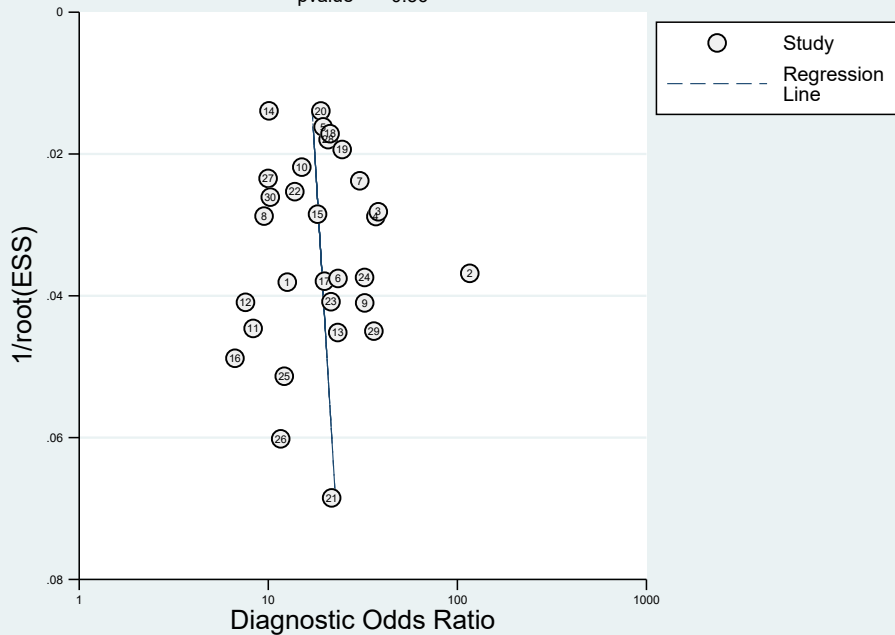

Supplement: Supplementary file 1 [file DataSheet1.ZIP › Supplementary Material, Fig 13.pdf]

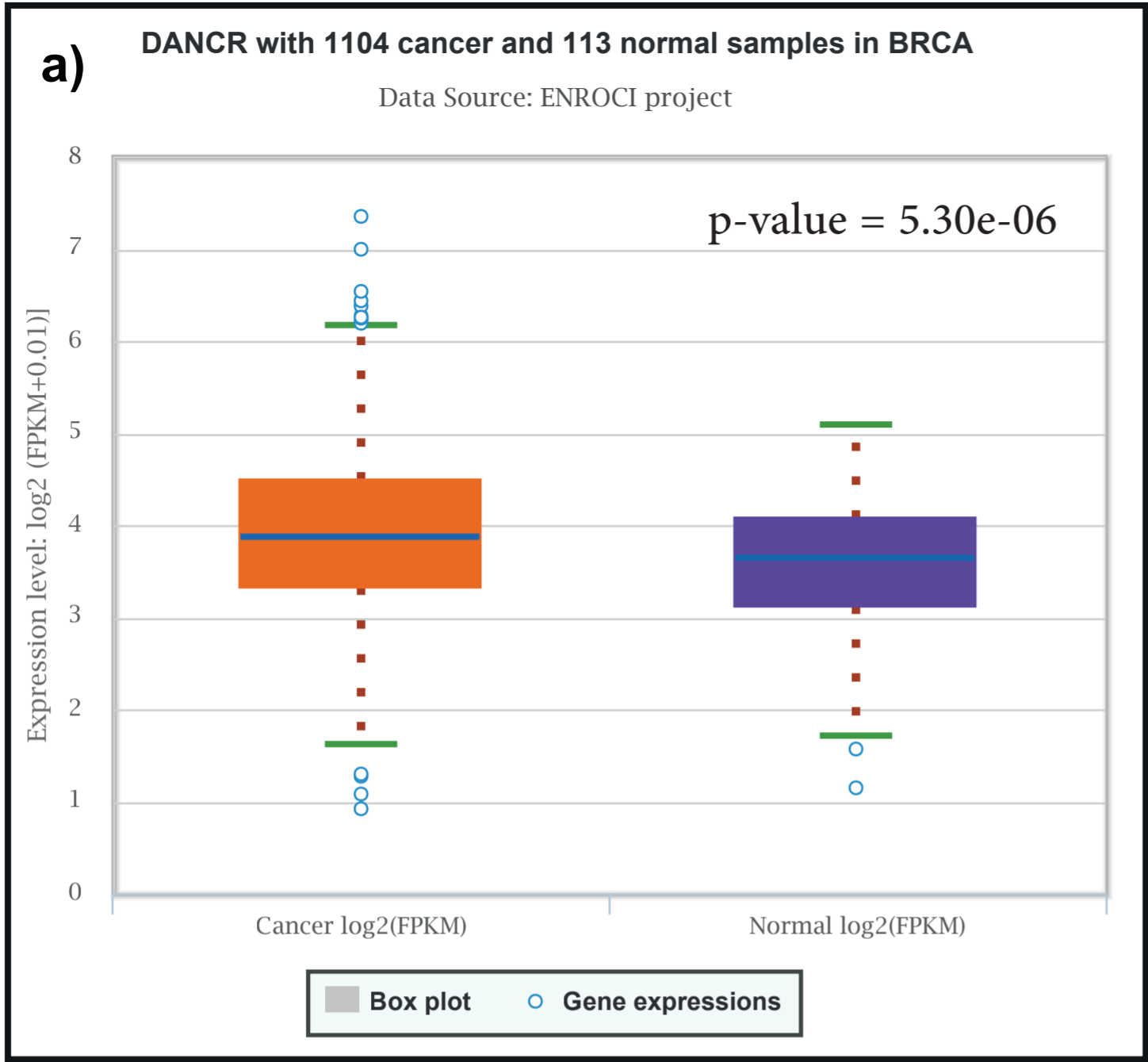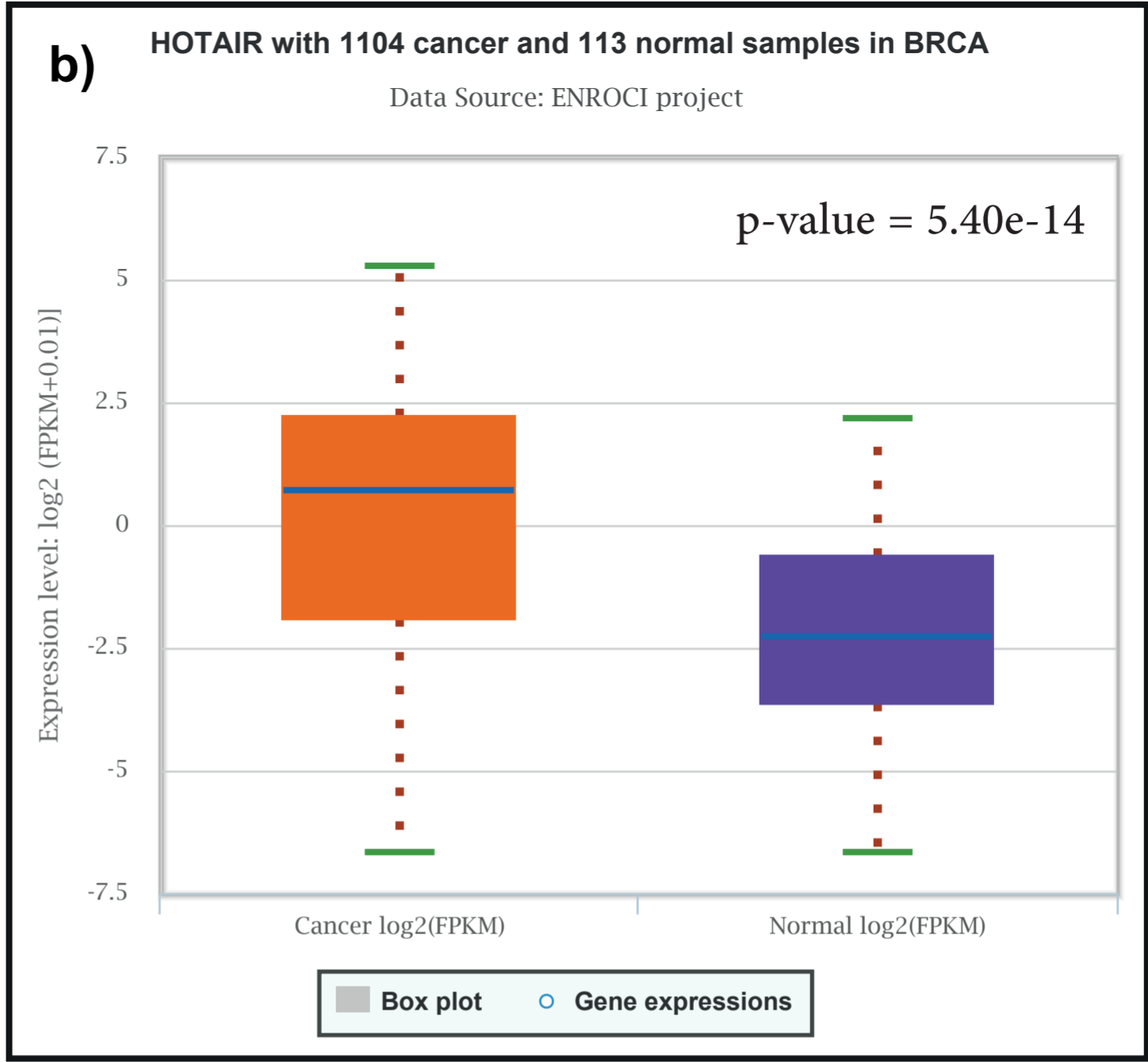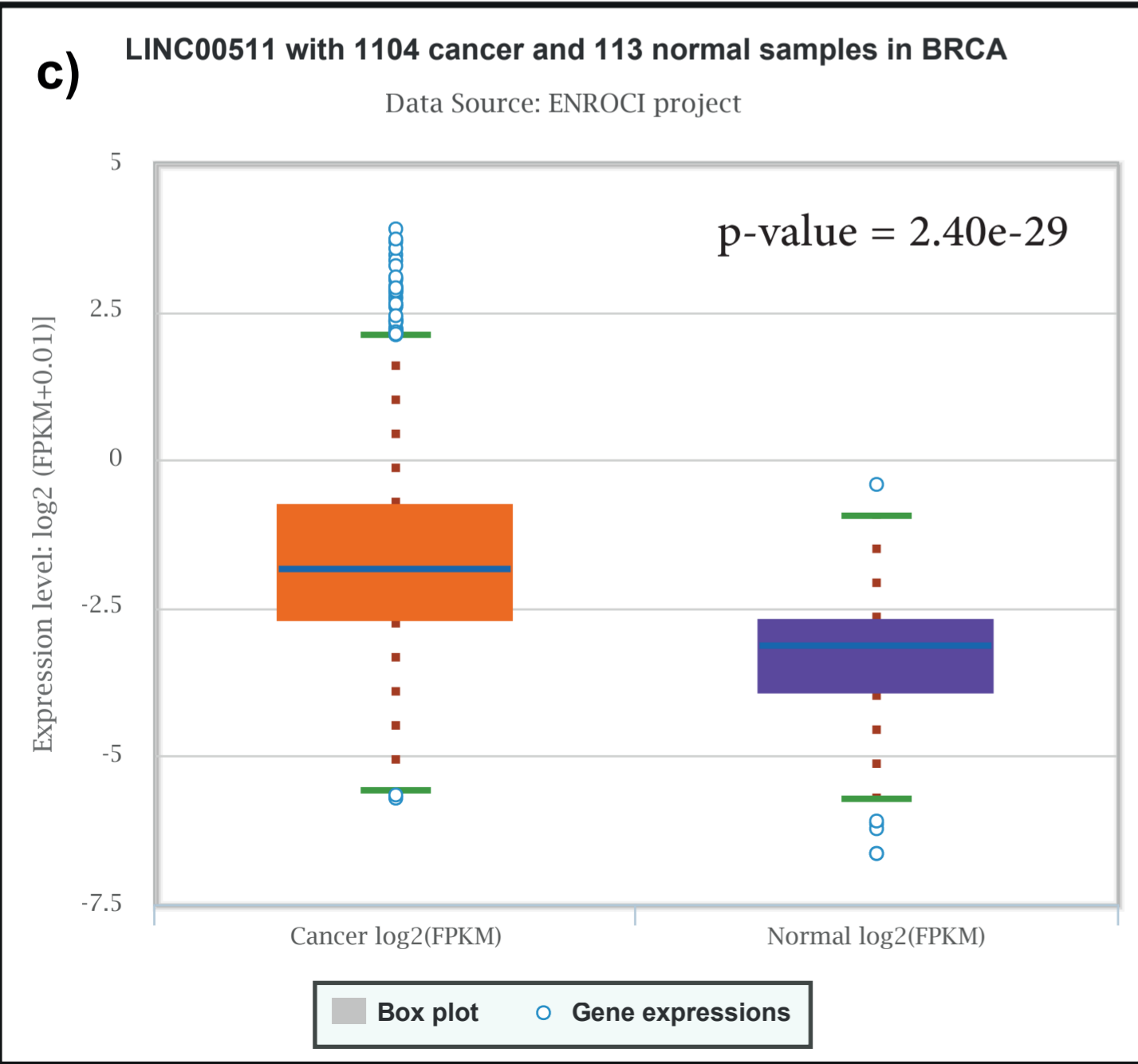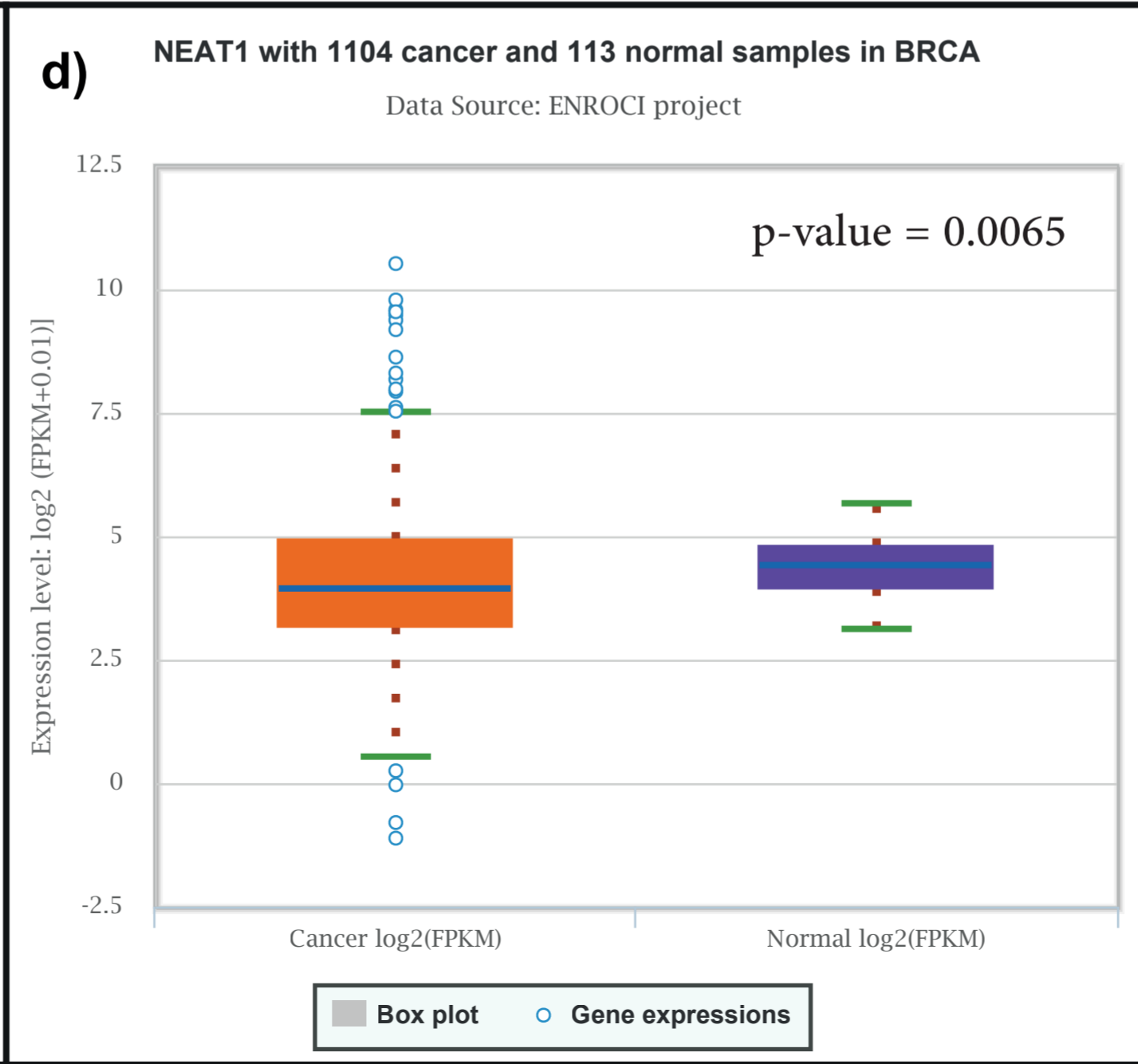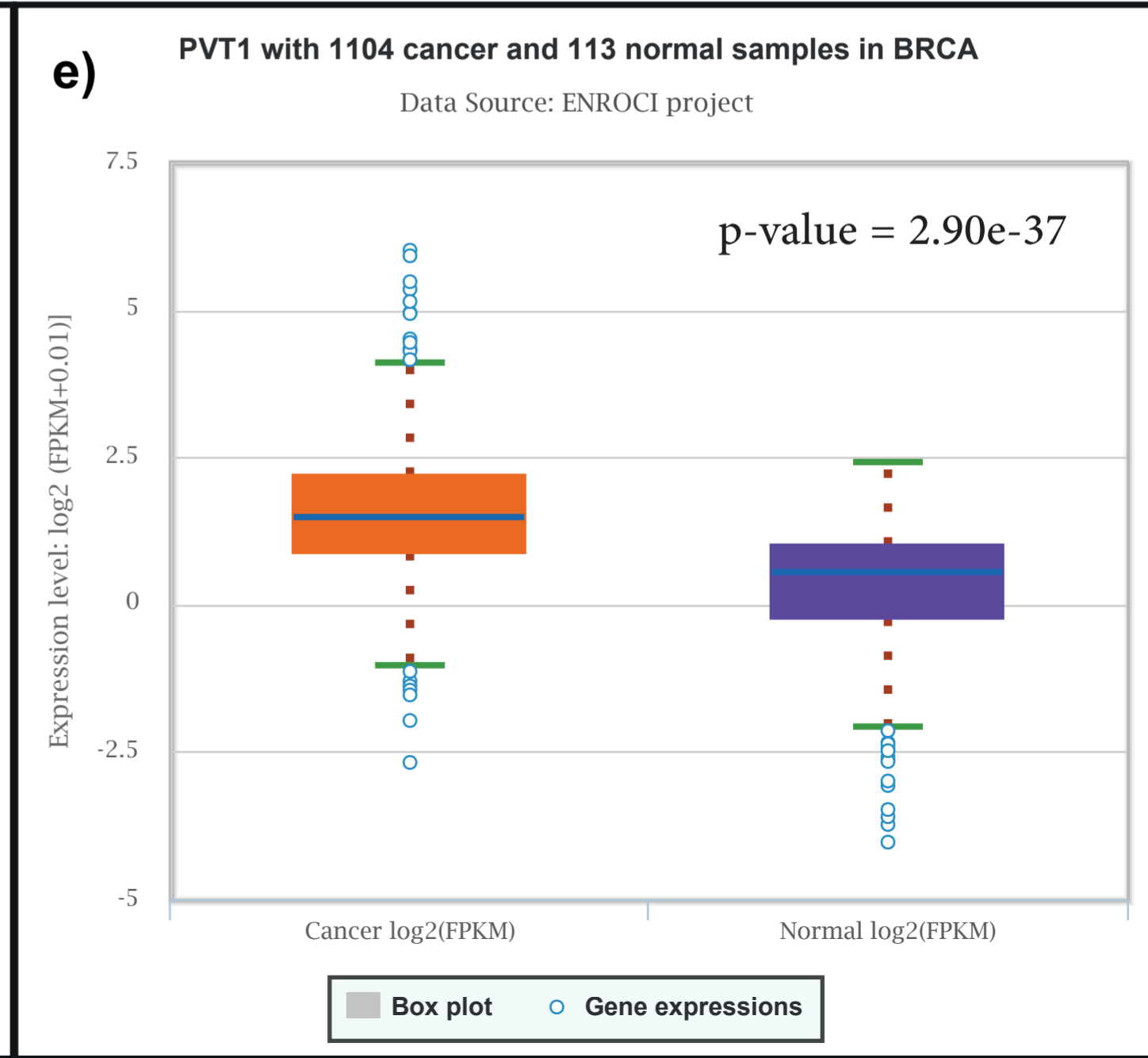

Supplement: Supplementary file 1 [file DataSheet1.ZIP › Supplementary Material, Fig 14.pdf]

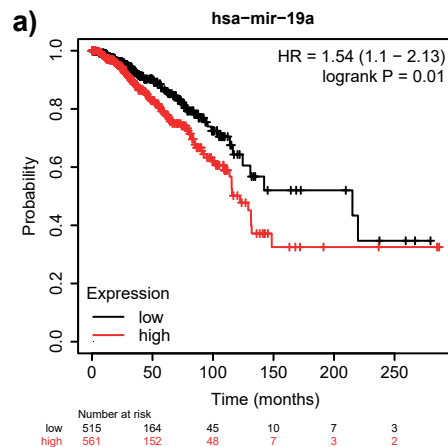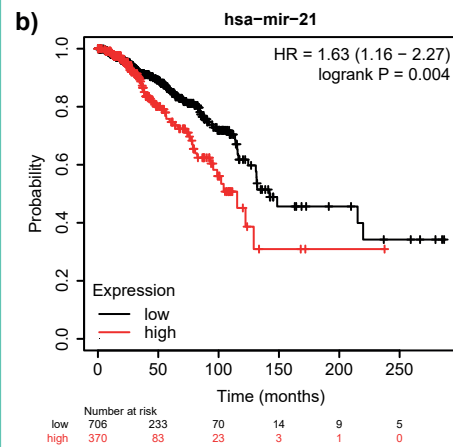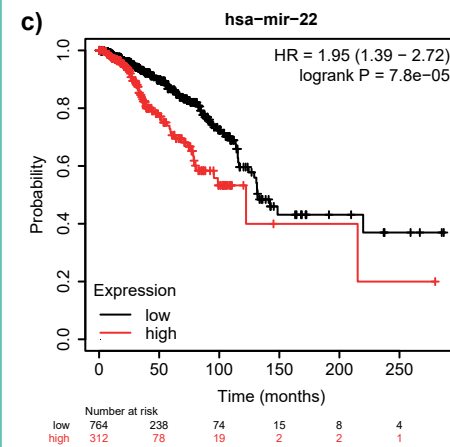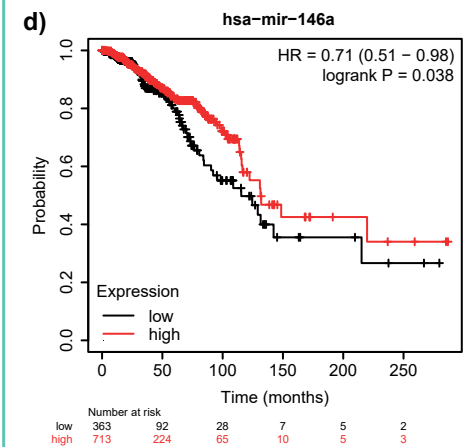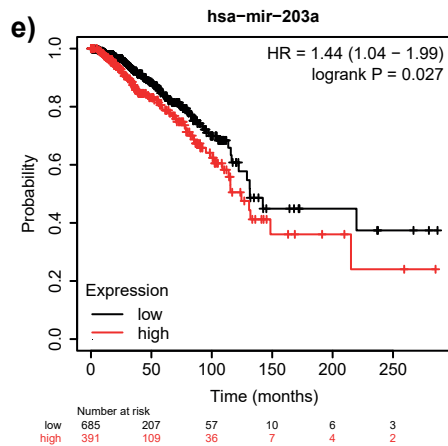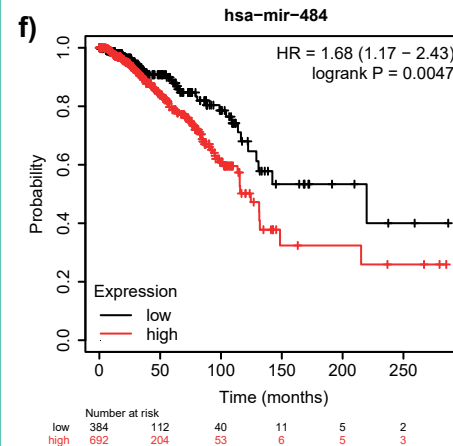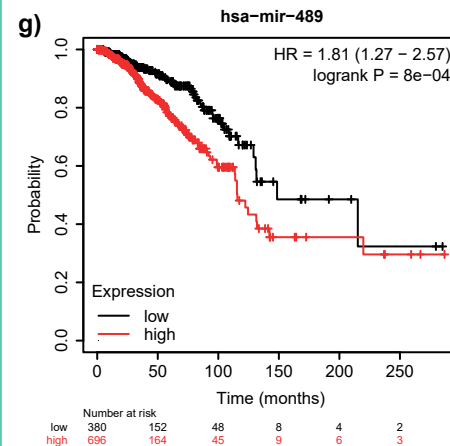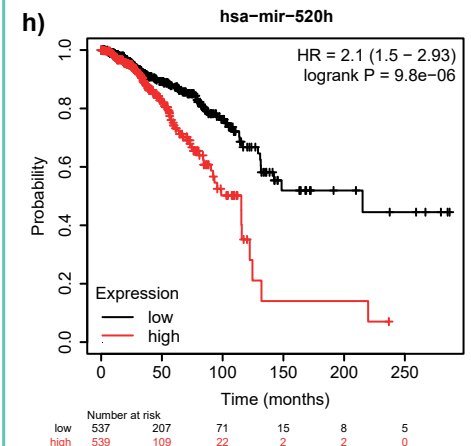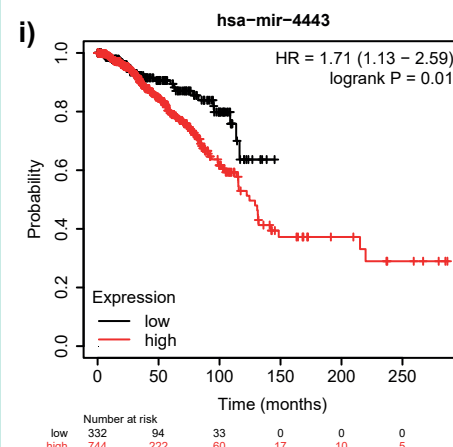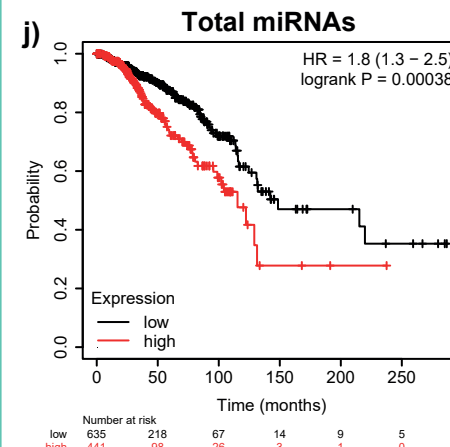

Supplement: Supplementary file 1 [file DataSheet1.ZIP › Supplementary Material, Fig 16.pdf]

OS

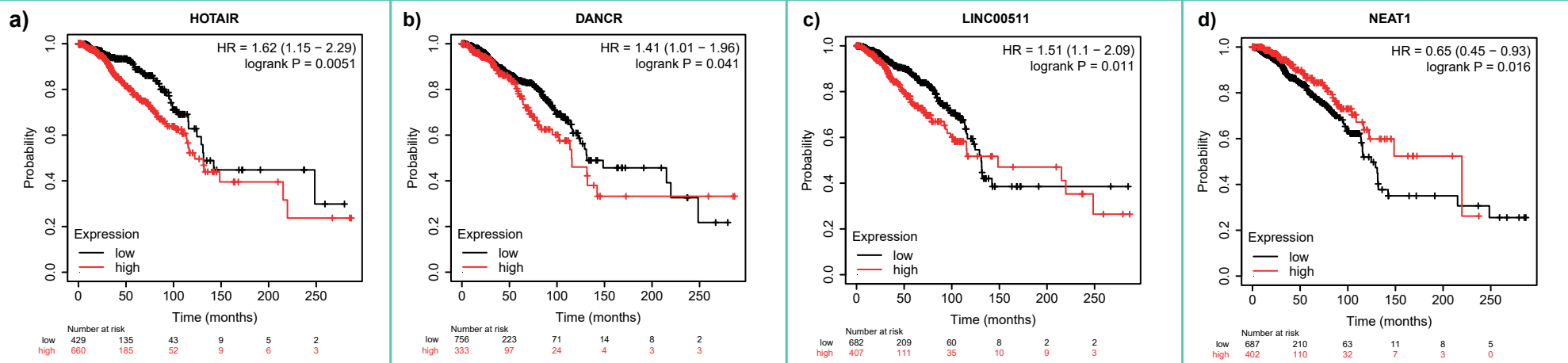

RFS

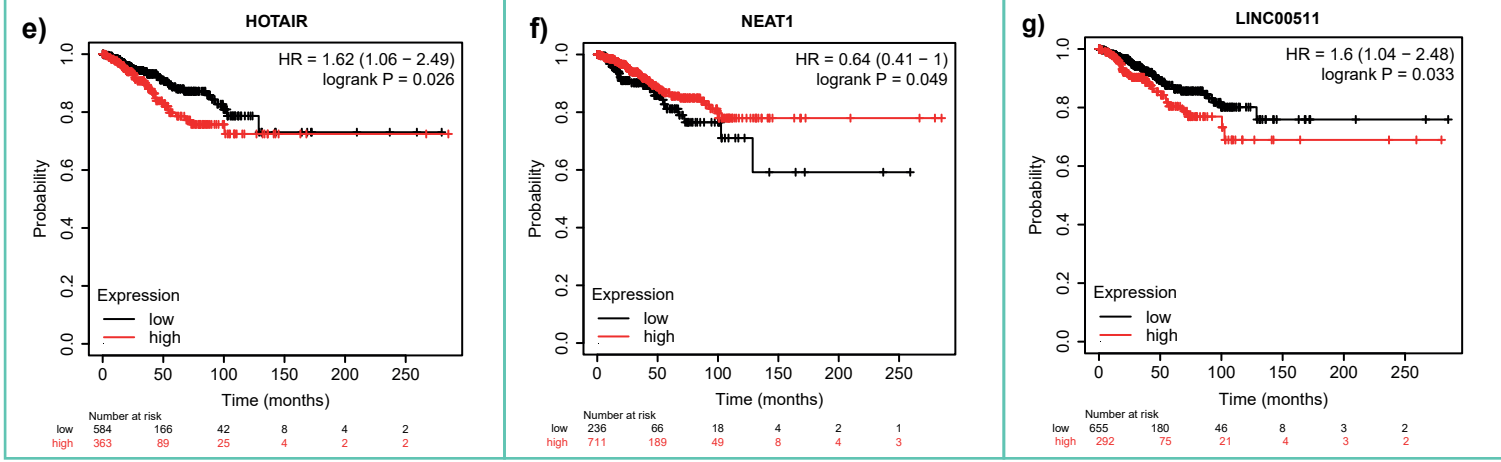

Supplement: Supplementary file 1 [file DataSheet1.ZIP › Supplementary Material, Fig 17.pdf]

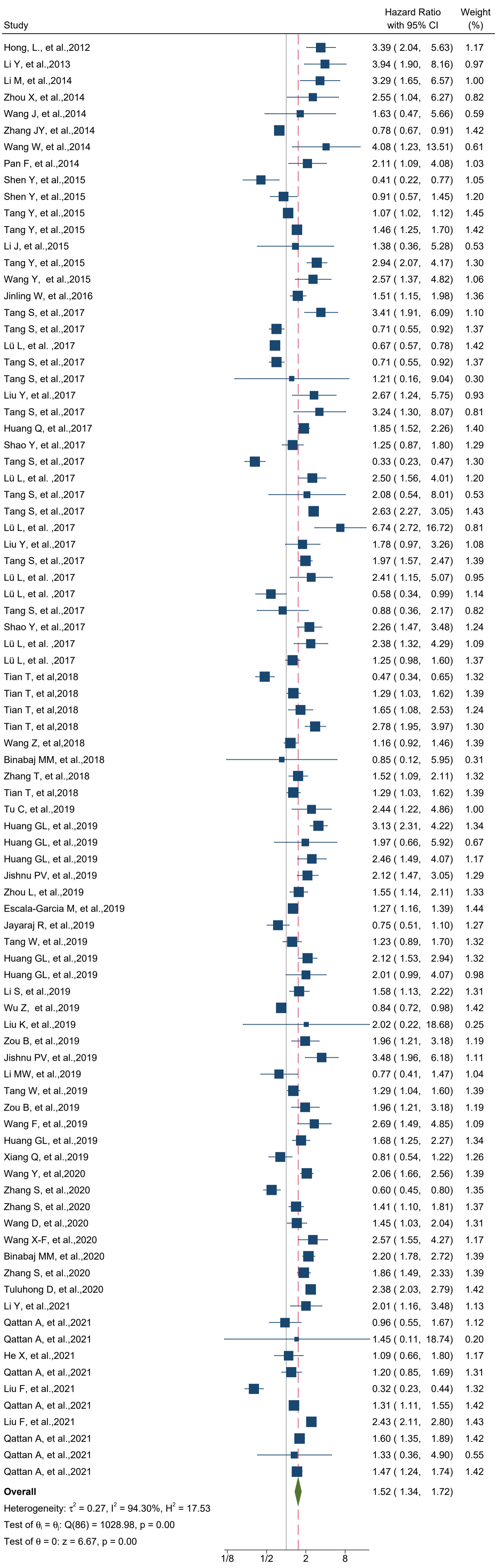

Supplement: Supplementary file 1 [file DataSheet1.ZIP › Supplementary Material, Fig 2a.pdf]

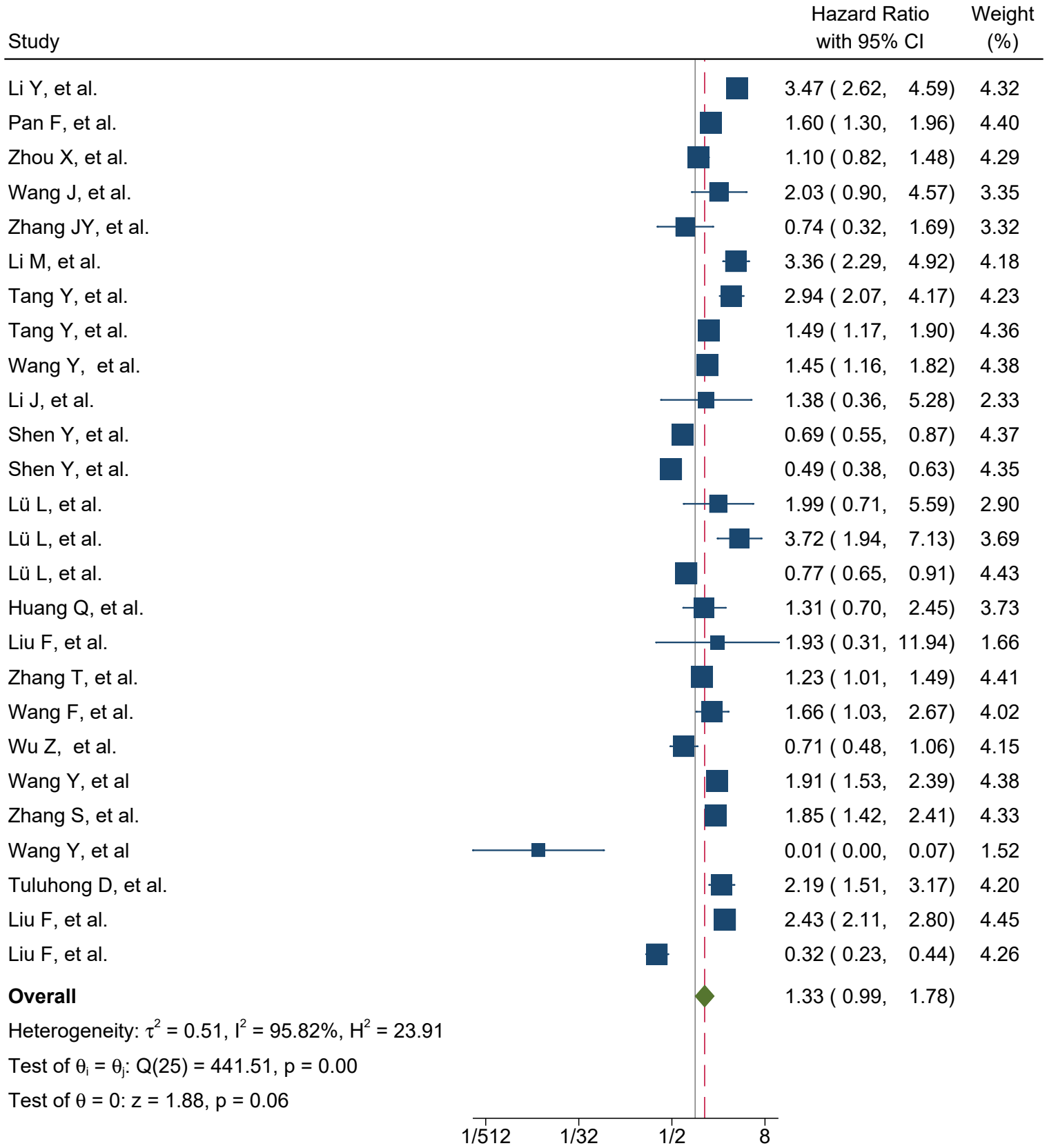

Random-effects REML model

Supplement: Supplementary file 1 [file DataSheet1.ZIP › Supplementary Material, Fig 2b.pdf]

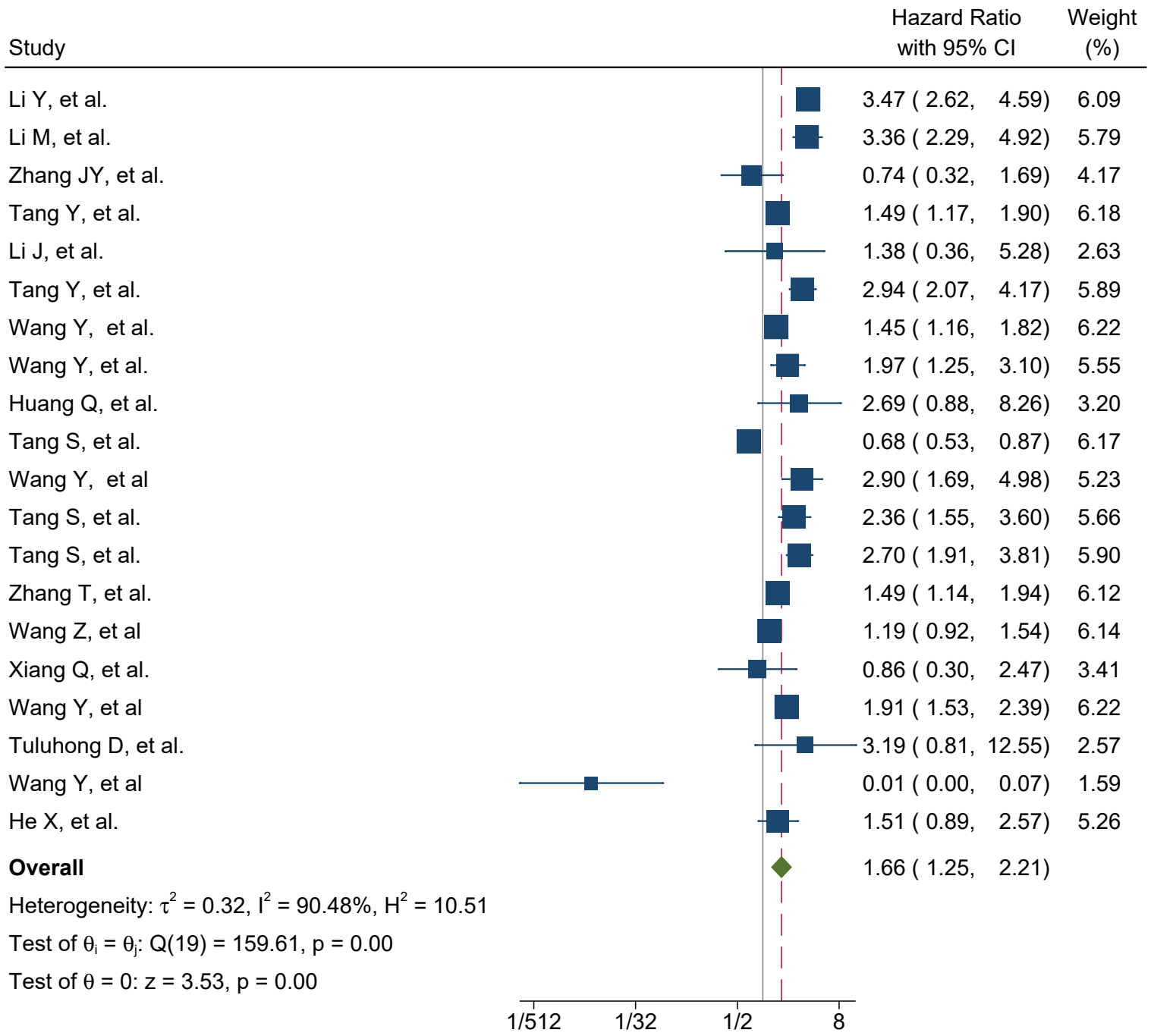

Random-effects REML model

Supplement: Supplementary file 1 [file DataSheet1.ZIP › Supplementary Material, Fig 2c.pdf]

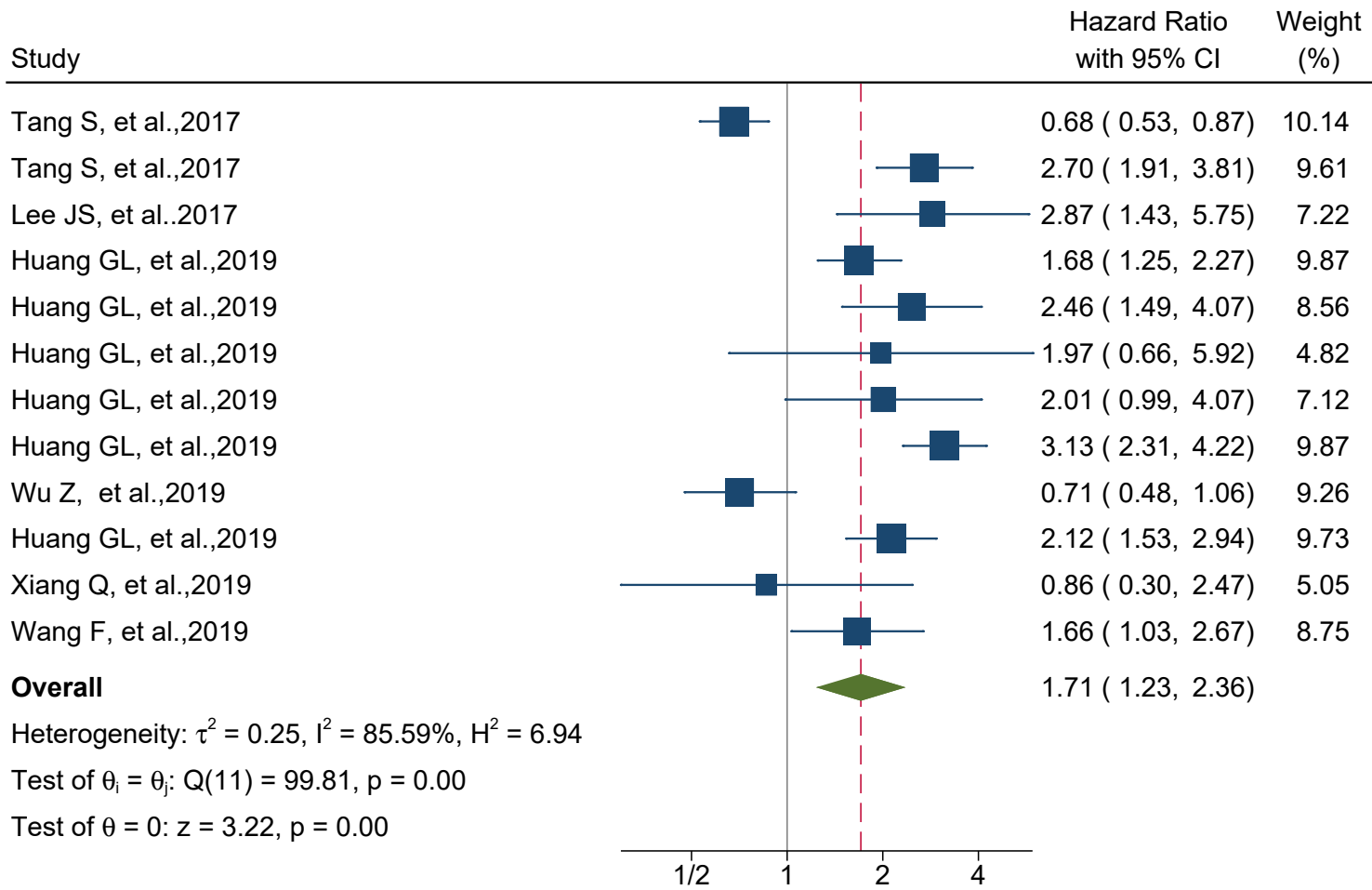

Random-effects REML model

Supplement: Supplementary file 1 [file DataSheet1.ZIP › Supplementary Material, Fig 2d.pdf]

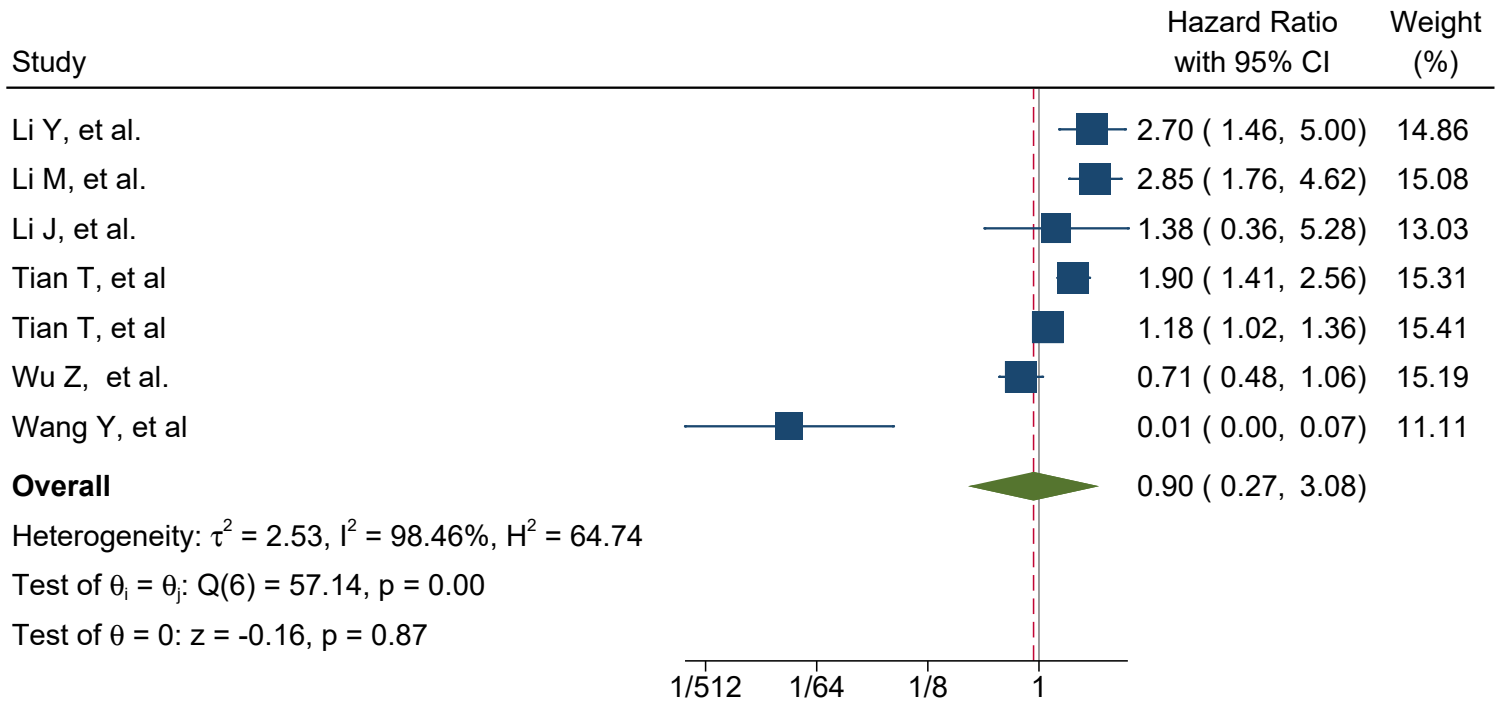

Random-effects REML model

Supplement: Supplementary file 1 [file DataSheet1.ZIP › Supplementary Material, Fig 2e.pdf]

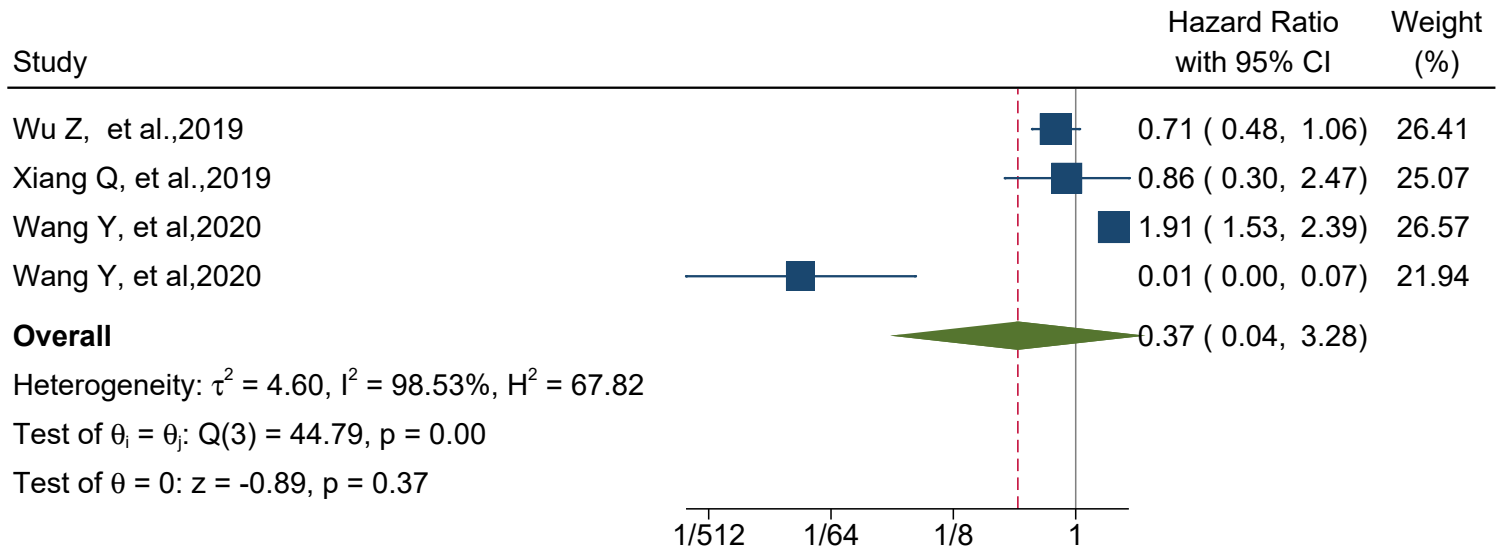

Heterogeneity:  $\tau^2 = 4.60$ ,  $I^2 = 98.53\%$ ,  $H^2 = 67.82$   
Test of  $\theta_i = \theta_j$ :  $Q(3) = 44.79$ ,  $p = 0.00$   
Test of  $\theta = 0$ :  $z = -0.89$ ,  $p = 0.37$

Supplement: Supplementary file 1 [file DataSheet1.ZIP › Supplementary Material, Fig 2f.pdf]

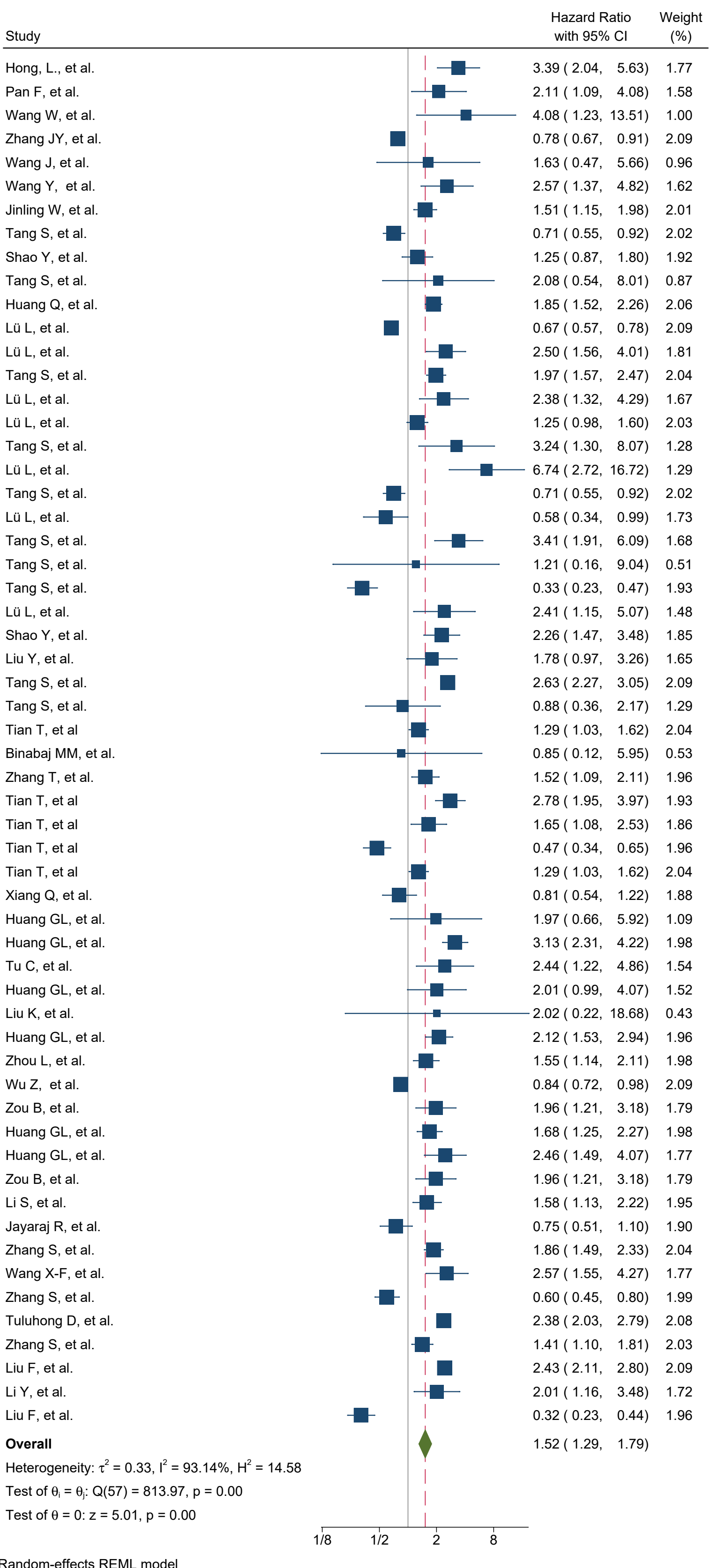

Supplement: Supplementary file 1 [file DataSheet1.ZIP › Supplementary Material, Fig 3a.pdf]

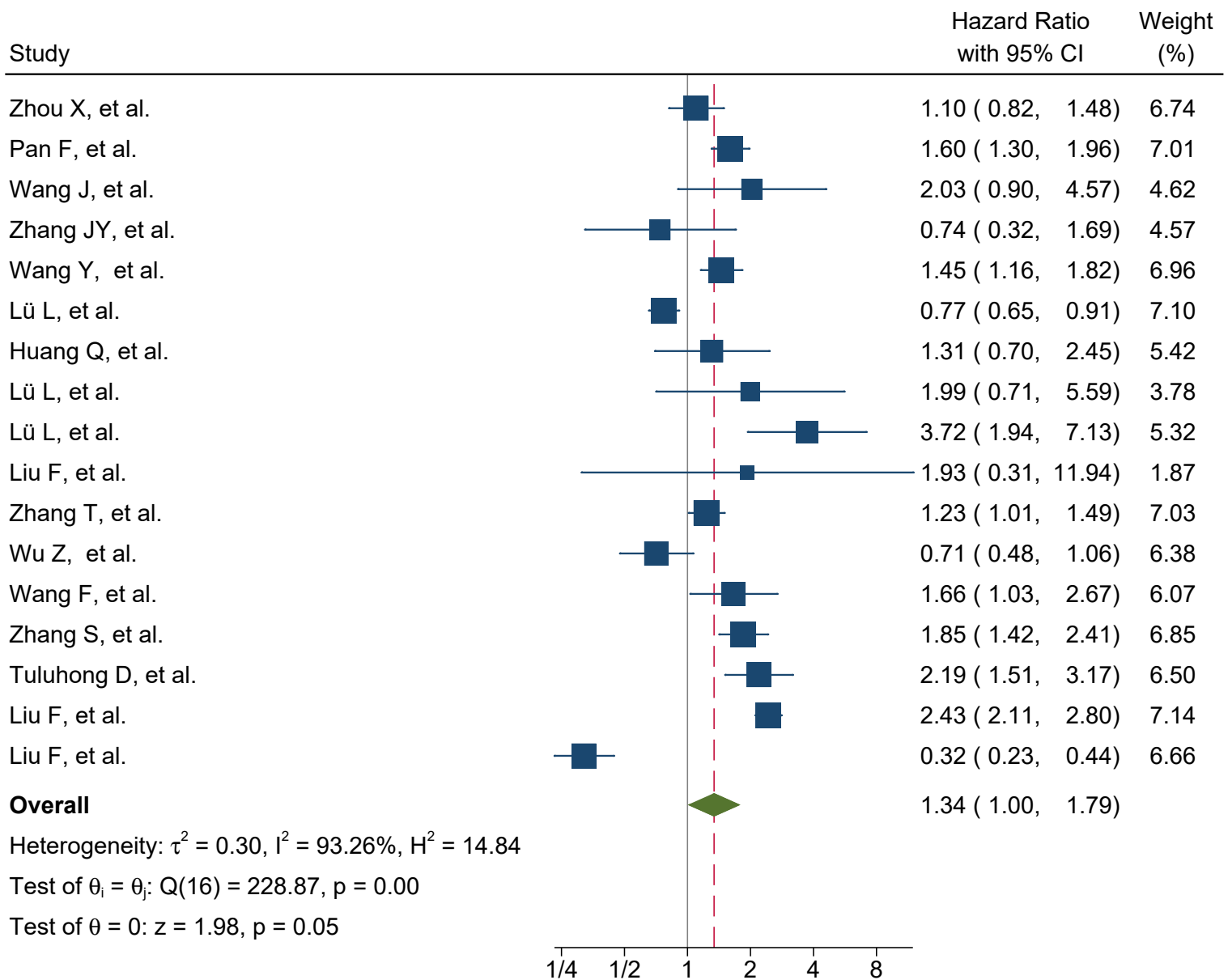

Random-effects REML model

Supplement: Supplementary file 1 [file DataSheet1.ZIP › Supplementary Material, Fig 3b.pdf]

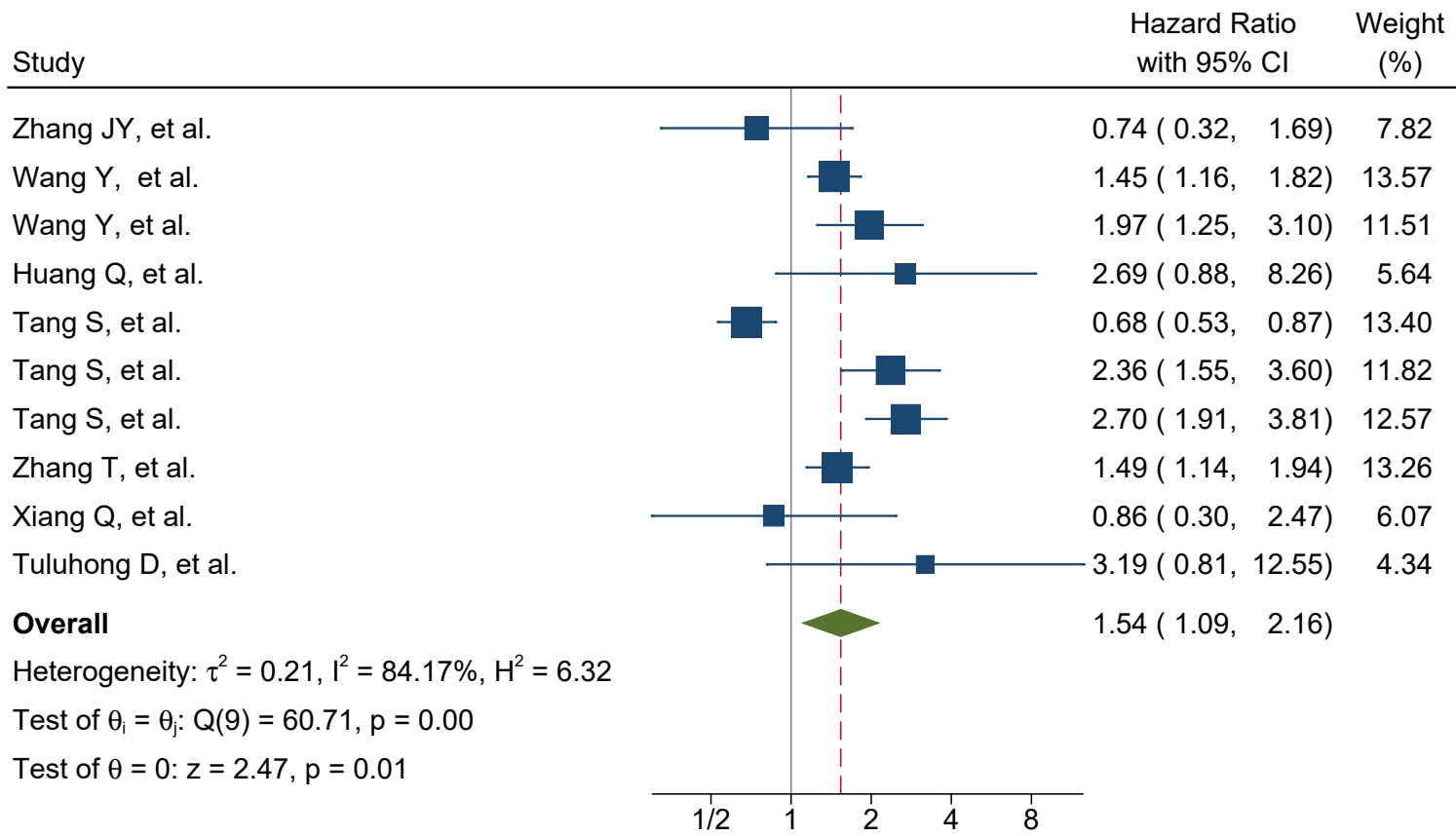

Random-effects REML model

Supplement: Supplementary file 1 [file DataSheet1.ZIP › Supplementary Material, Fig 3c.pdf]

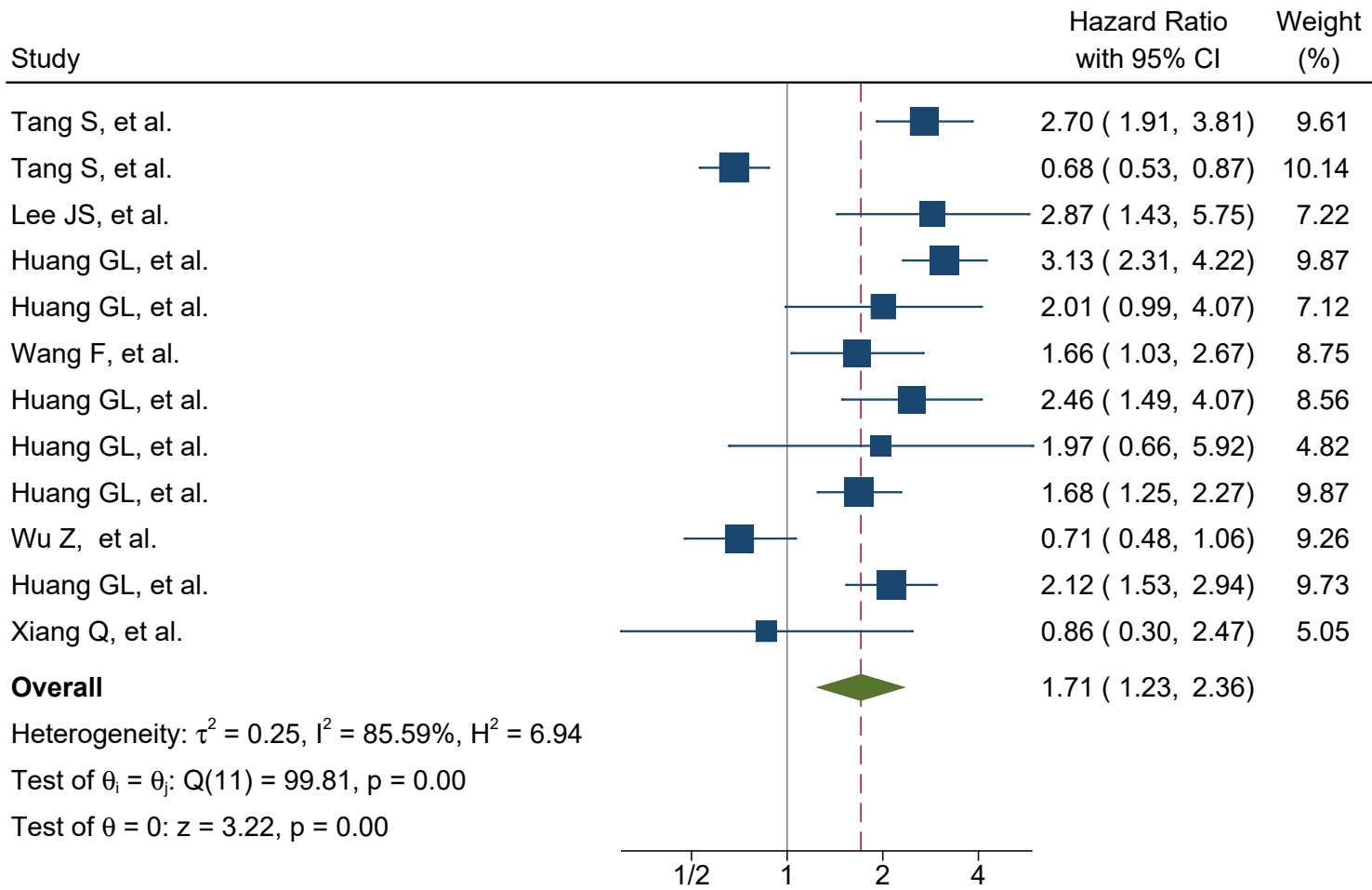

Random-effects REML model

Supplement: Supplementary file 1 [file DataSheet1.ZIP › Supplementary Material, Fig 3d.pdf]

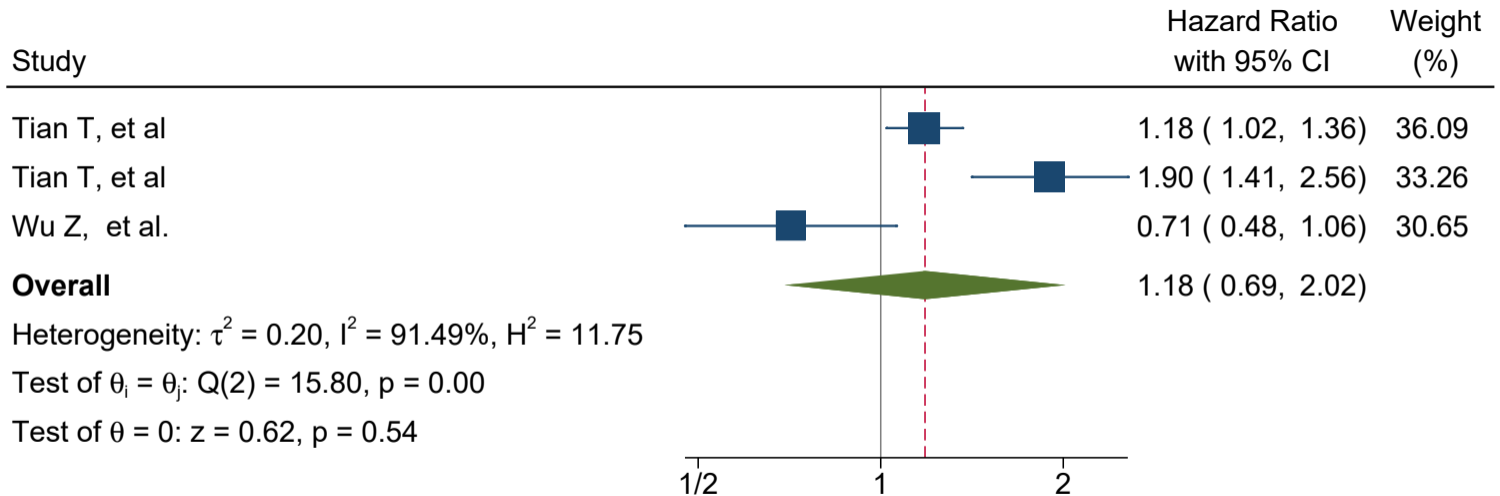

Random-effects REML model

Supplement: Supplementary file 1 [file DataSheet1.ZIP › Supplementary Material, Fig 3e.pdf]

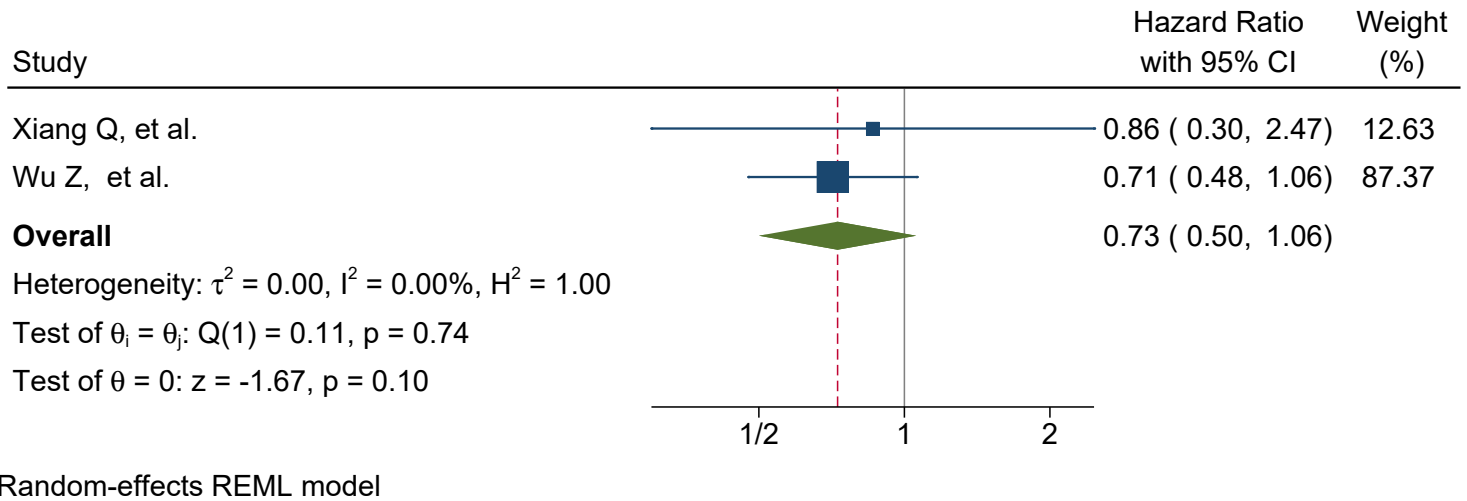

Supplement: Supplementary file 1 [file DataSheet1.ZIP › Supplementary Material, Fig 3f.pdf]

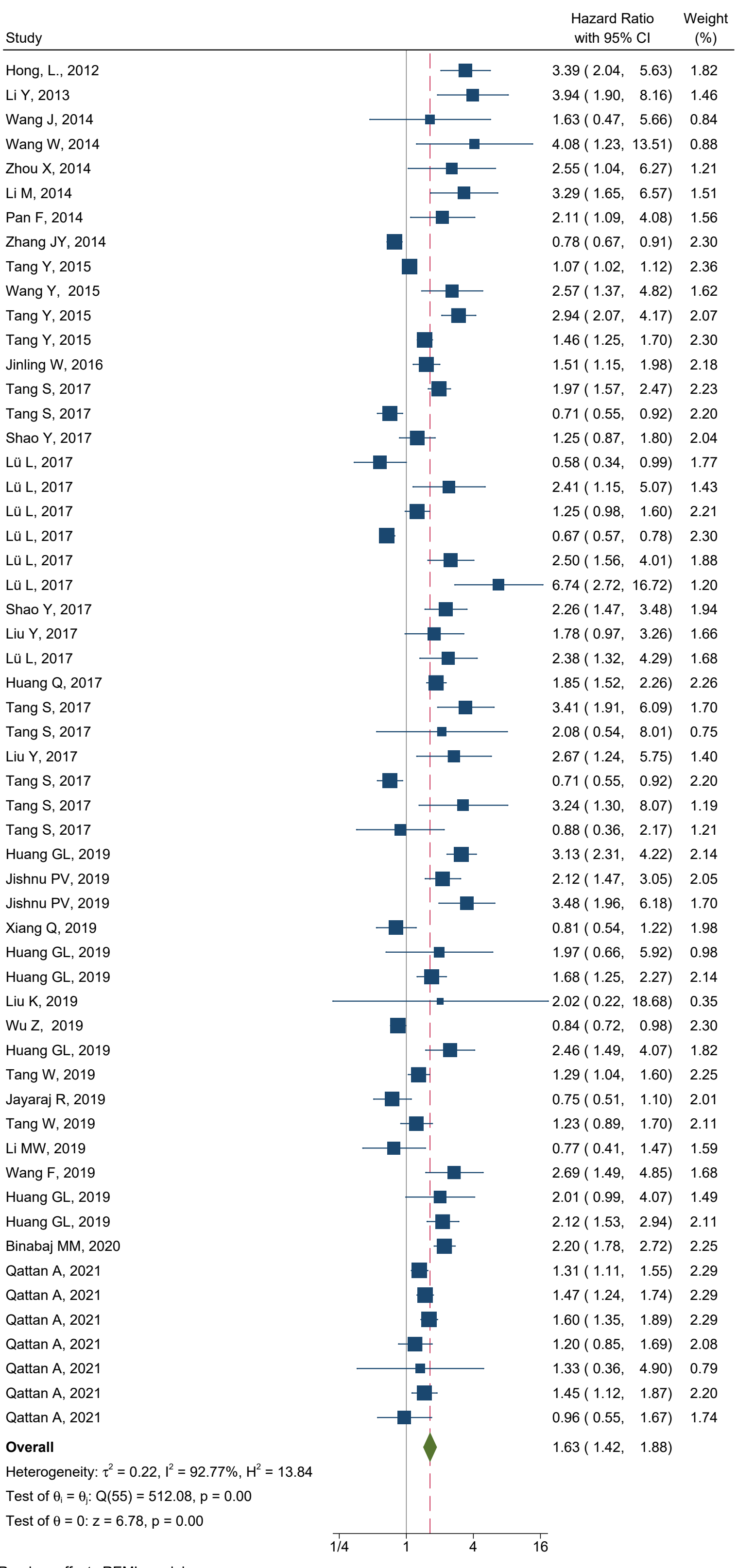

Supplement: Supplementary file 1 [file DataSheet1.ZIP › Supplementary Material, Fig 4a.pdf]

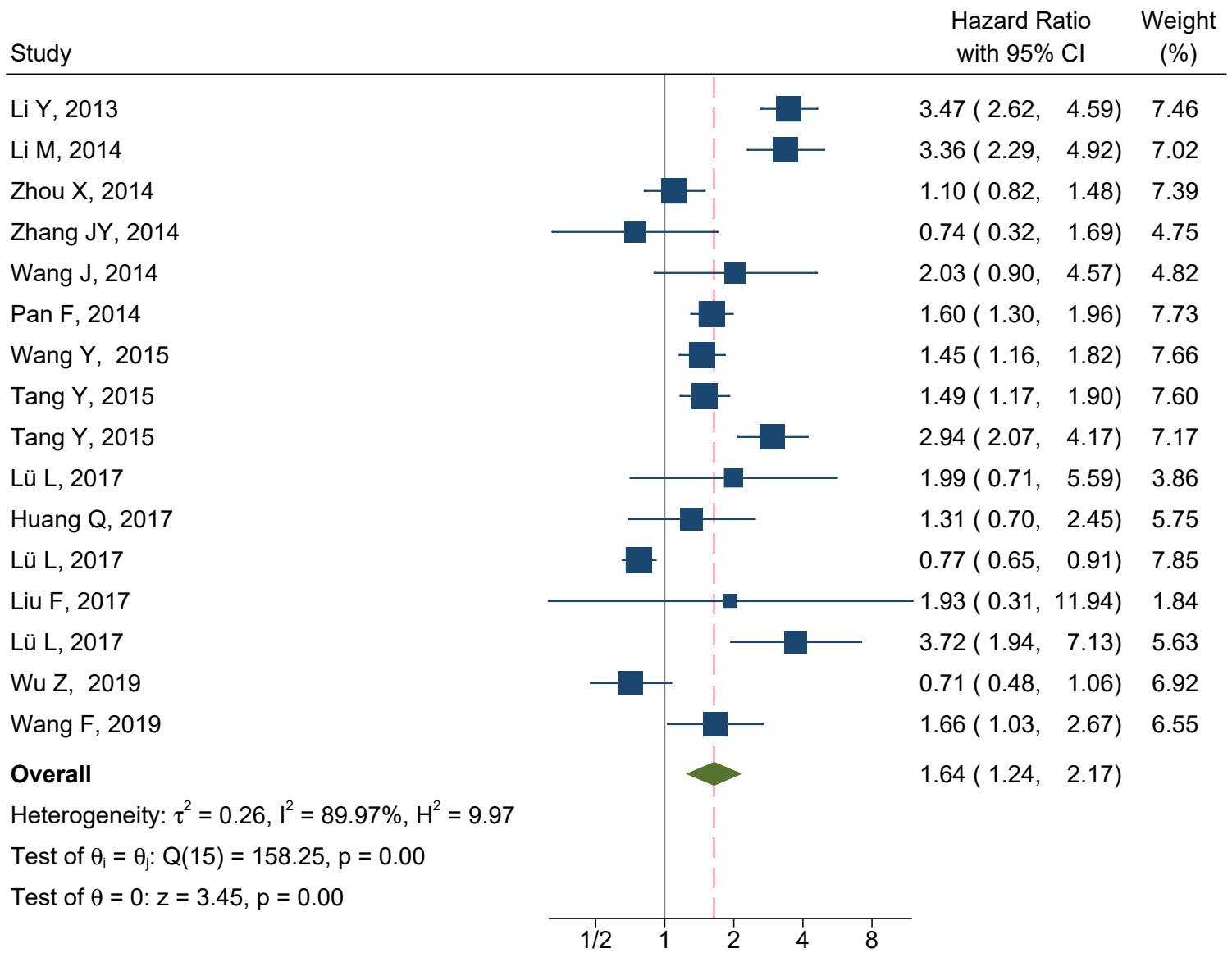

Random-effects REML model

Supplement: Supplementary file 1 [file DataSheet1.ZIP › Supplementary Material, Fig 4b.pdf]

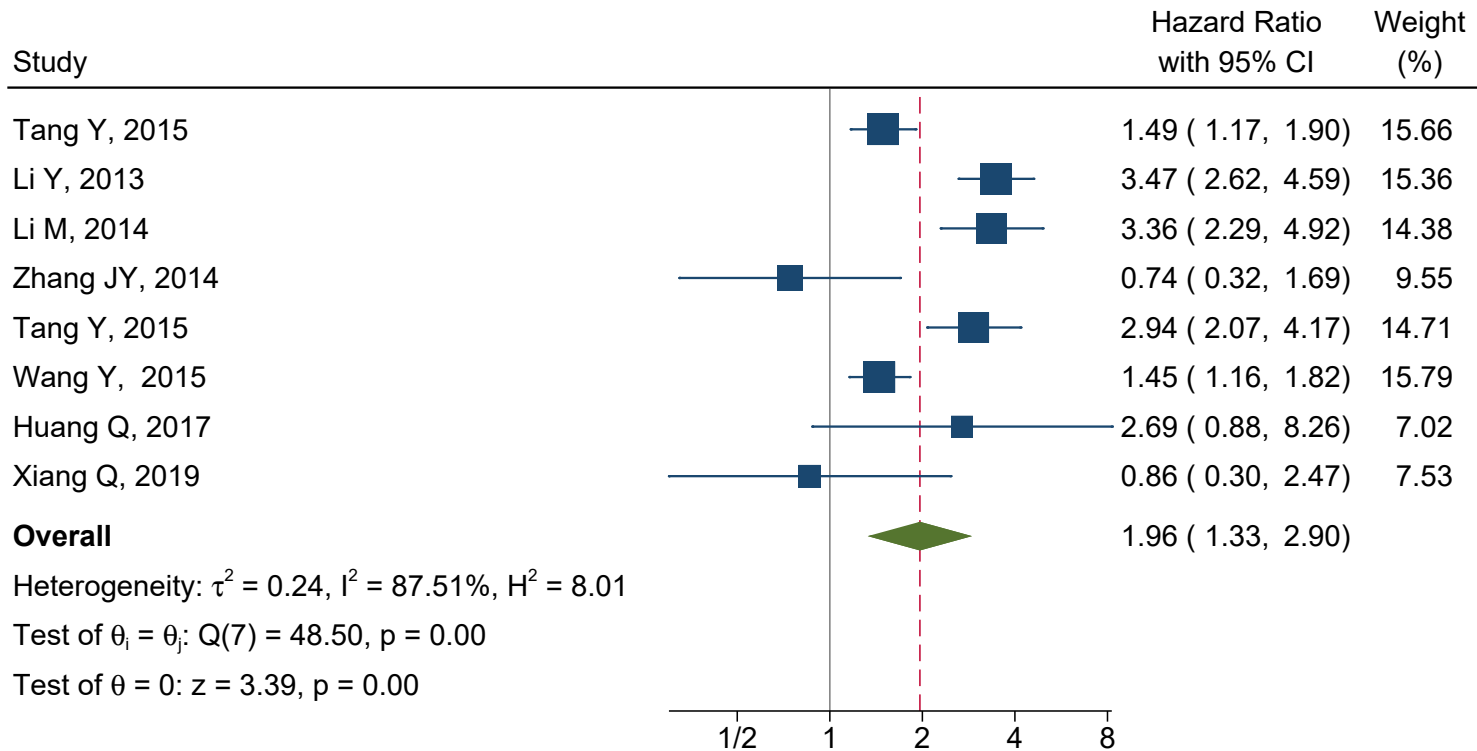

Random-effects REML model

Supplement: Supplementary file 1 [file DataSheet1.ZIP › Supplementary Material, Fig 4c.pdf]

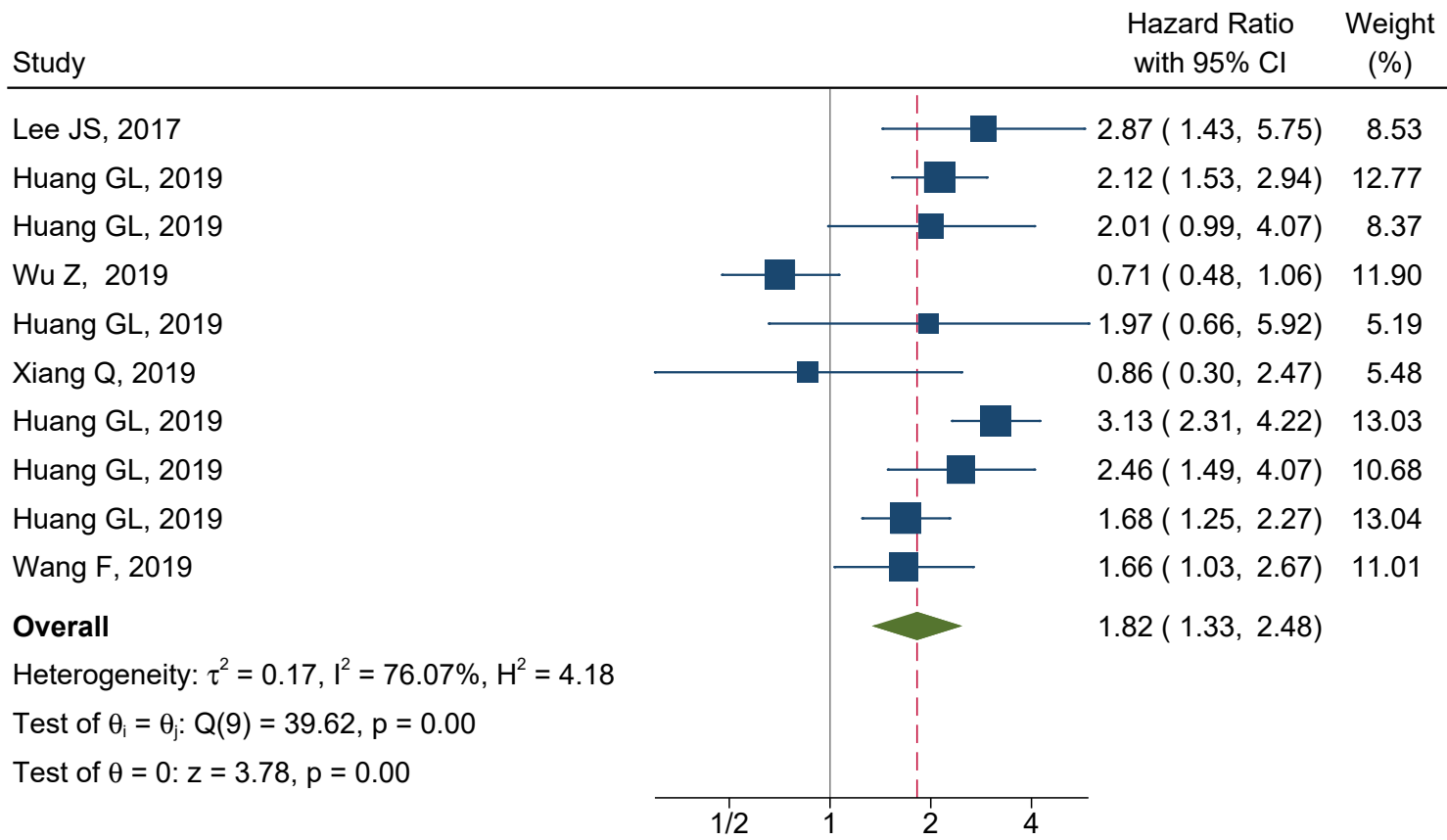

Random-effects REML model

Supplement: Supplementary file 1 [file DataSheet1.ZIP › Supplementary Material, Fig 4d.pdf]

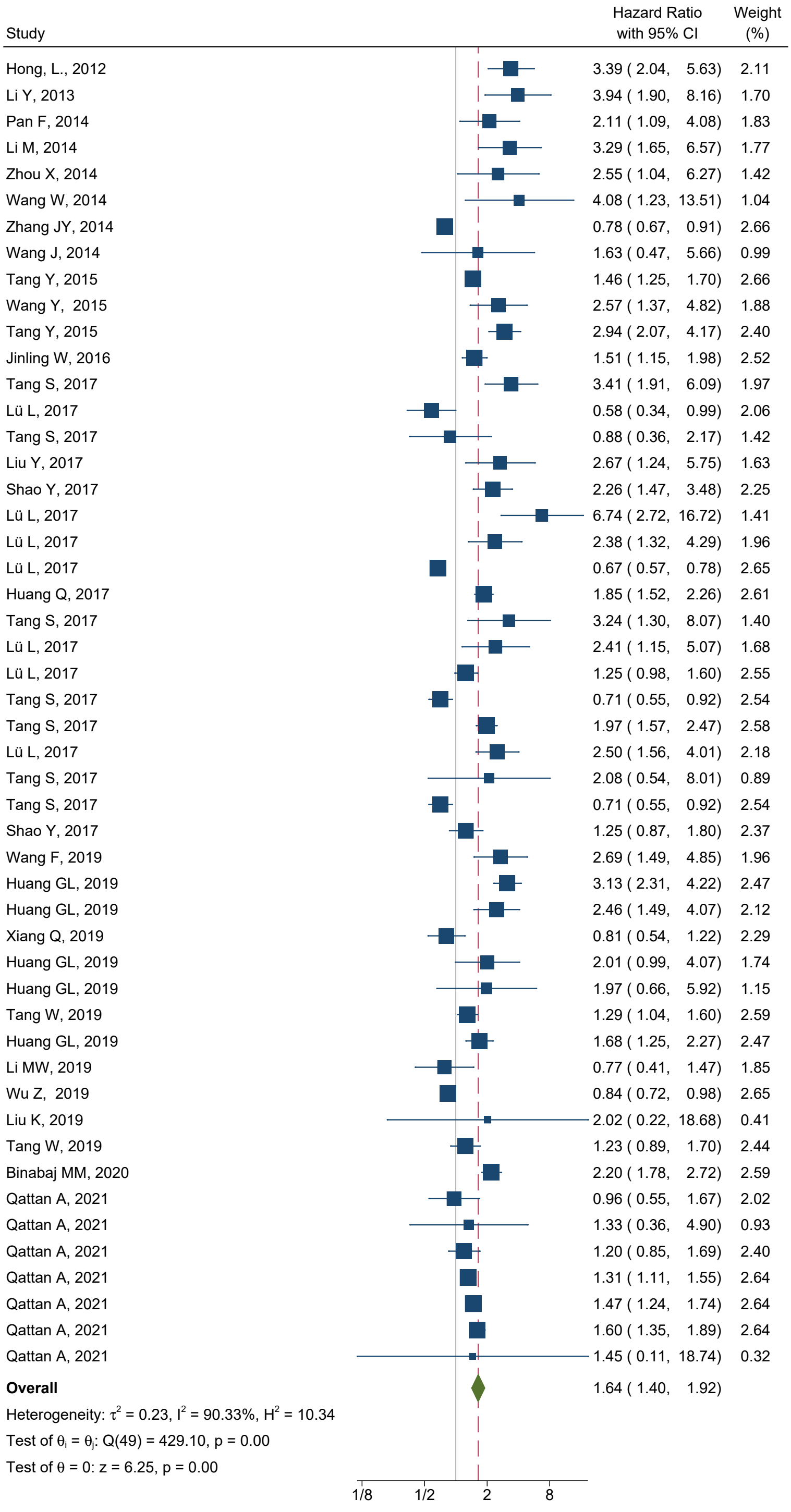

Supplement: Supplementary file 1 [file DataSheet1.ZIP › Supplementary Material, Fig 4e.pdf]

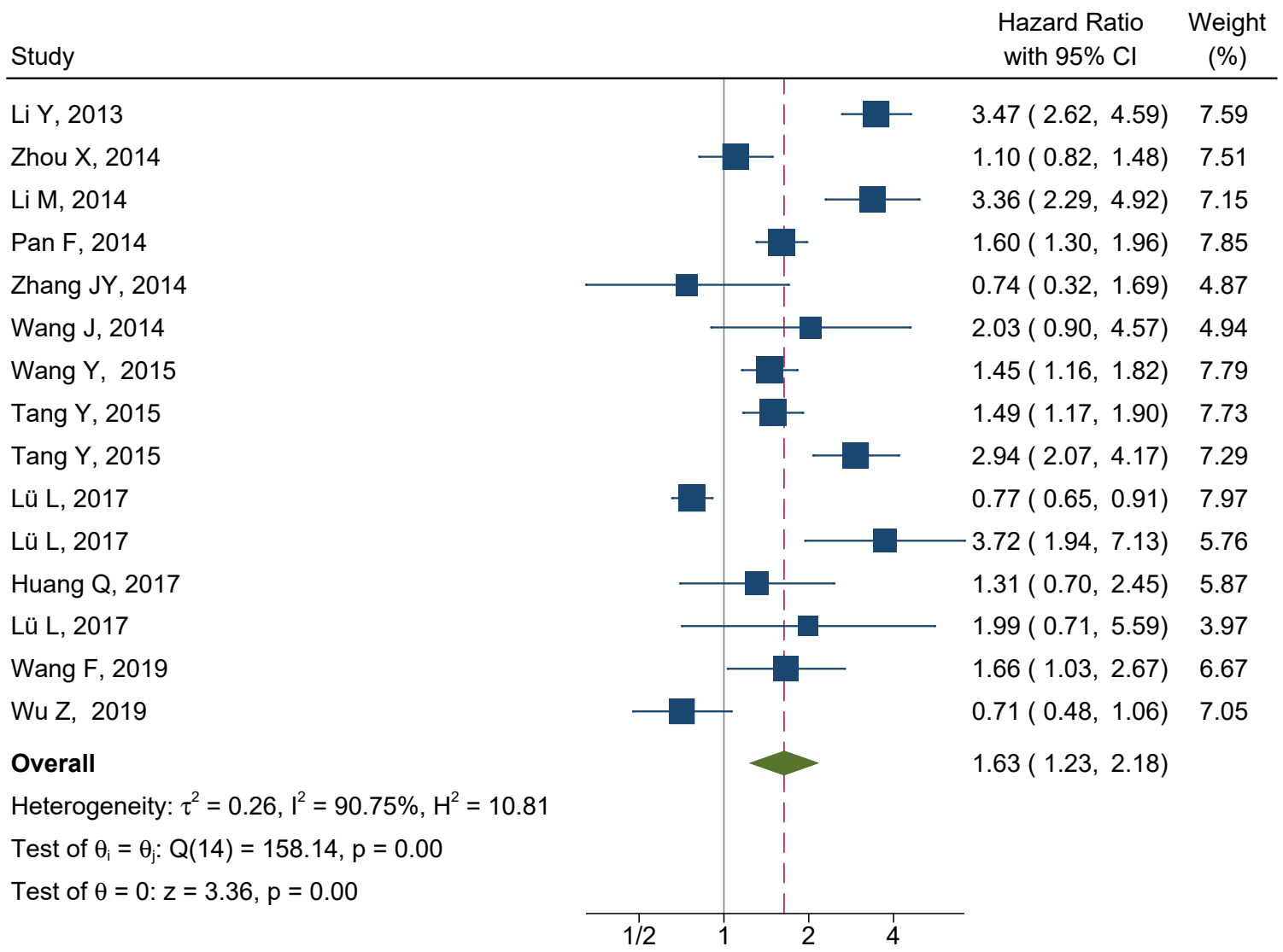

Random-effects REML model

Supplement: Supplementary file 1 [file DataSheet1.ZIP › Supplementary Material, Fig 4f.pdf]

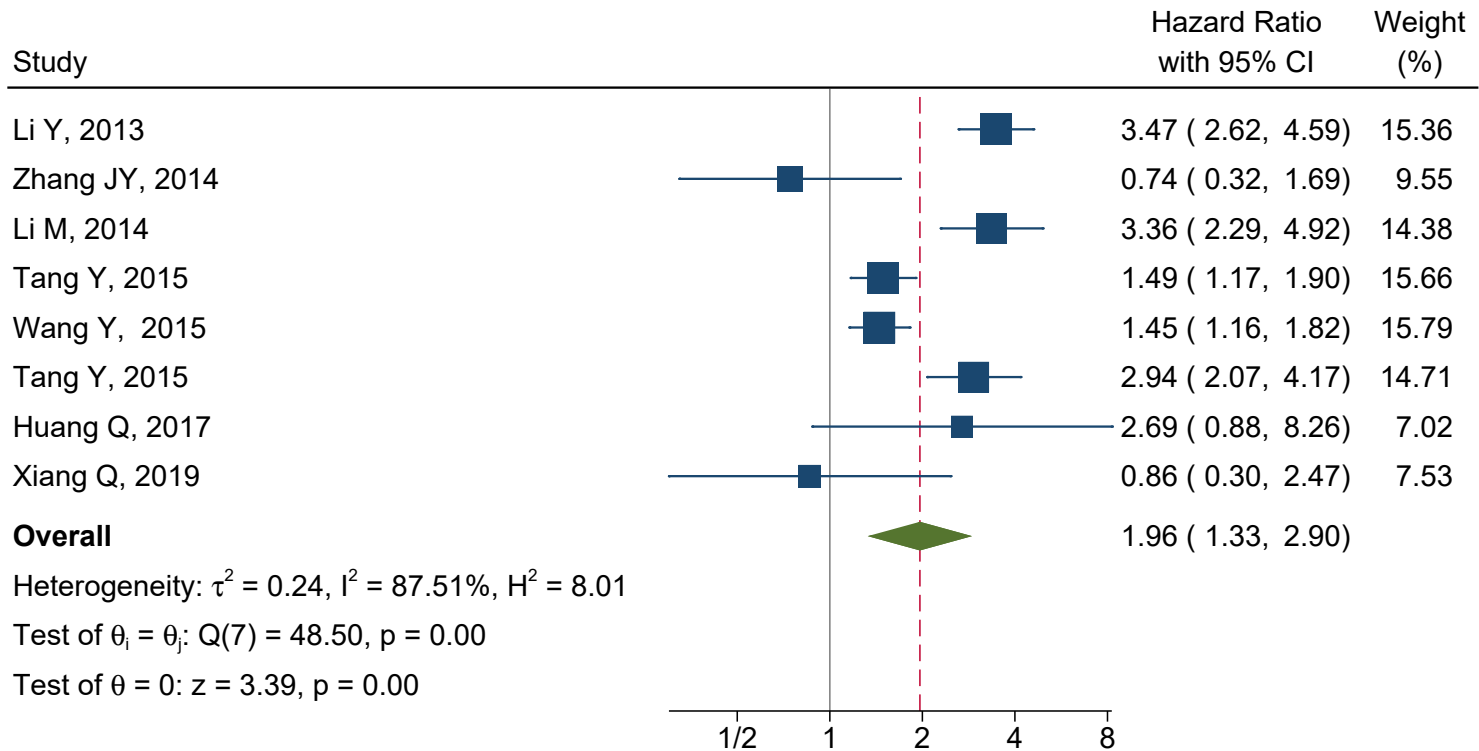

Random-effects REML model

Supplement: Supplementary file 1 [file DataSheet1.ZIP › Supplementary Material, Fig 4g.pdf]

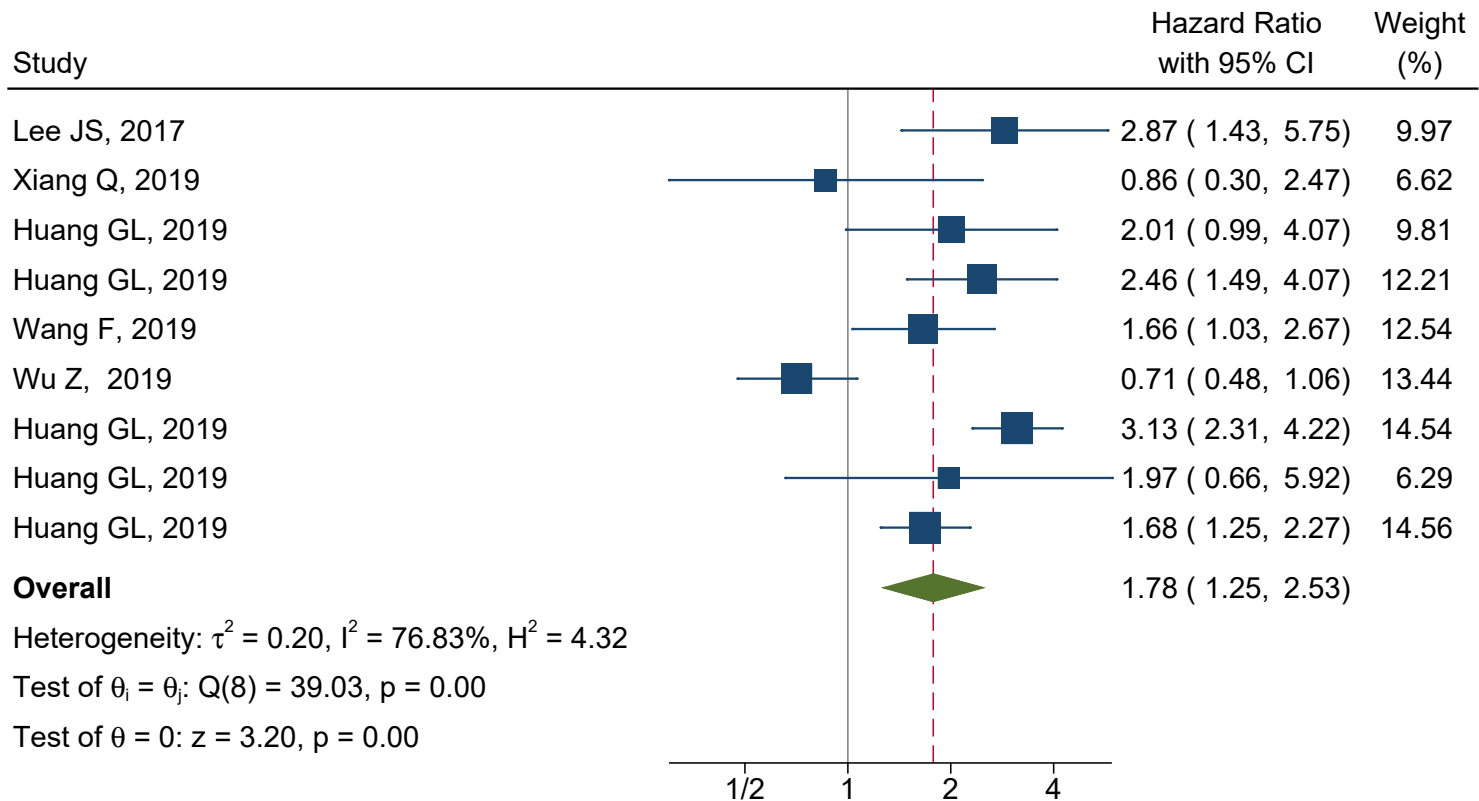

Supplement: Supplementary file 1 [file DataSheet1.ZIP › Supplementary Material, Fig 4h.pdf]

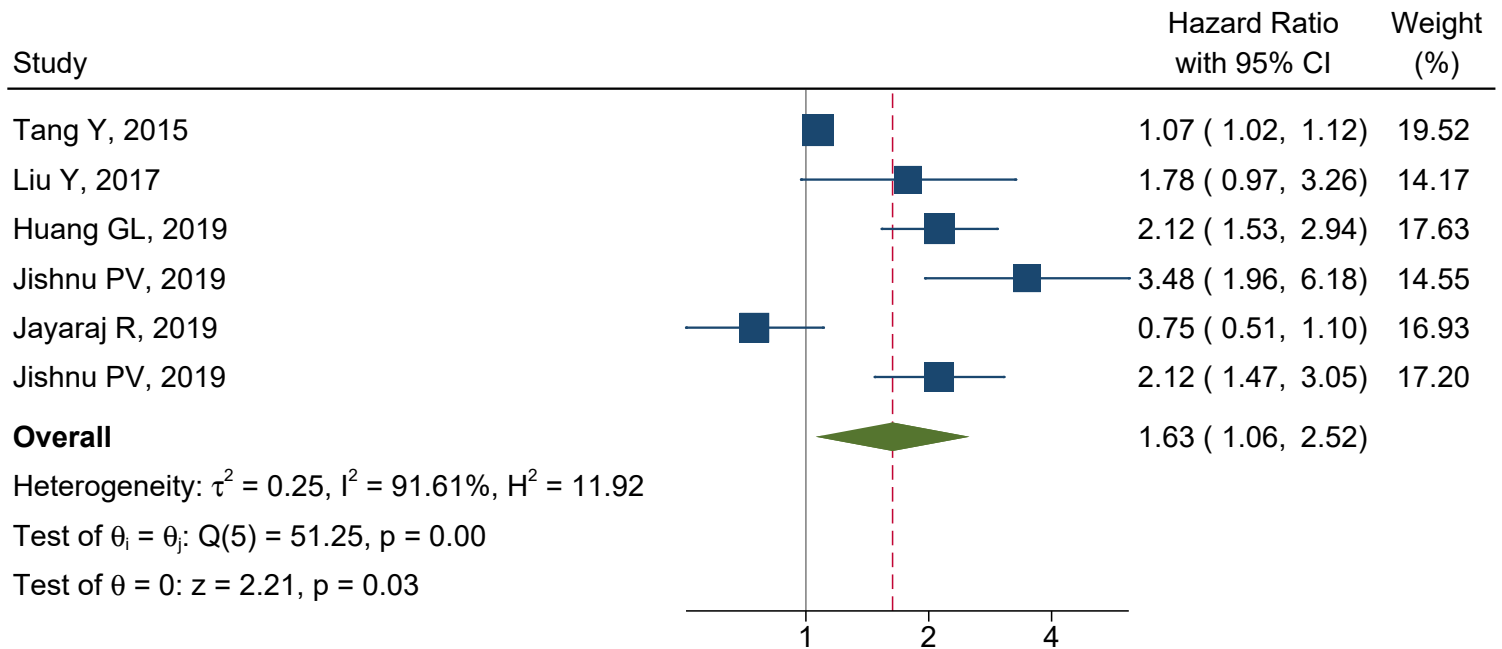

Random-effects REML model

Supplement: Supplementary file 1 [file DataSheet1.ZIP › Supplementary Material, Fig 4i.pdf]

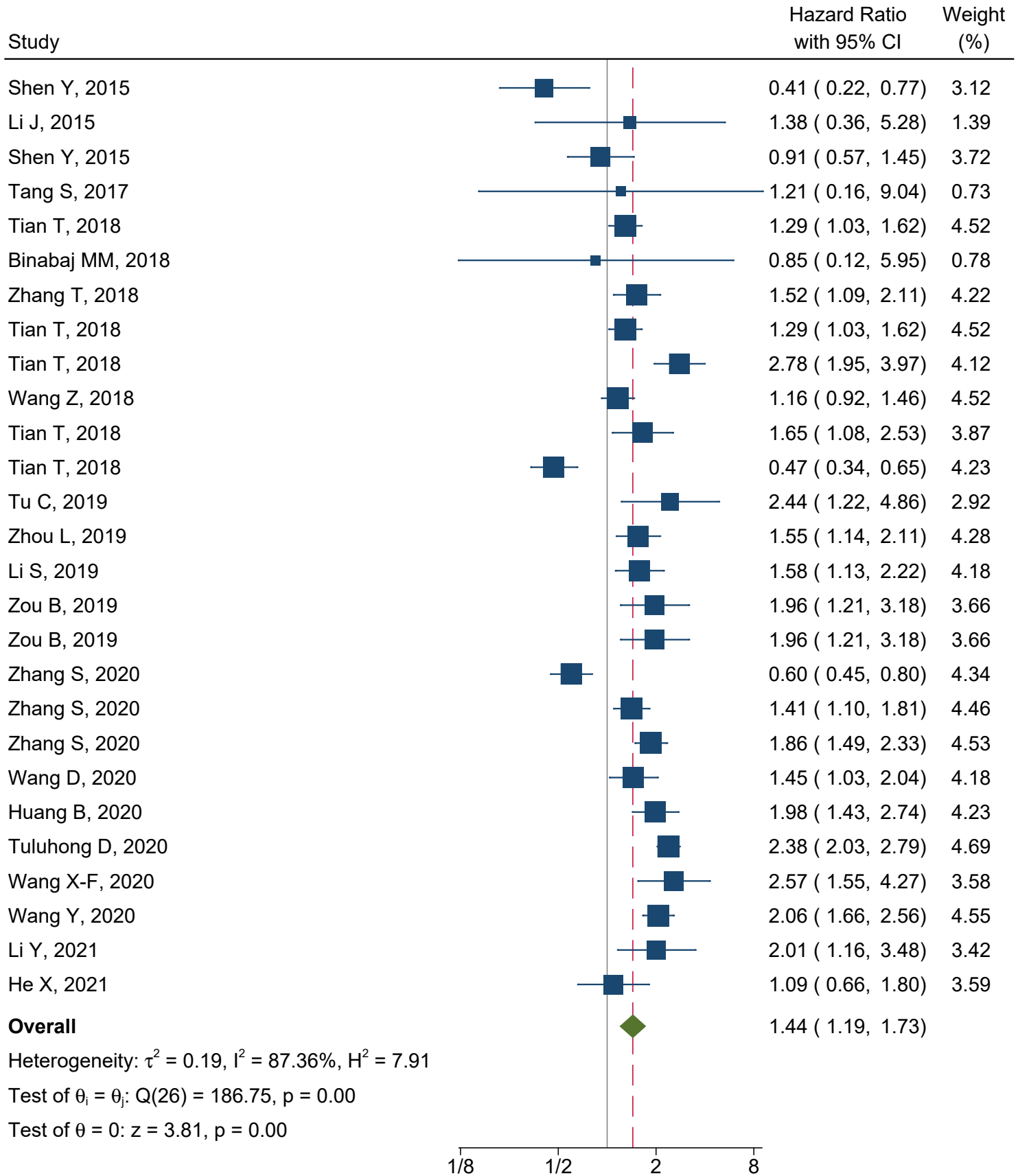

Random-effects REML model

Supplement: Supplementary file 1 [file DataSheet1.ZIP › Supplementary Material, Fig 5a.pdf]

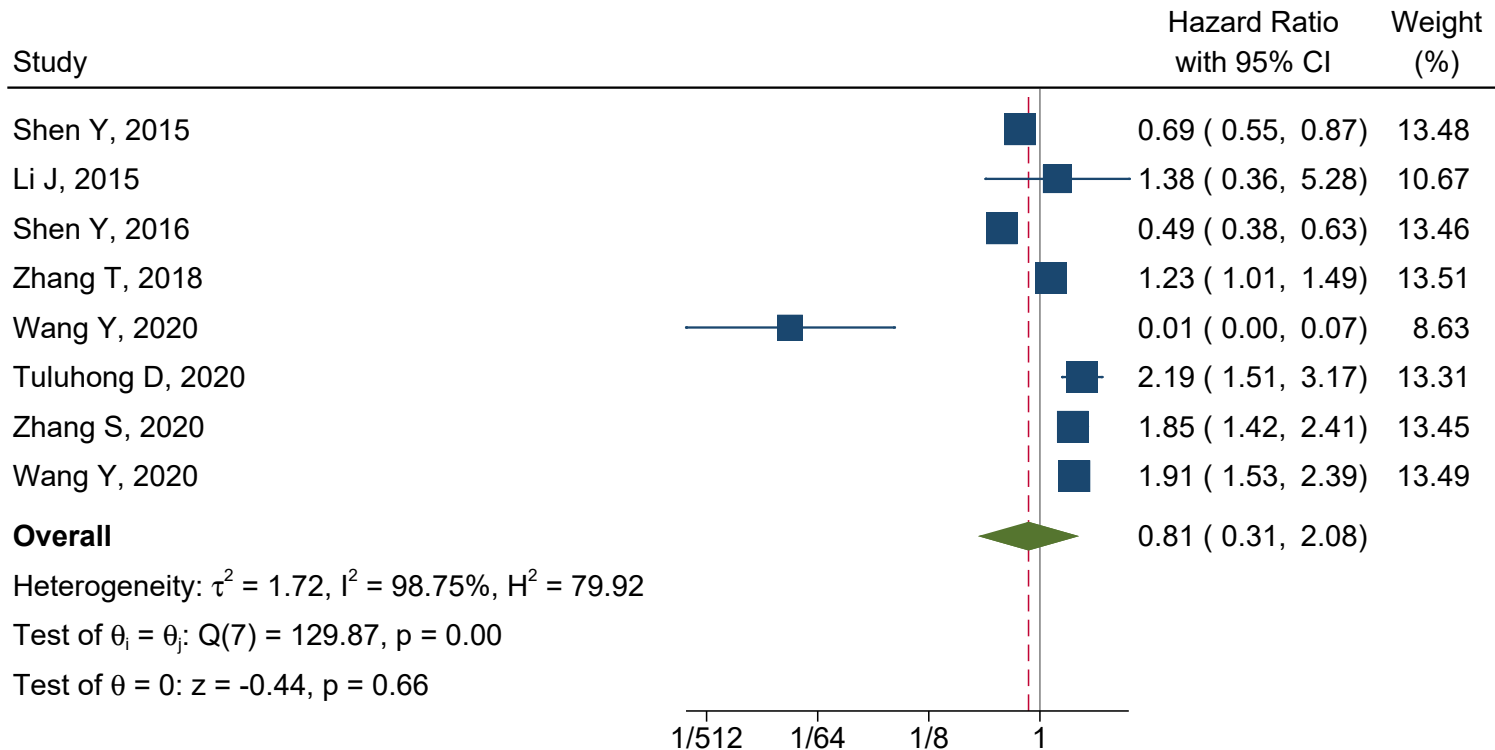

Random-effects REML model

Supplement: Supplementary file 1 [file DataSheet1.ZIP › Supplementary Material, Fig 5b.pdf]

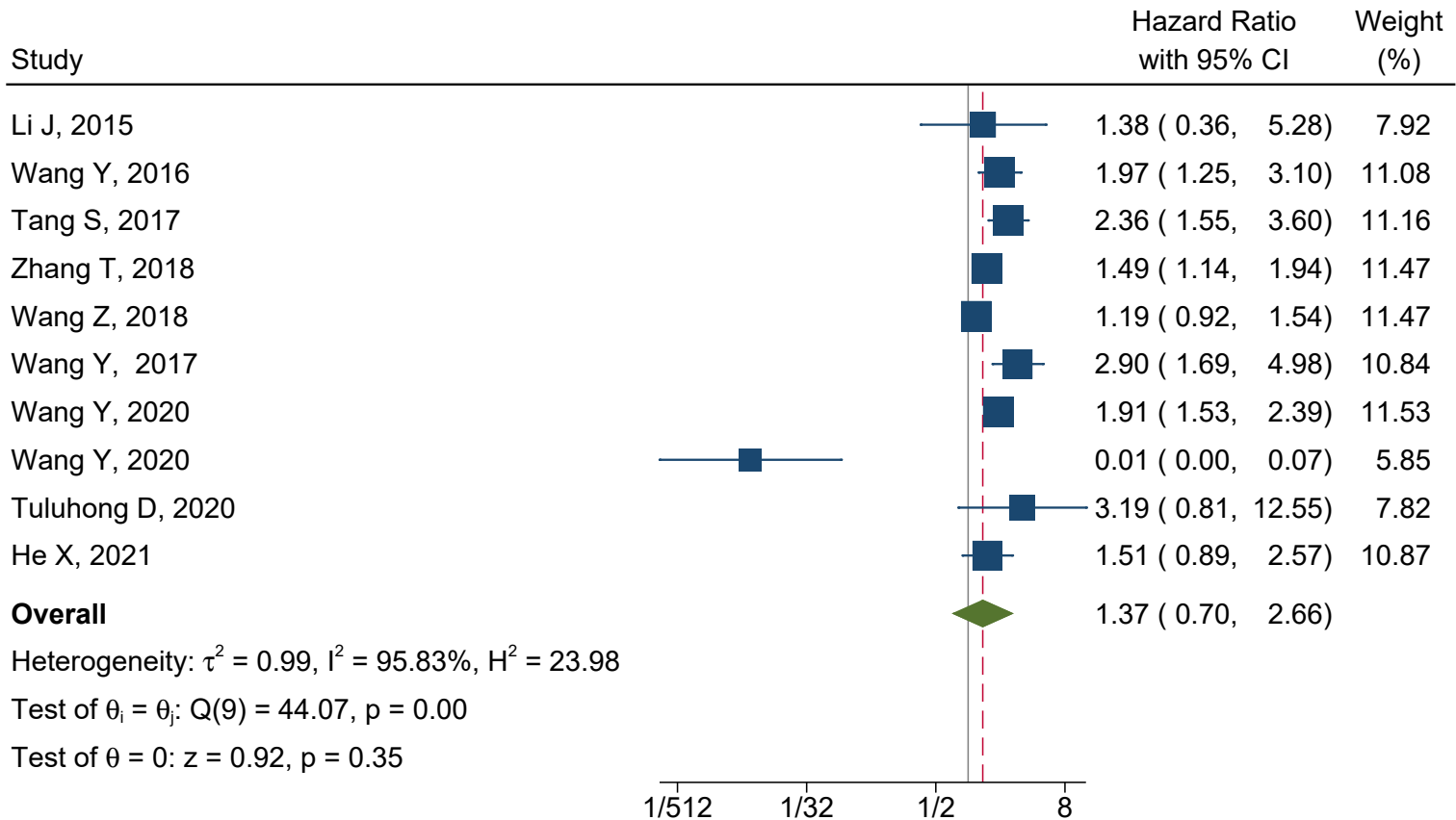

Supplement: Supplementary file 1 [file DataSheet1.ZIP › Supplementary Material, Fig 5c.pdf]

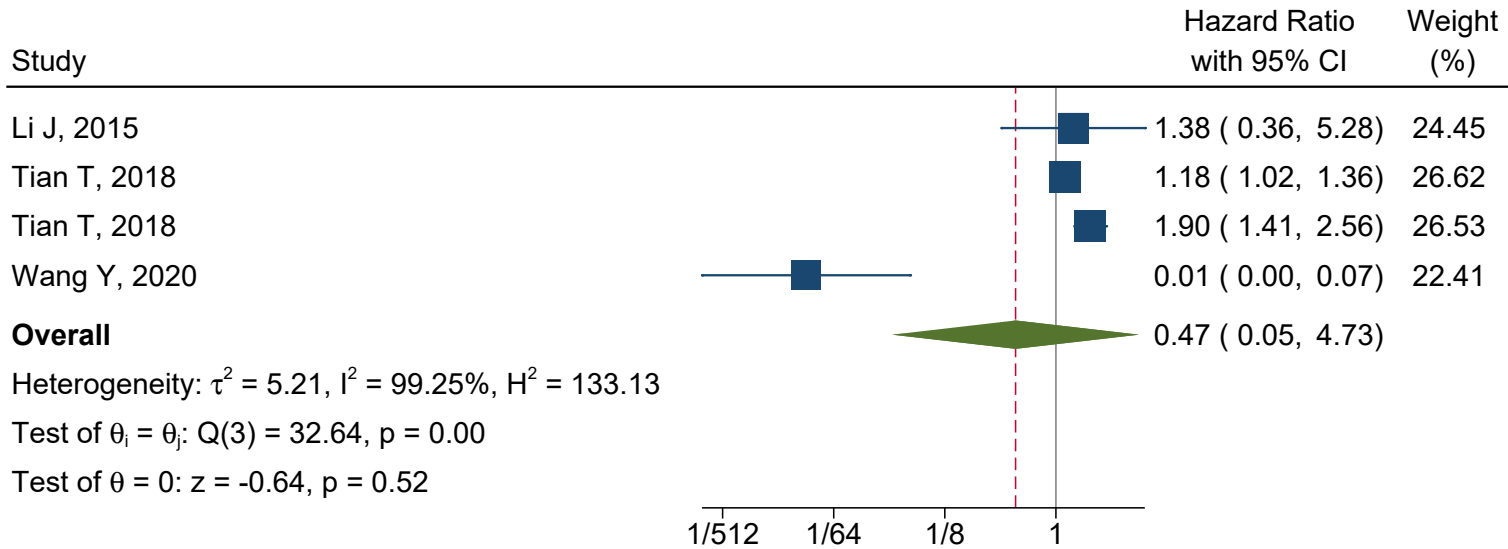

Random-effects REML model

Supplement: Supplementary file 1 [file DataSheet1.ZIP › Supplementary Material, Fig 5d.pdf]

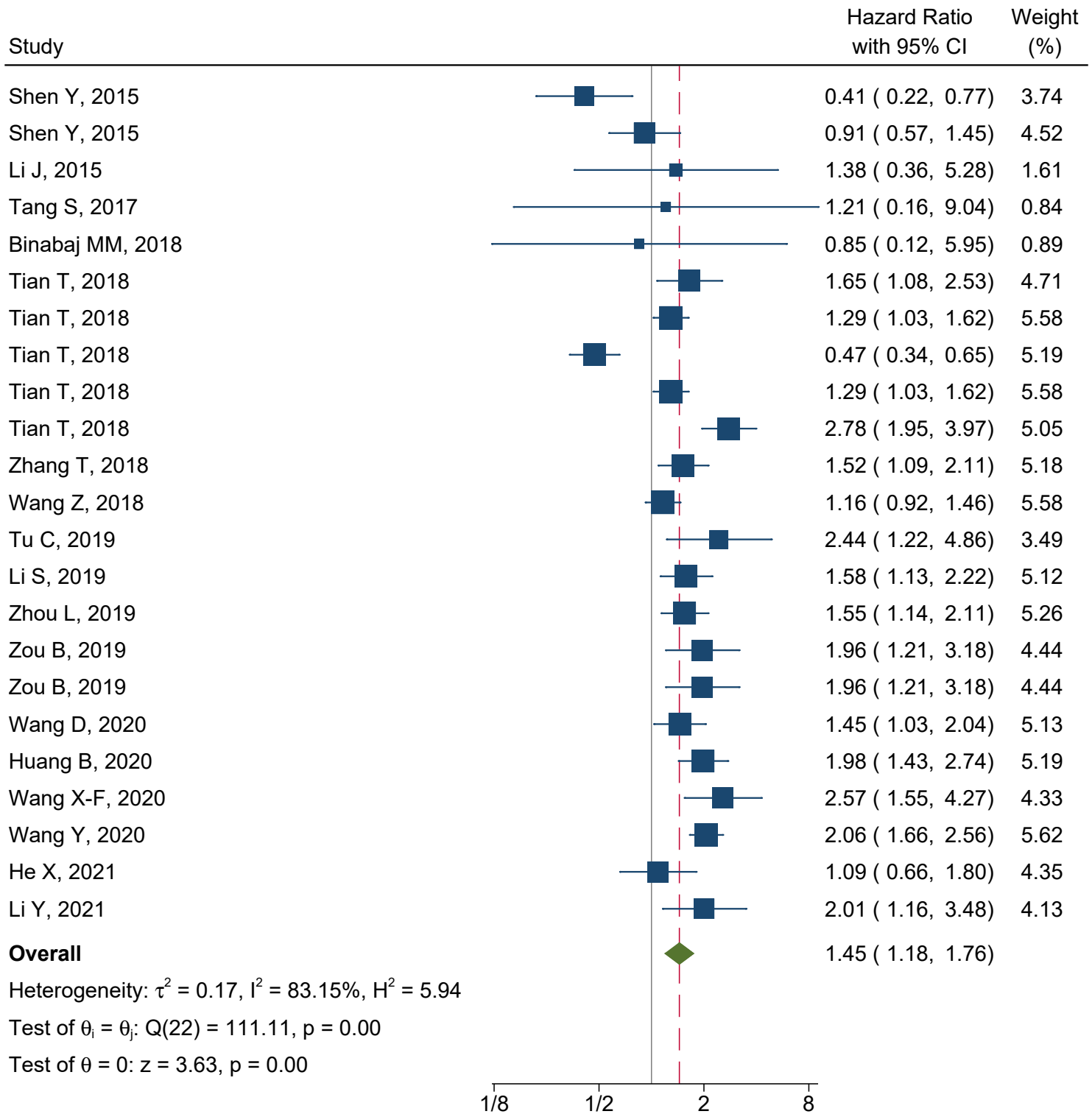

Random-effects REML model

Supplement: Supplementary file 1 [file DataSheet1.ZIP › Supplementary Material, Fig 5e.pdf]

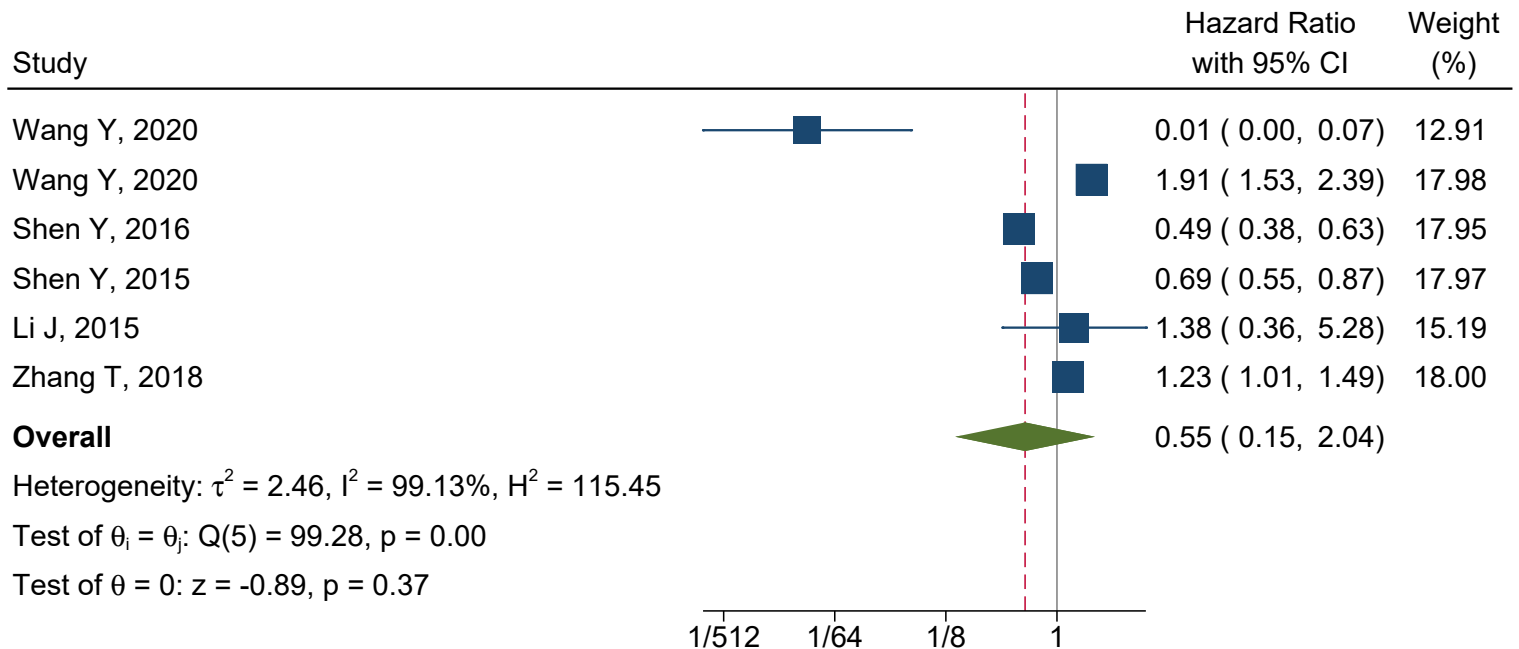

Random-effects REML model

Supplement: Supplementary file 1 [file DataSheet1.ZIP › Supplementary Material, Fig 5f.pdf]

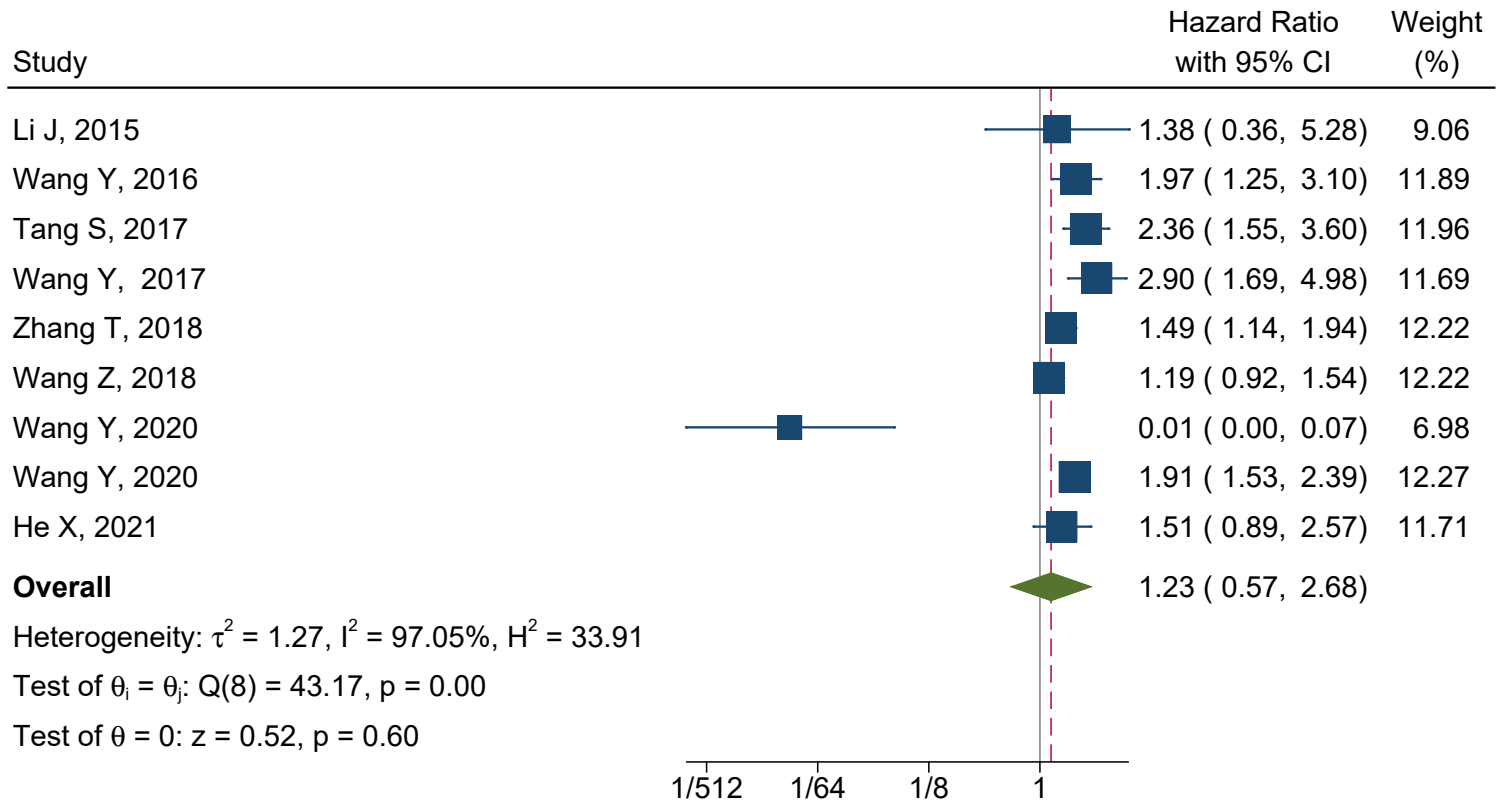

Supplement: Supplementary file 1 [file DataSheet1.ZIP › Supplementary Material, Fig 5g.pdf]

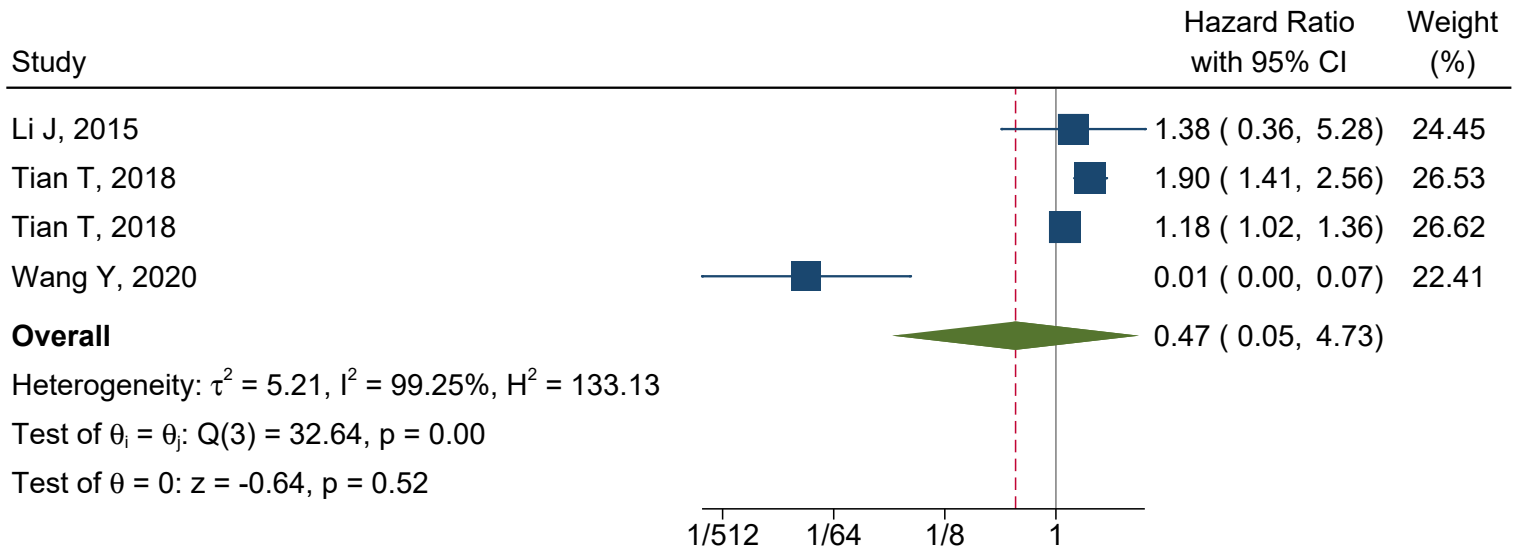

Random-effects REML model

Supplement: Supplementary file 1 [file DataSheet1.ZIP › Supplementary Material, Fig 5h.pdf]

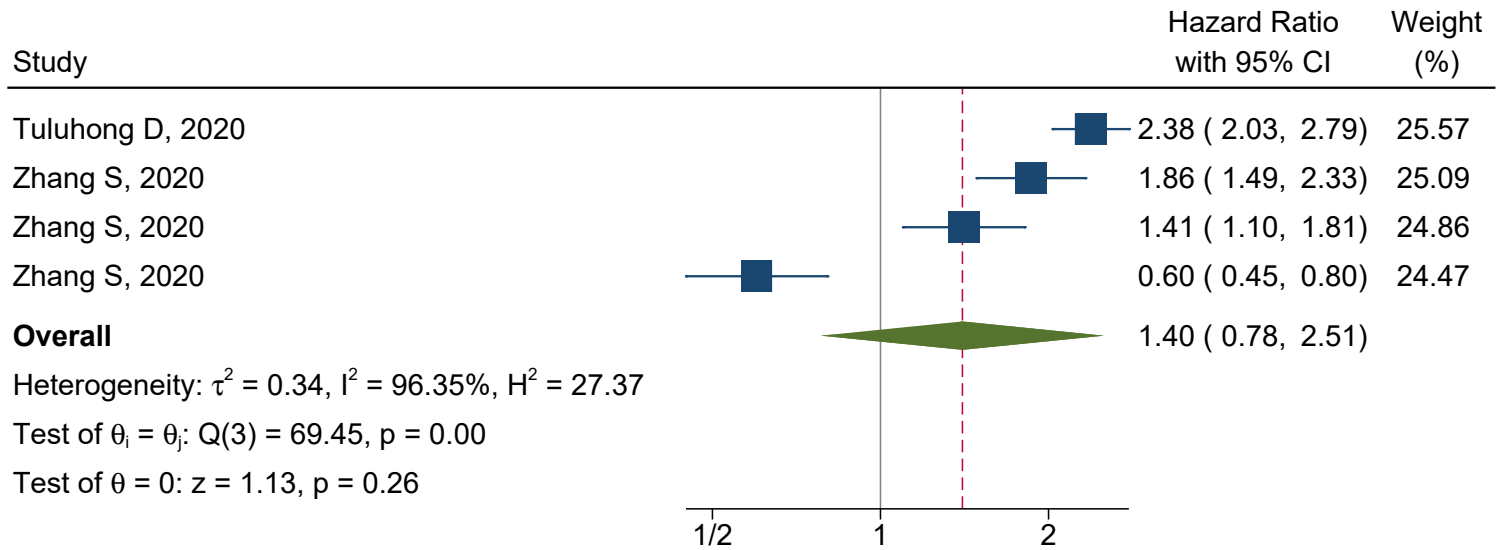

Random-effects REML model

Supplement: Supplementary file 1 [file DataSheet1.ZIP › Supplementary Material, Fig 5i.pdf]

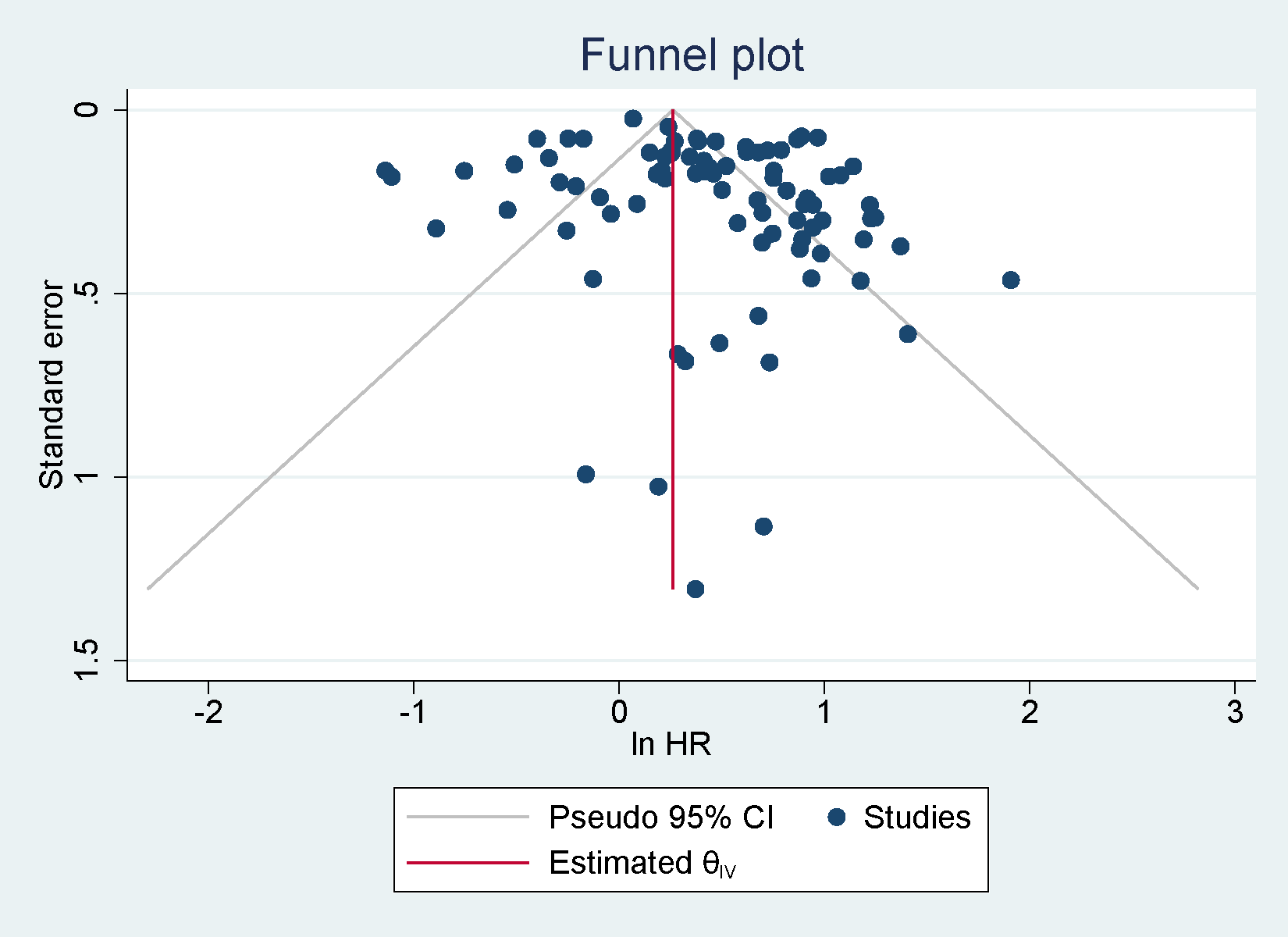

Supplement: Supplementary file 1 [file DataSheet1.ZIP › Supplementary Material, Fig 6.tif]

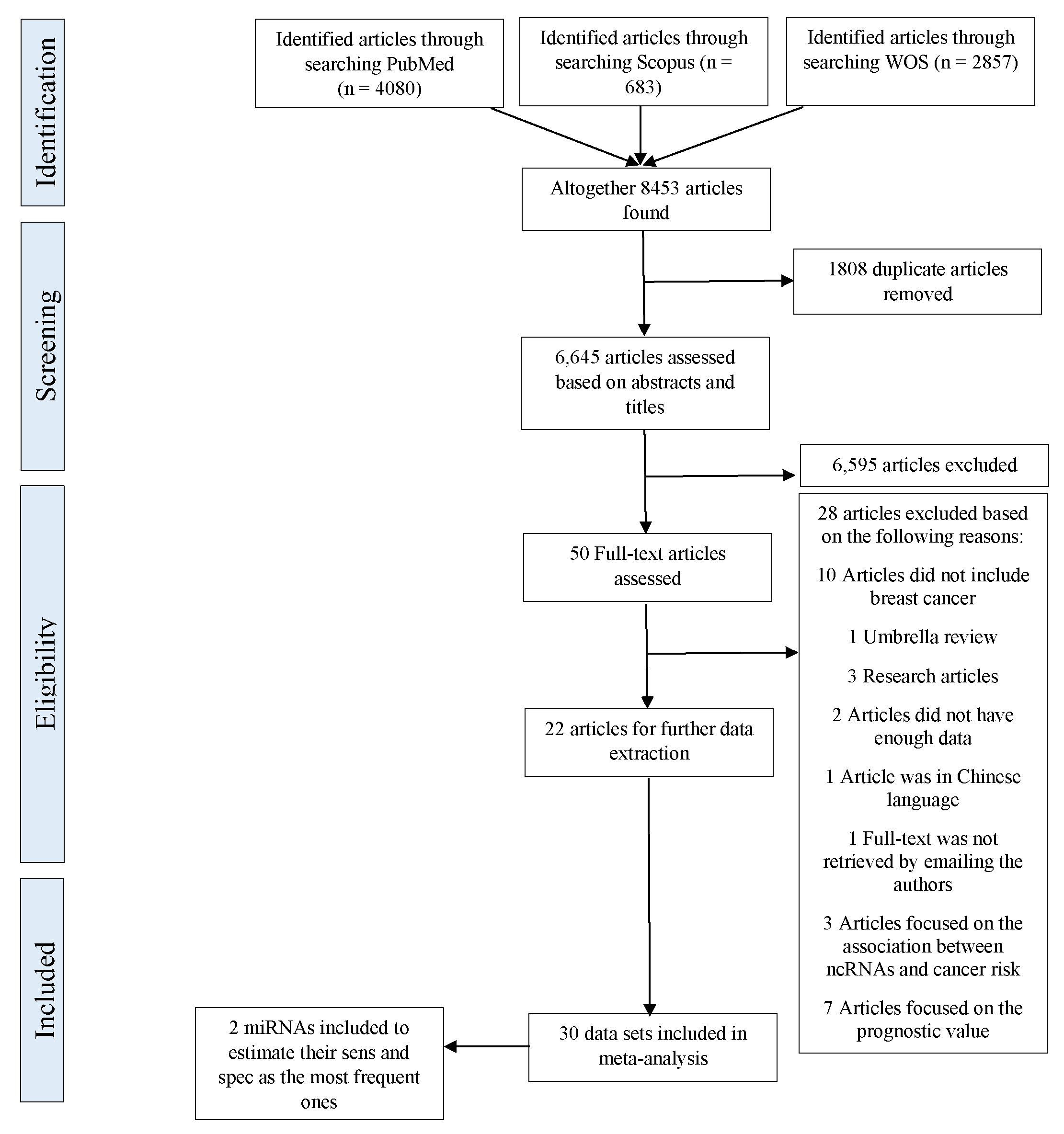

Supplement: Supplementary file 1 [file DataSheet1.ZIP › Supplementary Material, Fig 7.tif]

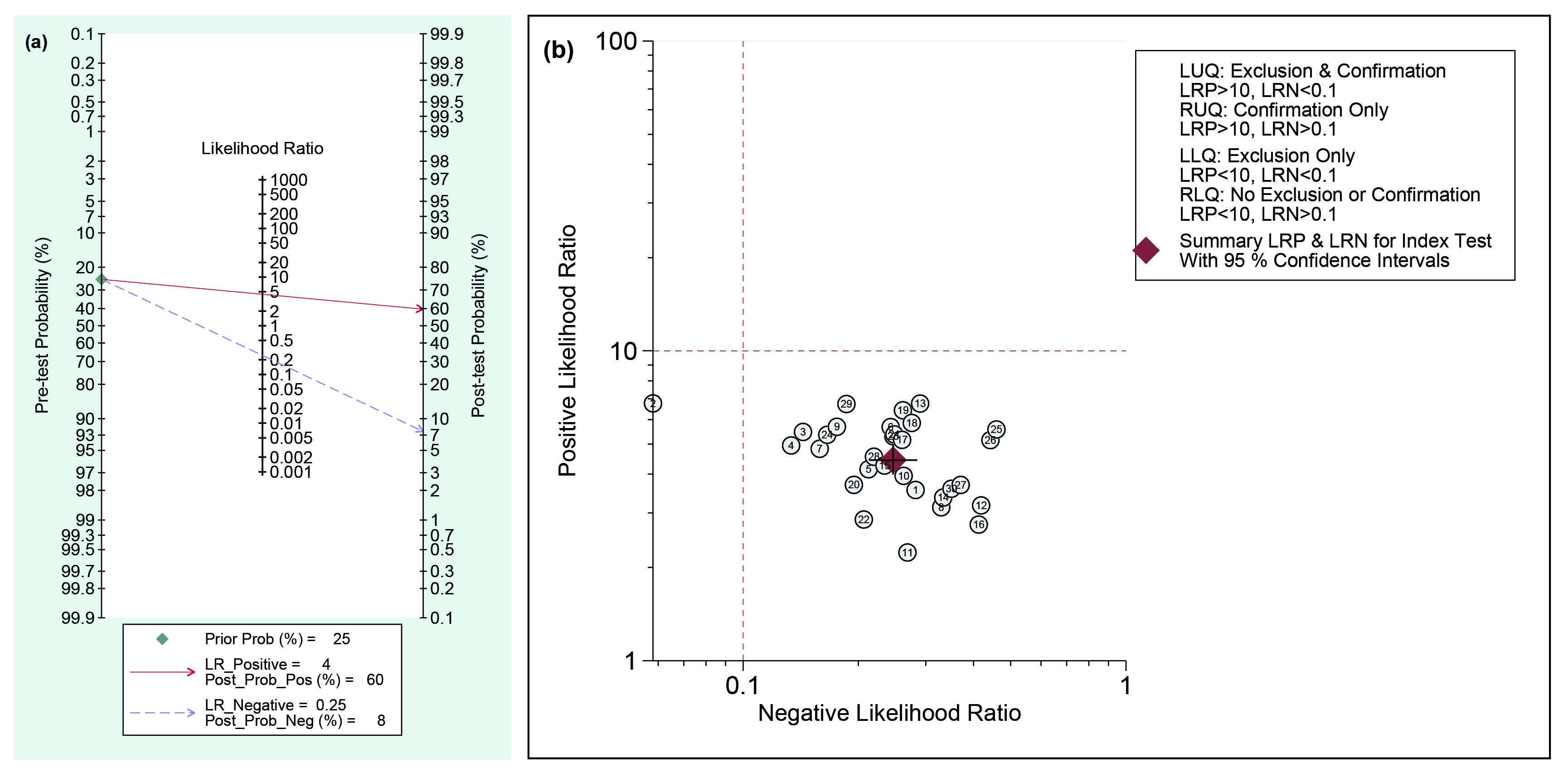

Supplement: Supplementary file 1 [file DataSheet1.ZIP › Supplementary Material, Fig 8.tif]

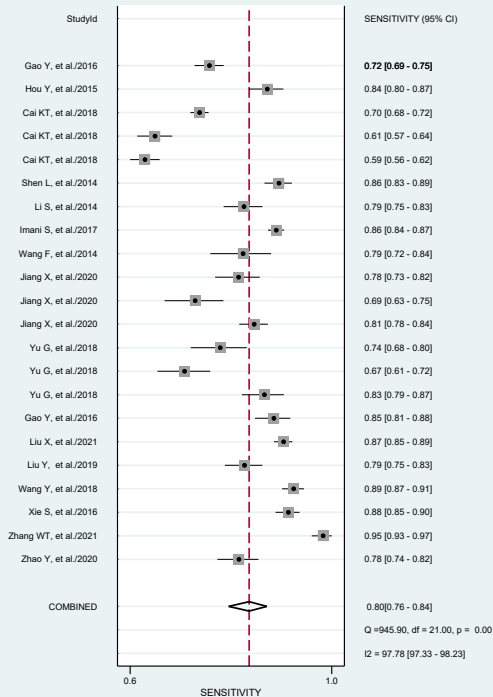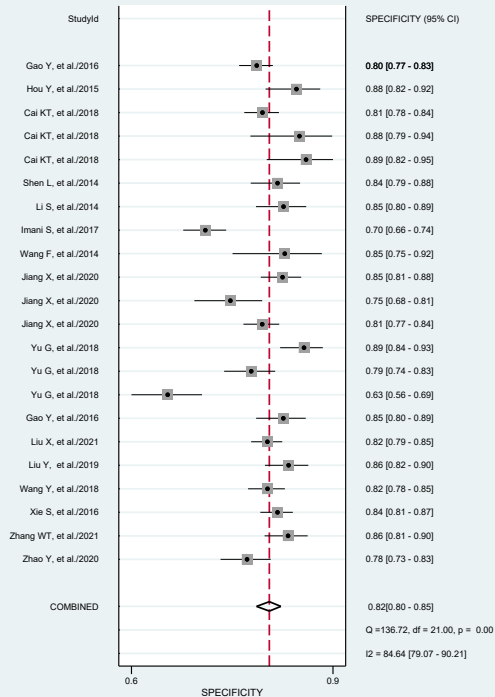

Supplement: Supplementary file 1 [file DataSheet1.ZIP › Supplementary Material, Fig 9a.pdf]

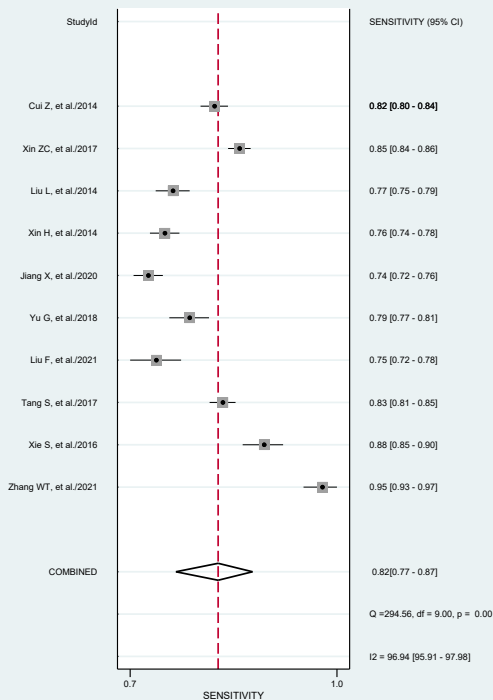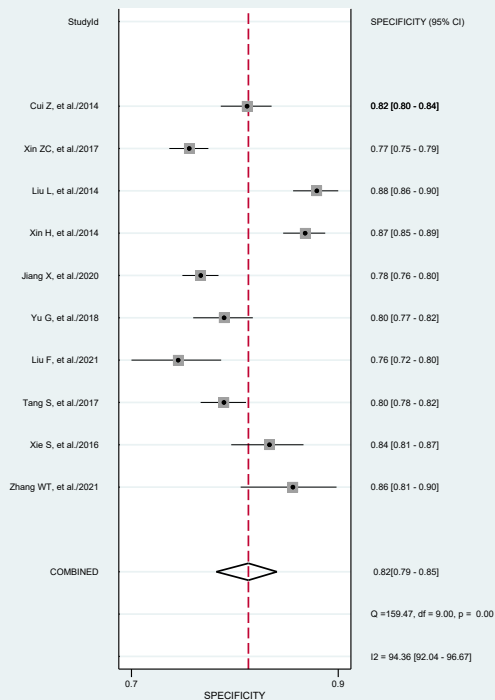

Supplement: Supplementary file 1 [file DataSheet1.ZIP › Supplementary Material, Fig 9b.pdf]
