# Supplementary material for: Prognostic and diagnostic values of non-coding RNAs as biomarkers for breast cancer: An umbrella review and pan-cancer analysis
Source: Front Mol Biosci. 2023 Jan 16;10:1096524. doi: 10.3389/fmolb.2023.1096524 (PMC9885171; doi:10.3389/fmolb.2023.1096524)
Supplement: Supplementary file 2 [file DataSheet2.ZIP › Supplementary Material, Table 1.docx]

**Supplementary Material, Table 1.** Search strategy details in PubMed.

| **PubMed’s search strategy** |
| --- |
| ((((((breast cancer[MeSH Terms]) OR (breast cancer[Title/Abstract])) OR (breast[Title/Abstract])) OR (breast tumor[Title/Abstract])) OR (breast carcinoma[Title/Abstract])) AND (((((((((((((((((miRNA[Title/Abstract]) OR (mir[Title/Abstract])) OR (miRNAs[Title/Abstract])) OR (microRNA[Title/Abstract])) OR (microRNAs[Title/Abstract])) OR (mirna[MeSH Terms])) OR (microrna[MeSH Terms])) OR (micrornas[MeSH Terms])) OR (lncRNA[Title/Abstract])) OR (lncRNAs[Title/Abstract])) OR (long non-coding RNA[Title/Abstract])) OR (long non-coding RNAs[Title/Abstract])) OR (non coding rna[MeSH Terms])) OR (rna, non coding[MeSH Terms])) OR (non coding rna, small[MeSH Terms])) OR (rna, small non coding[MeSH Terms])) OR (small non coding rna[MeSH Terms]))) AND ((((prognosis[Title/Abstract]) OR (prognosis[MeSH Terms])) OR (prognostic[Title/Abstract])) OR (prognostic value[Title/Abstract])) |
