# Supplementary material for: Prognostic and diagnostic values of non-coding RNAs as biomarkers for breast cancer: An umbrella review and pan-cancer analysis
Source: Front Mol Biosci. 2023 Jan 16;10:1096524. doi: 10.3389/fmolb.2023.1096524 (PMC9885171; doi:10.3389/fmolb.2023.1096524)
Supplement: Supplementary file 2 [file DataSheet2.ZIP › Supplementary Material, Table 10.docx]

**Supplementary Material, Table 10.** Characteristics of included articles

| **No** | **First Author** | **Search date** | **Databases** | **Cases**  **(n)** | **Controls**  **(n)** | **No. of study estimates** | **Studies Quality** | **Biomarkers name** | **Sens** | **Spec** | **AUC** |
| --- | --- | --- | --- | --- | --- | --- | --- | --- | --- | --- | --- |
| 1 | Zhao Y, et al (91). | 20-Dec-19 | PubMed, Cochrane, CNKI and Wanfang library | 404 | 301 | 4 | Moderate to high | MALAT1 | 0.78 | 0.78 | 0.84 |
| 2 | Zhang WT, et al (92). | 23-Oct-20 | PubMed, EMBASE, WOS, Cochrane, Google Scholar, Wanfang and CNKI | 500 | 292 | 6 | Moderate to high | let-7 family | 0.95 | 0.86 | 0.96 |
| 3 | Xie S, et al (3). | 25-Sep-15 | PubMed, EMBASE and WOS | 723 | 560 | 8 | high | miRNAs | 0.88 | 0.84 | 0.93 |
| 4 | Wang Y, et al (93). | 10-Apr-17 | VIP, WanFang, China National Knowledge Infrastructure (CNKI), Cochrane Library, PubMed, Web of Science, Embase | 813 | 478 | 9 | high | miR-155 | 0.89 | 0.82 | 0.92 |
| 5 | Tang S, et al (76). | Sep, 2016 | Medline and Web of Science | 2287 | 1644 | 27 | high | 1 lncRNA and 47 miRNAs | 0.83 | 0.8 | 0.90 |
| 6 | Liu Y, et al (10). | 24-May-19 | PubMed, Scopus, Embase, the Cochrane Library, BioMed Central, ISI Web of Knowledge, China National Knowledge Infrastructure, Wan Fang Data and Technology of Chongqing | 464 | 287 | 6 | high | miR-195 | 0.79 | 0.86 | 0.92 |
| 7 | Liu X, et al (94). | 1-May-20 | PubMed, EMBASE and Web of Science | 1,316 | 666 | 18 | Moderate to high | miR-155 | 0.87 | 0.82 | 0.91 |
| 8 | Liu F, et al (71). | 1-Aug-20 | Web of Science, EMBASE, PubMed, Cochrane Library and CNKI | 795 | 488 | 8 | Moderate to high | circRNAs | 0.75 | 0.76 | 0.82 |
| 9 | Gao Y, et al (95). | 3 March, 2016 | PubMed, Embase and Cochrane Library | 408 | 234 | 7 |  | miR-21 | 0.85 | 0.85 | 0.89 |
| 10 | Yu G, et al (96). | 1-Aug-17 | PubMed, EMBASE, Wed of Science, China National Knowledge Infrastructure, and Wanfang | 835 | 725 | 10 | High | lncRNAs | 0.79 | 0.8 | 0.86 |
|  |  |  |  | 320 | 207 |  |  | MALAT1 | 0.83 | 0.63 | 0.84 |
|  |  |  |  | 315 | 284 |  |  | H19 | 0.67 | 0.79 | 0.8 |
|  |  |  |  | 244 | 246 |  |  | HOTAIR | 0.74 | 0.89 | 0.86 |
| 11 | Jiang X, et al (12). | 1-Jun-19 | PubMed, EMBASE, Cochrane Library, Web of Science, Chinese National Knowledge Infrastructure Database (CNKI), VIP, and wanfang | 2425 | 2075 | 33 | High | lncRNAs | 0.74 | 0.78 | 0.82 |
|  |  |  |  | 744 | 526 |  |  | MALAT1 | 0.81 | 0.81 | 0.88 |
|  |  |  |  | 246 | 182 |  |  | H19 | 0.69 | 0.75 | 0.73 |
|  |  |  |  | 344 | 350 |  |  | HOTAIR | 0.78 | 0.85 | 0.82 |
| 12 | Xin H, et al (97). | 1-Apr-14 | PubMed, Embase, Sinomed, and Chinese National Knowledge Infrastructure (CNKI) | 1428 | 952 | 15 | Moderate to high | miRNAs | 0.76 | 0.87 | 0.88 |
| 13 | Liu L, et al (98). | 25-Apr-14 | PubMed, Medline, Embase, Chines e National Knowledge Infrastructure (CNKI), and Chinese Biology Medicine (CBM) | 1,668 | 1,111 | 16 | Moderate to high | miRNAs | 0.77 | 0.88 | 0.89 |
| 14 | Xin ZC, et al (99). | 2017 | PubMed, Embase, EBSCO and Cochrane | 2351 | 2230 | 14 | Moderate to high | miRNAs | 0.85 | 0.77 | 0.88 |
| 15 | Imani S, et al (100). | 12-Jul-16 | PubMed, ISI Web of Science, Google Scholar, vendor information pages database, and Embase | 1858 | 494 | 9 | Moderate to high | miR-34a | 85.5 | 70 | 0.8 |
| 16 | Li S, et al (101). | 10-Jun-14 | PubMed, Scopus, Embase, the Cochrane Library, BioMed Central, ISI Web of Knowledge, China National Knowledge Infrastructure, Wan Fang Data and Technology of Chongqing | 438 | 228 | 6 | High | miR-21 | 0.79 | 0.85 | 0.89 |
| 17 | Shen L, et al (102). | 1-Jul-14 | Medline, Embase, PubMed, CNKI, and Web of Science | 632 | 249 | 7 | High | miR-21 | 0.86 | 0.84 | 0.92 |
| 18 | Cai KT, et al (103). | 2018 |  | 1,077 | 104 | TCGA data |  | Precursor miR‑203a | 59.24% | 89.42% | 0.77 |
|  |  |  |  | 756 | 76 | UCSC Xena data |  | miR‑203a‑3p | 61.51% | 88.16% | 0.75 |
|  |  |  |  | 2,444 | 559 | TCGA, UCSC Xena, and GEO |  | miR‑203a‑3p | 0.7 | 0.81 | 0.82 |
| 19 | Cui Z, et al (104). | 31-Mar-14 | PubMed and EMBASE | 1368 | 849 | 15 | High | miRNAs | 0.82 | 0.82 | 0.92 |
| 20 | Hou Y, et al (105). | 5-Jul-15 | PubMed, Embase, Cochrane, Sinomed and Wanfang | 460 | 169 | 6 | High | miR-155 | 0.838 | 0.875 | 0.92 |
| 21 | Gao Y, et al (106). | 9-Nov-15 | PubMed, Embase, Chinese National Knowledge Infrastructure (CNKI), Wan Fang Data, and VIP database | 918 | 613 | 10 | High | miR-21 | 0.72 | 0.8 | 0.85 |
| 22 | Wang F, et al (107). | 30-Jan-14 | PubMed, Embase, EBSCO (ASP/BSP), Cochrane Library and China National Knowledge Infrastructure (CNKI) | 184 | 75 | 3 | High | miR-155 | 0.79 | 0.85 | 0.92 |
