# Supplementary material for: Prognostic and diagnostic values of non-coding RNAs as biomarkers for breast cancer: An umbrella review and pan-cancer analysis
Source: Front Mol Biosci. 2023 Jan 16;10:1096524. doi: 10.3389/fmolb.2023.1096524 (PMC9885171; doi:10.3389/fmolb.2023.1096524)
Supplement: Supplementary file 2 [file DataSheet2.ZIP › Supplementary Material, Table 12.docx]

| **Parameter** | **Sensitivity**  **(95% CI)** | ***P*-value** | **Specificity**  **(95% CI)** | ***P*-value** | **Category** | **LRTChi^2^** | ***P*-value** | **I^2^** | **I^2^ lo** | **I^2^ hi** |
| --- | --- | --- | --- | --- | --- | --- | --- | --- | --- | --- |
| Sample Size | 0.80 [0.77 - 0.83] | 1.00 | 0.82 [0.80 - 0.84] | 1.00 |  | 1.28 | 0.53 | 0 | 0 | 100 |
| AMSTAR Score | 0.81 [0.77 - 0.84]  0.78 [0.74 - 0.83] | 0.00  . | 0.82 [0.80 - 0.85]  0.82 [0.79 - 0.85] | 0.00  . | Yes  No | 0.83  . | 0.66  . | 0  . | 0  . | 100  . |
| miRNAs vs. LncRNAs | 0.81 [0.78 - 0.85]  0.76 [0.71 - 0.82] | 0.00  . | 0.84 [0.81 - 0.86]  0.79 [0.76 - 0.83] | 0.00  . | Yes  No | 28.53  . | 0.00  . | 93  . | 87  . | 99  . |
| Single miRNA vs. combined miRNAs  Single LncRNA vs. combined LncRNAs | 0.79 [0.75 - 0.84]  0.85 [0.80 - 0.90]  0.76 [0.73 - 0.80]  0.77 [0.70 - 0.83] | 0.00  .  0.00  . | 0.83 [0.81 - 0.86]  0.84 [0.81 - 0.86]  0.79 [0.75 - 0.84]  0.79 [0.71 - 0.87] | 0.00  .  0.00  . | Yes  No  Yes  No | 203.52  .  434.67  . | 0.00  .  0.00  . | 99  .  100  . | 99  .  99  . | 100  .  100  . |
|  |  |  |  |  |  |  |  |  |  |  |

**Supplementary Material, Table 12.** The results of meta-regression analysis
