# Supplementary material for: Prognostic and diagnostic values of non-coding RNAs as biomarkers for breast cancer: An umbrella review and pan-cancer analysis
Source: Front Mol Biosci. 2023 Jan 16;10:1096524. doi: 10.3389/fmolb.2023.1096524 (PMC9885171; doi:10.3389/fmolb.2023.1096524)
Supplement: Supplementary file 2 [file DataSheet2.ZIP › Supplementary Material, Table 15.docx]

**Supplementary Material, Table 15.** The expression level of ncRNAs (miRNAs and LncRNAs) and mRNAs that showed a significant difference between normal individuals and cancer patients in Pan-Cancer data.

| miR/Lnc/Gene | Cancer | CancerFullName | CancerNum | NormalNum | CancerExp | NormalExp | FoldChange | pValue | FDR |
| --- | --- | --- | --- | --- | --- | --- | --- | --- | --- |
| has-miR-21-5p | BRCA | Breast Invasive Carcinoma | 1085 | 104 | 2657.21 | 618.57 | 4.3 | 7.20E-96 | 2.30E-93 |
| hsa-miR-19a-3p | BRCA | Breast Invasive Carcinoma | 1085 | 104 | 35.64 | 14.06 | 2.53 | 1.30E-10 | 1.10E-09 |
| hss-miR-92a-3p | BRCA | Breast Invasive Carcinoma | 1085 | 104 | 9279.36 | 10326.74 | 0.9 | 0.00014 | 0.00064 |
| hsa-miR-19b-1-5p | BRCA | Breast Invasive Carcinoma | 1085 | 104 | 7.71 | 4.77 | 1.62 | 0.0014 | 0.0051 |
| hsa-miR-200c-3p | BRCA | Breast Invasive Carcinoma | 1085 | 104 | 12410.76 | 4304.51 | 2.88 | 2.60E-65 | 2.80E-63 |
| hsa-miR-203 | BRCA | Breast Invasive Carcinoma | 1085 | 104 | 7872.52 | 1727.97 | 4.56 | 4.40E-18 | 6.90E-17 |
| hsa-miR-210 | BRCA | Breast Invasive Carcinoma | 1085 | 104 | 818.74 | 87.82 | 9.32 | 9.00E-32 | 3.10E-30 |
| hsa-miR-22 | BRCA | Breast Invasive Carcinoma | 1085 | 104 | 69171.9 | 103722.25 | 0.67 | 1.10E-15 | 1.40E-14 |
| hsa-miR-221 | BRCA | Breast Invasive Carcinoma | 1085 | 104 | 125.27 | 126.09 | 0.99 | 0.00049 | 2.00E-03 |
| hsa-miR-375 | BRCA | Breast Invasive Carcinoma | 1085 | 104 | 24627.49 | 3895.65 | 6.32 | 3.90E-23 | 8.30E-22 |
| hsa-miR-93-5p | BRCA | Breast Invasive Carcinoma | 1085 | 104 | 5733.21 | 2498.57 | 2.29 | 4.80E-26 | 1.20E-24 |
| hsa-miR-125b-5p | BRCA | Breast Invasive Carcinoma | 1085 | 104 | 723.36 | 2660.52 | 0.27 | 1.50E-70 | 2.10E-68 |
| hsa-miR-484 | BRCA | Breast Invasive Carcinoma | 1085 | 104 | 98.73 | 70.55 | 1.4 | 3.10E-06 | 1.70E-05 |
| hsa-miR-520h | BRCA | Breast Invasive Carcinoma | 1085 | 104 | 0.23 | 0.27 | 0.86 | 5.00E-10 | 4.30E-09 |
| hsa-miR-4443 | BRCA | Breast Invasive Carcinoma | 1085 | 104 | 0.38 | 0.16 | 2.43 | 6.50E-05 | 0.00032 |
| hsa-miR-489 | BRCA | Breast Invasive Carcinoma | 1085 | 104 | 0.17 | 0.24 | 0.7 | 0.00028 | 0.0012 |
| HOTAIR | BRCA | Breast Invasive Carcinoma | 1104 | 113 | 3.11 | 0.51 | 6.11 | 5.40E-14 | 3.30E-13 |
| LINC00511 | BRCA | Breast Invasive Carcinoma | 1104 | 113 | 0.79 | 0.12 | 6.49 | 2.40E-29 | 3.20E-28 |
| NEAT1 | BRCA | Breast Invasive Carcinoma | 1104 | 113 | 28.82 | 22.63 | 1.27 | 0.0065 | 0.015 |
| DANCR | BRCA | Breast Invasive Carcinoma | 1104 | 113 | 17.87 | 12.8 | 1.4 | 5.30E-06 | 1.80E-05 |
| PVT1 | BRCA | Breast Invasive Carcinoma | 1104 | 113 | 3.91 | 1.52 | 2.57 | 2.90E-37 | 5.30E-36 |
| E2F3 | BRCA | Breast Invasive Carcinoma | 1104 | 113 | 6.46 | 3.35 | 1.93 | 2.40E-20 | 2.00E-19 |
| SMAD4 | BRCA | Breast Invasive Carcinoma | 1104 | 113 | 5.44 | 7.63 | 0.71 | 3.10E-23 | 3.10E-22 |
| BTG2 | BRCA | Breast Invasive Carcinoma | 1104 | 113 | 103.75 | 147.52 | 0.7 | 2.90E-11 | 1.40E-10 |
| IRAK1 | BRCA | Breast Invasive Carcinoma | 1104 | 113 | 36.45 | 20.44 | 1.78 | 2.00E-19 | 1.60E-18 |
| VEGFA | BRCA | Breast Invasive Carcinoma | 1104 | 113 | 9.18 | 5.33 | 1.72 | 7.30E-09 | 3.10E-08 |
