# Supplementary material for: Prognostic and diagnostic values of non-coding RNAs as biomarkers for breast cancer: An umbrella review and pan-cancer analysis
Source: Front Mol Biosci. 2023 Jan 16;10:1096524. doi: 10.3389/fmolb.2023.1096524 (PMC9885171; doi:10.3389/fmolb.2023.1096524)
Supplement: Supplementary file 2 [file DataSheet2.ZIP › Supplementary Material, Table 16.docx]

| Variables (mRNA) | Survival Outcomes | Cancer | HR (95%CI) | logRank P |
| --- | --- | --- | --- | --- |
| E2F3 | OS | BRCA | 1.41 (1.01 − 1.97) | 0.043 |
|  | RFS | BRCA | 1.82 (1.17 − 2.81) | 0.0066 |
| SMAD4 | OS | BRCA | 1.26 (0.91 − 1.75) | 0.16 |
|  | RFS | BRCA | 0.63 (0.41 − 0.98) | 0.039 |
| SMAD7 | OS | BRCA | 1.21 (0.88 − 1.67) | 0.23 |
|  | RFS | BRCA | 1.54 (0.99 − 2.4) | 0.052 |
| BTG2 | OS | BRCA | 0.53 (0.36 − 0.78) | 0.00086 |
|  | RFS | BRCA | 0.5 (0.32 − 0.78) | 0.0018 |
| IRAK1 | OS | BRCA | 1.58 (1.12 − 2.21) | 0.0077 |
|  | RFS | BRCA | 1.81 (1.16 − 2.81) | 0.0075 |
| VEGFA | OS | BRCA | 1.22 (0.86 − 1.75) | 0.26 |
|  | RFS | BRCA | 1.39 (0.88 − 2.21) | 0.16 |
| UBR5 | OS | BRCA | 1.72 (1.13 − 2.61) | 0.011 |
|  | RFS | BRCA | 1.43 (0.89 − 2.3) | 0.14 |
| Total | OS | BRCA | 0.75 (0.54 − 1.03) | 0.078 |
|  | RFS | BRCA | 1.31 (0.85 − 2.01) | 0.22 |

| Variables (LncRNA) | Survival Outcomes | Cancer | HR (95%CI) | logRank P |
| --- | --- | --- | --- | --- |
| DANCR | OS | BRCA | 1.41 (1.01 − 1.96) | 0.041 |
|  | RFS | BRCA | NS* | NS* |
| HOTAIR | OS | BRCA | 1.62 (1.15 − 2.29) | 0.0051 |
|  | RFS | BRCA | 1.62 (1.06 − 2.49) | 0.026 |
| NEAT1 | OS | BRCA | 0.65 (0.45 − 0.93) | 0.016 |
|  | RFS | BRCA | 0.64 (0.41 – 1) | 0.049 |
| LINC00511 | OS | BRCA | 1.51 (1.1 − 2.09) | 0.011 |
|  | RFS | BRCA | 1.6 (1.04 − 2.48) | 0.033 |

*NS = non-Significant

| Variables (miRNA) | Survival Outcomes | Cancer | HR (95%CI) | logRank P |
| --- | --- | --- | --- | --- |
| has-miR-19a | OS | BRCA | 1.54 (1.1 − 2.13) | 0.01 |
| has-miR-21 | OS | BRCA | 1.63 (1.16 − 2.27) | 0.004 |
| has-miR-22 | OS | BRCA | 1.95 (1.39 − 2.72) | 7.8e-05 |
| hsa-miR-146a | OS | BRCA | 0.71 (0.51 − 0.98) | 0.038 |
| has-miR-203a | OS | BRCA | 1.44 (1.04 − 1.99) | 0.027 |
| has-miR-484 | OS | BRCA | 1.68 (1.17 − 2.43) | 0.0047 |
| has-miR-489 | OS | BRCA | 1.81 (1.27 − 2.57) | 8e-04 |
| hsa-miR-520h | OS | BRCA | 2.1 (1.5 − 2.93) | 9.8e-06 |
| hsa-miR-4443 | OS | BRCA | 1.71 (1.13 − 2.59) | 0.01 |
| Total | OS | BRCA | 1.8 (1.3 − 2.5) | 0.00038 |
