# Supplementary material for: Prognostic and diagnostic values of non-coding RNAs as biomarkers for breast cancer: An umbrella review and pan-cancer analysis
Source: Front Mol Biosci. 2023 Jan 16;10:1096524. doi: 10.3389/fmolb.2023.1096524 (PMC9885171; doi:10.3389/fmolb.2023.1096524)
Supplement: Supplementary file 2 [file DataSheet2.ZIP › Supplementary Material, Table 3.docx]

**Scopus**

(TITLE-ABS-KEY ("breast carcinoma*") OR TITLE-ABS-KEY ("breast cancer*") OR TITLE-ABS-KEY("breast*") OR TITLE-ABS-KEY("breast tumor") OR TITLE-ABS-KEY("breast cancer ") OR TITLE-ABS-KEY("breast*" AND "cancer*") OR TITLE-ABS-KEY("breast*" AND " carcinoma*")) AND (TITLE-ABS-KEY (prognosis*) OR TITLE-ABS-KEY("prognosis") OR TITLE-ABS-KEY("prognostic*") OR TITLE-ABS-KEY("prognostic value *")) AND (TITLE-ABS-KEY("RNA, Untranslated") OR TITLE-ABS-KEY("RNA, Noncoding") OR TITLE-ABS-KEY("Non-coding RNA*") OR TITLE-ABS-KEY("RNA" AND "micro") OR TITLE-ABS-KEY("RNA" AND "untranslated") OR TITLE-ABS-KEY("RNA" AND "nontranslated") OR TITLE-ABS-KEY("non" AND "coding" AND "RNA") OR TITLE-ABS-KEY("noncoding RNA") OR TITLE-ABS-KEY(“ncRNA*") OR TITLE-ABS-KEY(RNA, Small Temporal) OR TITLE-ABS-KEY(stRNA*) OR TITLE-ABS-KEY(Temporal RNA, Small) OR TITLE-ABS-KEY(microarray analysis) OR TITLE-ABS-KEY("RNA, Small Untranslated") OR TITLE-ABS KEY("MicroRNAs*") OR TITLE-ABS-KEY(miRNA*) OR TITLE-ABS-KEY(miR) OR TITLE-ABS-KEY("Circulating MicroRNA*") OR TITLE-ABS-KEY("RNA, Long Noncoding") OR TITLE-ABS-KEY("long non coding RNA") OR TITLE-ABS-KEY("RNA" AND "long" AND "noncoding") OR TITLE-ABS-KEY("long non coding RNA") OR TITLE-ABS-KEY("RNA" AND "long" AND "non" AND "coding") OR TITLE-ABS-KEY("lncRNA*") OR TITLE-ABS-KEY(“long non-coding RNA”) OR TITLE-ABS-KEY(long noncoding RNAs) OR TITLE-ABS-KEY(LincRNA*) OR TITLE-ABS-KEY(“LINC RNA”))

**WOS**

(TS=("breast carcinoma") OR TS=("breast cancer") OR TS=(“breast”) OR TS=("breast tumor") OR TS=("breast" AND "carcinoma") OR TS=("breast" AND "cancer")) AND (TS=(prognosis) OR TS=(prognostic) OR TS=(prognostic value)) AND (TS=("RNA, Untranslated") OR TS=("RNA, Noncoding") OR TS=("Non-coding RNA*") OR TS=("RNA" AND "micro") OR TS=("RNA" AND "untranslated") OR TS=("RNA" AND "nontranslated") OR TS=("non" AND "coding" AND "RNA") OR TS=("noncoding RNA") OR TS=(“ncRNA*") OR TS=("RNA, Small Untranslated") OR TS=("MicroRNAs*") OR TS=(miRNA*) OR TS=(miR) OR TS=("Circulating MicroRNA*") OR TS=("RNA, Long Noncoding") OR TS=("long non coding RNA") OR TS=("RNA" AND "long" AND "noncoding") OR TS=("long non coding RNA") OR TS=("RNA" AND "long" AND "non" AND "coding") OR TS=("lncRNA*") OR TS=(“long non-coding RNA”) OR TS=(long noncoding RNAs) OR TS=(LincRNA*) OR TS=(“LINC RNA”))

(ALL=("breast carcinoma") OR ALL=("breast cancer") OR ALL=(breast) OR ALL=("breast tumor") OR ALL=("breast" AND "carcinoma") OR ALL=("breast" AND "cancer")) AND (ALL=(prognosis) OR ALL=(prognostic) OR ALL=(prognostic value)) AND (ALL=("RNA, Untranslated") OR ALL=("RNA, Noncoding") OR ALL=("Non-coding RNA*") OR ALL=("RNA" AND "micro") OR ALL=("RNA" AND "untranslated") OR ALL=("RNA" AND "nontranslated") OR ALL=("non" AND "coding" AND "RNA") OR ALL=("noncoding RNA") OR ALL=(“ncRNA*") OR ALL=("RNA, Small Untranslated") OR ALL=("MicroRNAs*") OR ALL=(miRNA*) OR ALL=(miR) OR ALL=("RNA, Long Noncoding") OR ALL=("long non coding RNA") OR ALL=("RNA" AND "long" AND "noncoding") OR ALL=("long non coding RNA") OR ALL=("RNA" AND "long" AND "non" AND "coding") OR ALL=("lncRNA*") OR ALL=(“long non-coding RNA”) OR ALL=(long noncoding RNAs) OR ALL=(LincRNA*) OR ALL=(“LINC RNA”))
