# Supplementary material for: Prognostic and diagnostic values of non-coding RNAs as biomarkers for breast cancer: An umbrella review and pan-cancer analysis
Source: Front Mol Biosci. 2023 Jan 16;10:1096524. doi: 10.3389/fmolb.2023.1096524 (PMC9885171; doi:10.3389/fmolb.2023.1096524)
Supplement: Supplementary file 2 [file DataSheet2.ZIP › Supplementary Material, Table 5.docx]

**Supplementary Material, Table 5.** Characteristics of included systematic review and meta-analysis studies.

| **N** | **References** | **Year** | **No. of Cases** | **Biomarkers name** | **No.of Study Estimates** | **Association Between Biomarker and Breast Cancer** | **Effect**  **Metrics** | **Summary relative**  **risk estimate (95%CI)** |
| --- | --- | --- | --- | --- | --- | --- | --- | --- |
| 1 | Wang X-F, et al. (33) | 2020 | 207 | LINC00511 | 3 | OS | HR | 2.57 (1.55-4.27) |
| 2 | Qattan A, et al. (34) | 2021 | 2502 | miR-93 | 4 | OS | HR | 1.47 (1.24-1.73) |
|  |  |  | 2502 | miR-210 |  | OS | HR | 1.6 (1.35-1.89) |
|  |  |  | 2502 | miR-19a |  | OS | HR | 1.45 (1.12-187) |
|  |  |  | 2502 | miR-19b |  | OS | HR | 1.31 (1.11-1.55) |
|  |  |  | 2502 | miR-25 |  | OS | HR | 1.2 (0.85-1.69) |
|  |  |  | 2502 | miR-22 |  | OS | HR | 0.96 (0.55-1.67) |
|  |  |  | 1347 | miR-199a-3 |  | OS | HR | 1.33 (0.36-4.89) |
| 3 | Wang Y, et al. (35) | 2016 | 252 | MALAT-1 | 3 | RFS | HR | 1.97 (1.25-3.09) |
| 4 | Wang F, et al. (36) | 2019 | 468 | miRNA-200c | 4 | OS | HR | 2.69 (1.49-4.85) |
|  |  |  | 631 | miRNA-200c | 5 | PFS/DFS | HR | 1.66 (1.03-2.67) |
| 5 | Zou B, et al. (37) | 2019 | 168 | PVT1 | 2 | OS | HR | 1.96 (1.21-3.18) |
| 6 | Liao M, et al. (38) | 2017 | 1767 | LINC00341 | 9 |  | HR | 3.04 (2.23-4.16) |
| 7 | Liu F, et al. (39) | 2017 | 450 | miR-17-92 family | 3 | DFS | HR | 1.93 (0.31-11.87) |
| 8 | Zhou X, et al. (40) | 2014 | 804 | miR-21 | 6 | OS | HR | 2.55 (1.04-6.29) |
|  |  |  | 666 | miR-21 | 4 | DFS | HR | 1.1 (0.82-1.49) |
| 9 | Tuluhong D, et al. (41) | 2020 | 2192 | lncRNAs | 21 | OS | HR | 2.38 (2.03-2.78) |
|  |  |  | 2192 | lncRNAs |  | DFS | HR | 2.19 (1.51-3.16) |
|  |  |  | 2192 | lncRNAs |  | RFS | HR | 3.19 (0.81-12.53) |
| 10 | Tang W, et al. (42) | 2019 |  | miR-375 |  | OS | HR | 1.29 (1.04-1.6) |
|  |  |  |  | miR-375 |  | OS | HR | 1.23 (0.89-1.7) |
| 11 | He X, et al. (43) | 2021 |  | MALAT1 | 6 | RFS | HR | 1.51 (0.79-2.29) |
|  |  |  |  | MALAT1 |  | OS | HR | 1.09 (0.63-1.72) |
| 12 | Liu K, et al. (44) | 2019 | 470 | miR-221 | 2 | OS | HR | 2.02 (0.22-18.81) |
| 13 | Xiang Q, et al. (45) | 2019 | 2282 | miR-22 | 5 | OS | HR | 0.81 (0.54-1.22) |
|  |  |  | 2282 | miR-22 |  | PRS/RFS/DFS | HR | 0.86 (0.3-2.47) |
| 14 | Li Y, et al. (46) | 2021 | 263 | AWPPH | 2 | OS | HR | 2.01 (1-3) |
| 15 | Lee JS, et al. (47) | 2017 | 335 | miR-200 | 2 | PFS | HR | 2.87 (1.43-5.73) |
| 16 | Huang B, et al. (48) | 2020 | 187 | CCAT2 | 2 | OS | HR | (1.36-2.6) |
| 17 | Escala-Garcia M, et al. (49) | 2019 | 96661 | rs67918676 long intergenic non-coding RNA gene, LOC105375207 | 12 | OS | HR | 1.27 (1.16-1.39) |
| 18 | Li MW, et al. (50) | 2019 | 1127 | miR-146a-5p | 4 | OS | HR | 0.774 (0.406-1.474) |
| 19 | Wang W, et al. (51) | 2014 | 334 | microRNA-21 | 3 | OS | HR | 4.08 (1.23-13.48) |
| 20 | Binabaj MM, et al. (52) | 2020 | 334 | miR-21 |  | OS | HR | 2.2 (1.78-2.73) |
| 21 | Wang Z, et al. (53) | 2018 | 2958 | MALAT | 10 | RFS | HR | 1.19 (0.92-1.55) |
|  |  |  | 2225 | MALAT | 7 | OS | HR | 1.16 (0.92-1.45) |
| 22 | Mongre RK, et al. (54) | 2019 | 626 | SERTAD1 |  | OS | HR | 0.78 |
|  |  |  | 626 | SERTAD1 |  | RFS | HR | 0.72 |
|  |  |  | 26 | SERTAD1 |  |  | HR | 0.56 |
| 23 | Wang Y, et al. (55) | 2020 | 1072 | MALAT-1 | 6 | OS | HR | 2.06 (1.66-2.56) |
|  |  |  | 3487 | MALAT-1 | 6 | DFS/RFS/MFS/DSS | HR | 0.0093 (0.0013-0.0637) |
|  |  |  | 3041 | MALAT-1 | 5 | DFS/RFS/DSS | HR | 1.91 (1.53-2.39) |
| 24 | Wang Y, et al. (56) | 2017 | 252 | AFAP1-AS1 | 3 | RFS | HR | 2.9 (1.69-4.98) |
| 25 | Tian T, et al. (57) | 2018 | 2428 | CCAT2 | 32 | OS | HR | 1.29 (1.03-1.63) |
|  |  |  | 386 | MALAT1 |  | OS | HR | 2.78 (1.95-3.97) |
|  |  |  | 228 | NEAT1 |  | OS | HR | 1.65 (1.08-2.54) |
|  |  |  | 554 | MEG3 |  | OS | HR | 0.47 (0.37-0.71) |
|  |  |  | 498 | HOTAIR |  | OS | HR | 1.29 (1.03-1.63) |
|  |  |  | 2370 | CCAT2 |  | MFS | HR | 1.18 (1.02-1.36) |
|  |  |  | 1108 | HOTAIR |  | MFS | HR | 1.9 (1.41-2.55) |
| 26 | Shen Y, et al. (58) | 2016 |  | LINC00472 | 9 | DFS | HR | 0.49 (0.38-0.63) |
| 27 | Zhang S, et al. (59) | 2020 | 3146 | lncRNAs | 31 | OS | HR | 1.86 (1.45-2.27) |
|  |  |  | 3146 | lncRNAs |  | OS | HR | 1.41 (1.06-1.75) |
|  |  |  | 3146 | GAS5, NEF and MIR503HG |  | OS | HR | 0.6 (0.43-0.77) |
|  |  |  | 3146 | AFAP1-AS1, LINC00511, HOTAIR, linc-ZNF469–3 |  | DFS | HR | 1.85 (1.37-2.33) |
| 28 | Binabaj MM, et al. (60) | 2018 | 464 | MEG3 | 2 | OS | HR | 0.85 (0.12-5.88) |
| 29 | Shen Y, et al. (61) | 2015 | 4628 | LINC00472 | 13 | OS | HR | 0.91 (0.57-1.45) |
|  |  |  | 4628 | LINC00472 |  | OS | HR | 0.41 (0.22-0.78) |
|  |  |  | 4628 | LINC00472 |  | DFS | HR | 0.69 (0.55-0.88) |
|  |  |  | 4628 | LINC00472 |  |  | HR | 0.43 (0.31-0.59) |
| 30 | Zhou L, et al. (62) | 2019 | 730 | FOXD2-AS1 | 5 | OS | HR | 1.55 (1.14-2.11) |
| 31 | Li J, et al. (63) | 2015 | 498 | HOTAIR | 3 | OS/DFS/RFS/MFS | HR | 1.38 (0.36-5.27) |
| 32 | Tu C, et al. (64) | 2019 | 240 | BCAR4 | 2 | OS | HR | 2.44 (1.22-4.85) |
| 33 | Li S, et al. (65) | 2019 |  | TINCR | 2 | OS | HR | 1.582 (1.126-2.223) |
| 34 | Zhang T, et al. (66) | 2018 | 276 | TUBA4B | 3 | OS | HR | 1.52 (1.1-2.12) |
|  |  |  | 252 | TUBA4B |  | RFS | HR | 1.49 (1.14-1.94) |
|  |  |  | 397 | TUBA4B |  | DFS | HR | 1.23 (1.01-1.49) |
| 35 | Wang J, et al. (67) | 2014 | 707 | miR-210 | 7 | OS | HR | 1.63 (0.47-5.67) |
|  |  |  | 478 | miR-210 |  | DFS | HR | 2.03 (0.9-4.57) |
| 36 | Shao Y, et al. (68) | 2017 | 413 | miR-203 | 2 | OS | HR | 2.26 (1.47-3.48) |
|  |  |  | 1027 | miR-203 |  | OS | HR | 1.25 (0.87-1.81) |
| 37 | Li M, et al. (69) | 2014 | 268 | miR-210 | 6 | OS | HR | 3.29 (1.65-6.58) |
|  |  |  | 542 | miR-210 |  | DFS/RFS | HR | 3.36 (2.3-4.93) |
|  |  |  | 624 | miR-210 |  | MFS/DRFS | HR | 2.85 (1.76-4.62) |
| 38 | Jayaraj R, et al. (70) | 2019 | 852 | miRNAs | 6 | OS | HR | 0.748 (0.508-1.1) |
| 39 | Liu F, et al. (71) | 2021 | 1690 | circRNAs | 26 | OS/DFS | HR | 0.32 (0.23-0.44) |
|  |  |  | 1920 | circRNAs |  | OS/DFS | HR | 2.43 (2.2-2.92) |
| 40 | Ma JY, et al. (72) | 2021 | 1104 | FOXD2.AS1 |  |  | HR | 1.496 (1.013-2.209) |
|  |  |  |  | A1BG.AS1 |  |  | HR | 0.635 (0.39-1.031) |
|  |  |  |  | C9orf163 |  |  | HR | 2.726 (1.392-5.336) |
|  |  |  |  | GSN.AS1 |  |  | HR | 2.104 (1.302-3.403) |
|  |  |  |  | LINC00893 |  |  | HR | 0.25 (0.097-0.641) |
| 41 | Jishnu PV, et al. (73) | 2019 | 750 | miRNAs | 4 | OS | HR | 2.12 (1.47-3.04) |
|  |  |  | 410 | miRNAs |  | OS | HR | 3.48 (1.96-6.19) |
| 42 | Huang Q, et al. (74) | 2017 | 2757 | miR-10b | 3 | OS | HR | 1.853 (1.521-2.258) |
|  |  |  | 292 | miR-10b |  | RFS | HR | 2.692 (0.877-8.265) |
|  |  |  | 704 | miR-10b |  | DFS | HR | 1.309 (0.699-2.453) |
| 43 | Huang GL, et al. (75) | 2019 | 731 | miR-200 family | 3 | OS/PFS | HR | 2.122 (1.533-2.936) |
|  |  |  | 731 | miR-141 |  | OS/PFS | HR | 2.005 (0.987-4.074) |
|  |  |  | 569 | miR-200a |  | OS/PFS | HR | 3.126 (2.313-4.224) |
|  |  |  | 569 | miR-200b |  | OS/PFS | HR | 2.462 (1.488-4.073) |
|  |  |  | 626 | miR-200c |  | OS/PFS | HR | 1.684 (1.248-2.273) |
|  |  |  | 569 | miR-429 |  | OS/PFS | HR | 1.971 (0.656-5.917) |
| 44 | Tang S, et al. (76) | 2017 | 4472 | ncRNAs | 51 | OS/PFS | HR | 0.33 (0.23-0.47) |
|  |  |  | 6854 | ncRNAs |  | OS/PFS | HR | 2.63 (2.27-3.05) |
|  |  |  | 2605 | ncRNAs |  | PFS/RFS | HR | 0.68 (0.53-0.87) |
|  |  |  | 7447 | ncRNAs |  | PFS/RFS | HR | 2.7 (1.91-3.81) |
|  |  |  | 57 | ncRNAs |  |  | HR | 0.4 (0.17-0.94) |
|  |  |  | 442 | ncRNAs |  |  | HR | 2.09 (1.41-3.11) |
|  |  |  |  | MALAT1 |  | RFS | HR | 2.36 (1.55-3.6) |
|  |  |  |  | miR-21 |  | OS | HR | 1.97 (1.55-2.44) |
|  |  |  |  | miR-200a |  | OS | HR | 3.24 (1.3-8.07) |
|  |  |  |  | miR-200b |  | OS | HR | 2.08 (0.54-8.01) |
|  |  |  |  | miR-200c |  | OS | HR | 3.41 (1.91-6.09) |
|  |  |  |  | miR-22 |  | OS | HR | 0.88 (0.36-2.19) |
|  |  |  |  | miR-124 |  | OS | HR | 0.71 (0.55-0.92) |
|  |  |  |  | miR-210 |  | OS | HR | 0.71 (0.55-0.92) |
|  |  |  |  | HOTAIR |  | OS | HR | 1.21 (0.16-8.93) |
| 45 | Wang D, et al. (77) | 2020 | 1107 | LINC0164 | 1 | OS | HR | 1.452 (1.034-2.039) |
| 46 | Zhang JY, et al. (78) | 2014 | 772 | miRNA-205 | 4 | OS | HR | 0.78 (0.67-0.91) |
|  |  |  | 191 | miRNA-205 |  | DFS/RFS | HR | 0.74 (0.32-1.67) |
| 47 | Liu Y, et al. (79) | 2017 | 672 | miR-210 | 6 | OS | HR | 2.67 (1.24-5.76) |
| 48 | Huang M, et al. (80) | 2020 | 127 | miR-153 | 2 |  | HR | 4.01 (1.46-11.04) |
| 49 | Pan F, et al. (81) | 2014 | 951 | MiR-21 | 6 | OS | HR | 2.11 (1.09-4.08) |
|  |  |  | 813 | MiR-21 | 4 | DFS | HR | 1.6 (1.3-1.96) |
| 50 | Lü L, et al. (82) | 2017 | 2510 | miRNAs | 19 |  | HR |  |
|  |  |  | 822 | miR-155 |  | OS | HR | 0.67 (0.58-0.79) |
|  |  |  | 364 | miR-155 |  | OS | HR | 0.58 (0.34-0.99) |
|  |  |  | 276 | miR-21 |  | OS | HR | 2.5 (1.56-4.01) |
|  |  |  | 276 | miR-21 |  | DFS | HR | 1.99 (0.71-5.6) |
|  |  |  | 920 | miR-27a/b |  | OS | HR | 1.25 (0.98-1.61) |
|  |  |  | 765 | miR-27a/b |  | OS | HR | 2.38 (1.32-4.29) |
|  |  |  | 589 | miR-374a/b |  | DFS | HR | 0.77 (0.65-0.9) |
|  |  |  | 107 | miR-210 |  | OS | HR | 2.41 (1.15-5.08) |
|  |  |  | 208 | miR-454 |  | OS | HR | 6.74 (2.72-16.73) |
|  |  |  | 208 | miR-454 |  | DFS | HR | 3.72 (1.94-7.12) |
| 51 | Jinling W, et al. (83) | 2016 | 1629 | miR-21 | 7 | OS | HR | 1.51 (1.15-1.98) |
| 52 | Wu Z, et al. (84) | 2019 | 6979 | miR-205 | 6 | OS | HR | 0.84 (0.72-0.98) |
|  |  |  | 200 | miR-205 |  | PFS/DFS/DMFS/DSS | HR | 0.71 (0.48-1.07) |
| 53 | Wang N, et al. (85) | 2016 | 770 | miR-10b | 7 | DFS | RR | 1.53 (1.06-2.21) |
|  |  |  | 770 | miR-10b |  | OS | RR | 1.05 (0.67-1.64) |
| 54 | Liu Y, et al. (86) | 2017 | 1473 | miRNAs | 14 | OS | HR | 1.78 (0.97-3.25) |
| 55 | Tang Y, et al. (87) | 2015 | 5507 | miRNAs | 41 | OS | HR | 1.07 (1.02-1.12) |
|  |  |  | 1488 | miR-21 | 11 | OS | HR | 1.46 (1.25-1.7) |
|  |  |  | 1488 | miR-21 | 6 | DFS/RFS | HR | 1.49 (1.17-1.9) |
|  |  |  | 744 | miR-210 | 5 | OS/DFS/RFS | HR | 2.94 (2.08-4.18) |
| 56 | Li Y, et al. (88) | 2013 | 822 | miR-210 | 5 | DFS/RFS | HR | 3.47 (2.63-4.6) |
|  |  |  | 822 | miR-210 | 4 | OS | HR | 3.94 (1.9-8.15) |
|  |  |  | 822 | miR-210 | 2 | MFS | HR | 2.7 (1.46-5) |
| 57 | Hong, L., et al. (89) | 2012 | 511 | miR-210 | 4 | Survival outcome with a follow up time of more than 10 years | HR | 3.39 (2.04-5.63) |
| 58 | Wang Y, et al. (90) | 2015 | 1439 | miR-21 | 9 | OS | HR | 2.57 (1.37-4.81) |
|  |  |  | 1439 | miR-21 | 7 | DFS/RFS | HR | 1.45 (1.16-1.82) |
