# Supplementary material for: Prognostic and diagnostic values of non-coding RNAs as biomarkers for breast cancer: An umbrella review and pan-cancer analysis
Source: Front Mol Biosci. 2023 Jan 16;10:1096524. doi: 10.3389/fmolb.2023.1096524 (PMC9885171; doi:10.3389/fmolb.2023.1096524)
Supplement: Supplementary file 2 [file DataSheet2.ZIP › Supplementary Material, Table 7.docx]

**Supplementary Material, Table 7.** The result of meta-regression of OS.

| Variables | Coefficient | Std. err. | z | P>\|z\| | [95% conf. interval] |
| --- | --- | --- | --- | --- | --- |
| One variable at a time |  |  |  |  |  |
| AMSTAR  Low quality  Moderate quality  cons | -.0766533  -.0422941  .4519266 | .1964162  .1391099  .1049629 | -0.39  -0.30  4.31 | 0.696  0.761  0.000 | -.4616221 .3083154  -.3149444 .2303562  .2462031 .6576501 |
| Single/Combined lncRNAs  _cons | .0078172  .3426563 | .2600196  .2345412 | 0.03  1.46 | 0.976  0.144 | -.5018118 .5174462  -.117036 .8023486 |
| Single/Combined miRNAs  _cons | .0081707  .4872698 | .2284775  .2142112 | 0.04  2.27 | 0.971  0.023 | -.4396369 .4559783  .0674236 .907116 |
| lncRNAs/miRNAs  cons | .142821  .3487671 | .1260107  .1032138 | 1.13  3.38 | 0.257  0.001 | -.1041554 .3897975  .1464718 .5510625 |
| Grouped variables |  |  |  |  |  |
| No of cases  AMSTAR  Low quality  Moderate quality  lncRNAs/miRNAs  cons | -.0001707  -.0376956  -.1160716  .1408061  .6742136 | .0000422  .1899411  .1336672  .1374988  .1651243 | -4.04  -0.20  -0.87  1.02  4.08 | 0.000  0.843  0.385  0.306  0.000 | -.0002535 -.0000879  -.4099732 .3345821  -.3780544 .1459112  -.1286865 .4102987  .3505759 .9978513 |
| No of cases  AMSTAR  Low quality  Moderate quality  Single/Combined lncRNAs  cons | -.000215  .264606  -.0082816  -.1576289  .702946 | .0001104  .3943648  .353971  .4070785  .6730028 | -1.95  0.67  -0.02  -0.39  1.04 | 0.051  0.502  0.981  0.699  0.296 | -.0004313 1.35e-06  -.5083347 1.037547  -.702052 .6854889  -.9554881 .6402304  -.6161153 2.022007 |
| No of cases  AMSTAR  Low quality  Moderate quality  Single/Combined miRNAs  cons | -.000159  -.8288856  -.135148  -.089795  .9220901 | .0000478  .3213512  .1461725  .2062041  .2098244 | -3.32  -2.58  -0.92  -0.44  4.39 | 0.001  0.010  0.355  0.663  0.000 | -.0002527 -.0000652  -1.458722 -.1990488  -.4216408 .1513448  -.4939477 .3143577  .5108419 1.333338 |
