# Supplementary material for: Prognostic and diagnostic values of non-coding RNAs as biomarkers for breast cancer: An umbrella review and pan-cancer analysis
Source: Front Mol Biosci. 2023 Jan 16;10:1096524. doi: 10.3389/fmolb.2023.1096524 (PMC9885171; doi:10.3389/fmolb.2023.1096524)
Supplement: Supplementary file 2 [file DataSheet2.ZIP › Supplementary Material, Table 8.docx]

**Supplementary Material, Table 8.** The result of meta-regression of DFS.

| Variables | Coefficient | Std. errs. | z | P>\|z\| | [95% conf. interval] |
| --- | --- | --- | --- | --- | --- |
| One variable at a time |  |  |  |  |  |
| AMSTAR  Low quality  Moderate quality  cons | -.2817121  -.083236  .4060722 | .4707591  .3436422  .2611891 | -0.60  -0.24  1.55 | 0.550  0.809  0.120 | -1.204383 .6409588  -.7567624 .5902903  -.105849 .9179933 |
| lncRNAs/miRNAs  cons | .5099368  -.012971 | .2988833  .2449061 | 1.71  -0.05 | 0.088  0.958 | -.0758637 1.095737  -.4929782 .4670362 |
| Grouped variables |  |  |  |  |  |
| No of cases  AMSTAR  Low quality  Moderate quality  lncRNAs/miRNAs  cons | -.000159  -.8288856  -.135148  -.089795  .9220901 | .0000478  .3213512  .1461725  .2062041  .2098244 | -3.32  -2.58  -0.92  -0.44  4.39 | 0.001  0.010  0.355  0.663  0.000 | -.0002527 -.0000652  -1.458722 -.1990488  -.4216408 .1513448  -.4939477 .3143577  .5108419 1.333338 |
