# Supplementary material for: Prognostic and diagnostic values of non-coding RNAs as biomarkers for breast cancer: An umbrella review and pan-cancer analysis
Source: Front Mol Biosci. 2023 Jan 16;10:1096524. doi: 10.3389/fmolb.2023.1096524 (PMC9885171; doi:10.3389/fmolb.2023.1096524)
Supplement: Supplementary file 2 [file DataSheet2.ZIP › Supplementary Material, Table 9.docx]

**Supplementary Material, Table 9.** The result of meta-regression of RFS.

| Variables | Coefficient | Std. errs. | z | P>\|z\| | [95% conf. interval] |
| --- | --- | --- | --- | --- | --- |
| One variable at a time |  |  |  |  |  |
| AMSTAR  Low quality  Moderate quality  cons | -.8460588  -.3527513  .7544868 | .4537202  .3253989  .214483 | -1.86  -1.08  3.52 | 0.062  0.278  0.000 | -1.735334 .0432164  -.9905215 .2850188  .3341079 1.174866 |
| lncRNAs/miRNAs  cons | .2221261  .4483429 | .284082  .1940635 | 0.78  2.31 | 0.434  0.021 | -.3346645 .7789166  .0679853 .8287004 |
| Grouped variables |  |  |  |  |  |
| AMSTAR  Low quality  Moderate quality  lncRNAs/miRNAs  cons | .1511219  -.8186674  -.3076757  .6804734 | .3303028  .4658061  .3718413  .2707804 | 0.46  -1.76  -0.83  2.51 | 0.647  0.079  0.408  0.012 | -.4962596 .7985035  -1.731631 .0942959  -1.036471 .4211198  .1497535 1.211193 |
