# Supplementary material for: Prognostic and diagnostic values of non-coding RNAs as biomarkers for breast cancer: An umbrella review and pan-cancer analysis
Source: Front Mol Biosci. 2023 Jan 16;10:1096524. doi: 10.3389/fmolb.2023.1096524 (PMC9885171; doi:10.3389/fmolb.2023.1096524)
Supplement: Supplementary file 1 [file Supplementary Material, Fig 15.pdf › Supplementary Material, Fig 15.pdf]

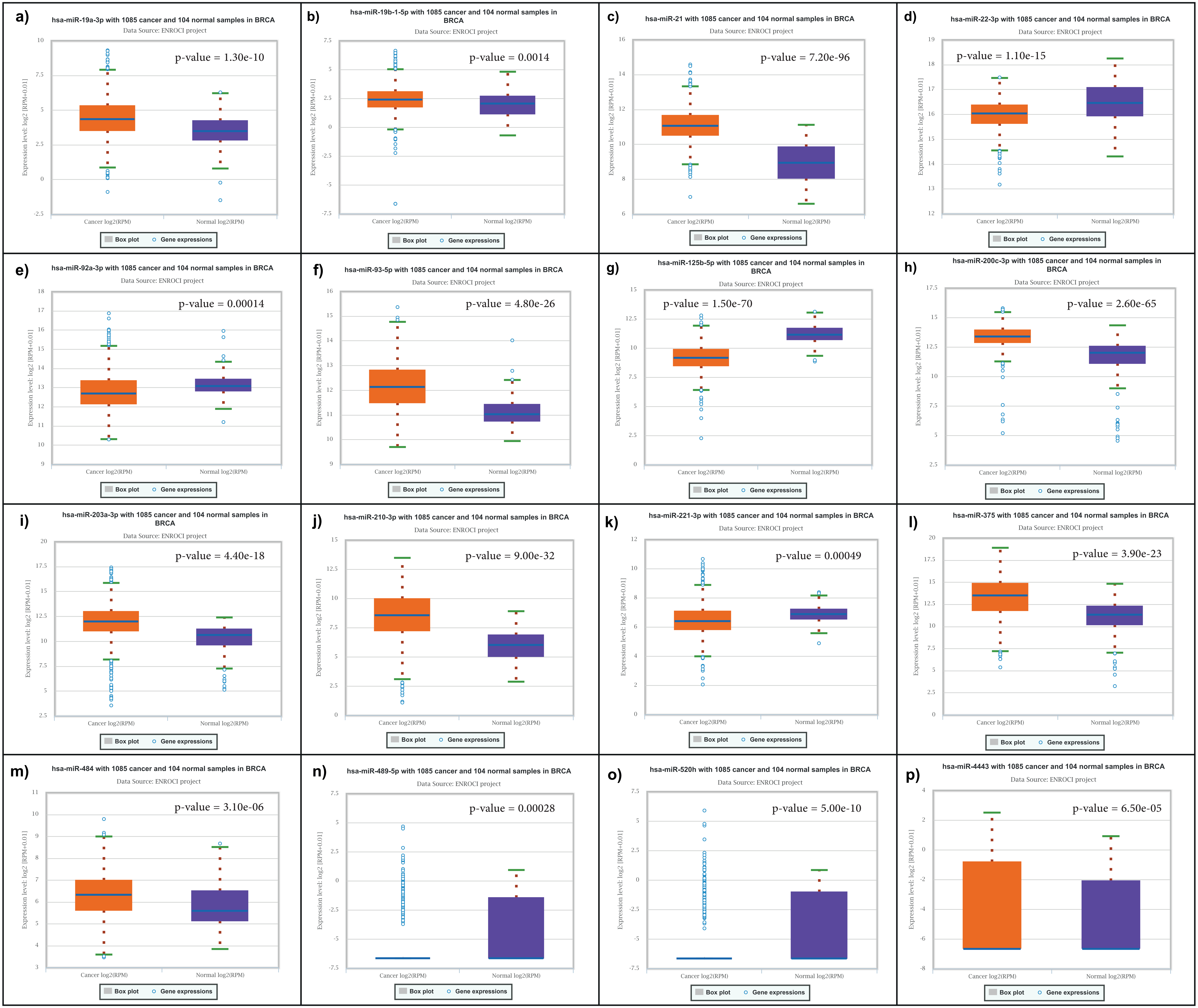
a)
hsa-miR-19a-3p with 1085 cancer and 104 normal samples in BRCA
Data Source: ENROCI project
p-value = 1.30e-10
b)
hsa-miR-19b-1-5p with 1085 cancer and 104 normal samples in BRCA
Data Source: ENROCI project
p-value = 0.0014
c)
hsa-miR-21 with 1085 cancer and 104 normal samples in BRCA
Data Source: ENROCI project
p-value = 7.20e-96
d)
hsa-miR-22-3p with 1085 cancer and 104 normal samples in BRCA
Data Source: ENROCI project
p-value = 1.10e-15
e)
hsa-miR-92a-3p with 1085 cancer and 104 normal samples in BRCA
Data Source: ENROCI project
p-value = 0.00014
f)
hsa-miR-93-5p with 1085 cancer and 104 normal samples in BRCA
Data Source: ENROCI project
p-value = 4.80e-26
g)
hsa-miR-125b-5p with 1085 cancer and 104 normal samples in BRCA
Data Source: ENROCI project
p-value = 1.50e-70
h)
hsa-miR-200c-3p with 1085 cancer and 104 normal samples in BRCA
Data Source: ENROCI project
p-value = 2.60e-65
i)
hsa-miR-203a-3p with 1085 cancer and 104 normal samples in BRCA
Data Source: ENROCI project
p-value = 4.40e-18
j)
hsa-miR-210-3p with 1085 cancer and 104 normal samples in BRCA
Data Source: ENROCI project
p-value = 9.00e-32
k)
hsa-miR-221-3p with 1085 cancer and 104 normal samples in BRCA
Data Source: ENROCI project
p-value = 0.00049
l)
hsa-miR-375 with 1085 cancer and 104 normal samples in BRCA
Data Source: ENROCI project
p-value = 3.90e-23
m)
hsa-miR-484 with 1085 cancer and 104 normal samples in BRCA
Data Source: ENROCI project
p-value = 3.10e-06
n)
hsa-miR-489-5p with 1085 cancer and 104 normal samples in BRCA
Data Source: ENROCI project
p-value = 0.00028
o)
hsa-miR-520h with 1085 cancer and 104 normal samples in BRCA
Data Source: ENROCI project
p-value = 5.00e-10
p)
hsa-miR-4443 with 1085 cancer and 104 normal samples in BRCA
Data Source: ENROCI project
p-value = 6.50e-05
Expression level: log2 [RPM+0.01]
Cancer log2(RPM)
Normal log2(RPM)
Box plot
Gene expressions
